# Global burden of disease due to opioid, amphetamine, cocaine, and cannabis use disorders, 1990-2021: a systematic analysis for the Global Burden of Disease Study 2021

**DOI:** 10.1371/journal.pone.0328276

**Published:** 2025-08-21

**Authors:** David T. Zhu, Ye In Christopher Kwon, Alan Lai, Andrew Min–Gi Park, Andrew J. Barnes, Derek A. Chapman

**Affiliations:** 1 Medical Scientist Training Program, School of Medicine, Virginia Commonwealth University, Richmond, Virginia, United States; 2 Department of Health Policy, School of Public Health, Virginia Commonwealth University, Richmond, Virginia, United States; 3 School of Medicine, Virginia Commonwealth University, Richmond, Virginia, United States; 4 Department of Epidemiology, School of Public Health, Virginia Commonwealth University, Richmond, Virginia, United States; University of New Mexico Health Sciences Center, UNITED STATES OF AMERICA

## Abstract

Drug use disorders (DUDs) represent a major global health challenge, leading to substantial morbidity and mortality, while also being compounded by social and structural barriers. In this study, we examined global epidemiological trends in DUDs over the past three decades to inform clinical and public health responses. We extracted data on the incidence, deaths, and disability-adjusted life years (DALYs) attributable to DUDs from the 2021 Global Burden of Diseases, Injuries, and Risk Factors Study between 1990 and 2021. Age-standardized incidence (ASIR), mortality (ASMR), and DALY rates per 100,000 population were calculated. The analysis focused on four DUDs—opioid, amphetamine, cocaine, and cannabis use disorders—and further stratified rates by sex, Socio-demographic Index (SDI), countries, and world regions. In 2021, there were 13.6 (95% UI, 11.6–15.7) million new cases, 137,278 (95% UI, 129,269–146,181) deaths, and 15.6 (95% UI, 12.8–18.1) million DALYs attributed to DUDs. Between 1990 and 2021, the ASIR decreased by 8.1%, while the ASMR and DALY rates rose by 30.8% and 14.8%, respectively. Opioid use disorder accounted for the highest ASIR (169.4 [95% UI, 145.1–195.0] per 100,000), ASMR (1.7 [95% UI, 1.6–1.8] per 100,000), and age-standardized DALY rate (191.0 [95% UI, 156.1–222.8] per 100,000) in 2021. Sex and geographical variations were notable, with males and world regions like high-income North America, Australasia, and Eastern/Western Europe showing disproportionately higher rates. Overall, these findings highlight rising mortality and morbidity rates despite a modest decline in incidence, underscoring the need for tailored public health interventions, advancing harm reduction programs, and expanding access to treatment.

## Introduction

Drug use disorders (DUDs) are typically defined by patterns of compulsive and persistent drug use [1]. DUDs have been associated with a wide range of psychological and physiological health consequences, including increased risk of overdose, suicidal ideation, infectious diseases like HIV and Hepatitis B and C, and more [2–4]. According to the 2019 Global Burden of Disease, Injuries, and Risk Factors (GBD) Study, DUDs ranked among the top 20 leading causes of disability-adjusted life years (DALYs), accounting for 1.3% of all-cause DALYs globally [5], up from 0.8% in GBD 2010 [6]. Moreover, the World Drug Report 2023 highlights that over 296 million people worldwide have used illicit drugs in 2021, with an estimated 39.5 million individuals suffering from DUDs, a 45% increase over the past decade [7].

In 2019, the United States (U.S.) accounted for more than half of the global DUD-related mortality burden [7], largely due to the overprescribing of opioids in the early 2000s, widespread availability of heroin around 2010, and influx of potent synthetic opioids (e.g., fentanyl) into the illicit drug market beginning around 2013 [8,9]. Additionally, structural barriers continue to hinder access to medication-assisted treatment (MAT) for DUDs, perpetuating overdose disparities, particularly among populations experiencing poverty, homelessness, and comorbid chronic and mental health conditions [10].

Over the past decade, DUDs are increasingly shifting from single-substance use to the comorbid use of multiple substances, known as “polysubstance” use [9,11]. While prescription opioids remain a significant contributor to overdoses, polysubstance mixtures co-involving fentanyl and stimulants like cocaine and methamphetamine now account for the majority of drug overdoses in certain regions like the U.S. and Canada [11]. Thus, this study aims to provide updated and comprehensive estimates of global epidemiological trends using data obtained from GBD 2021. We aim to evaluate temporal and geographical trends in opioid, cocaine, amphetamine, and cannabis use disorders across 204 countries and territories, aiming to inform clinical and public health interventions for preventing, treating, and supporting individuals with DUDs.

## Methods

GBD 2021 methodological details for data collection, analysis, and modeling are thoroughly provided in the GBD 2021 summary publications [12]. Here, we aim to provide a brief overview, focusing on approaches used to estimate DUD-related morbidity and mortality. Given the deidentified and pubicly available data source, this study was exempted from review and informed consent by our university’s institutional review board.

### Data collection

GBD 2021 compiles data from several sources, including vital registration systems, population-based surveys, verbal autopsies, and others, to product robust estimates of morbidity and mortality. These data are standardized to account for differences in case definitions, measurement tools, coding systems (e.g., *ICD* codes), and age-sex distributions. When data are limited or unavailable for specific types of DUDs, regions, or years, GBD employs various models to impute missing data, including the Cause of Death Ensemble Model (CODEm), DisMod-MR 2.1, and Spatiotemporal Gaussian Process Regression (ST-GPR).

We obtained data from the GBD 2021 database on the incidence, mortality, and DALYs attributable to DUDs, both overall and by specific drug type (opioid, amphetamine, cocaine, and cannabis use disorders). One DALYs represents the loss of one “healthy” year due to illness or disability. In GBD 2021, diagnostic criteria for DUDs followed the *Diagnostic and Statistical Manual of Mental Disorders (DSM-IV-TR)* or *the International Classification of Diseases, 10*^*th*^ *revision (ICD-10)*. Mortality data were unavailable for cannabis use disorder in GBD 2021. Additionally, we excluded the aggregated “other drug use disorder” category in GBD 2021 given lack of drug-specific information.

### Data analysis

Age-standardized incidence rates (ASIR), mortality rates (ASMR), and DALYs were calculated using the GBD global standard population and expressed per 100,000 population. The 95% uncertainty intervals (UIs) were derived directly from the GBD 2021 database. Regional and country-specific changes in DUD-related morbidity and mortality were considered significant if the UI excluded the null value of 0%. Additionally, we stratified the data by the 204 countries and territories, as well as five Socio-demographic Index (SDI) quintiles, listed in GBD 2021. The SDI, ranging from 0 to 100, represents socioeconomic development through a composite measure of lag-distributed income per capita (which smooths out short-term income fluctuations by accounting for past income levels), mean years of education for individuals aged 15 and older, and the total fertility rate (the average number of children born to a woman over her lifetime). Methodological details for computing the SDI are provided in S1 Appendix.

### Data visualization

Global chloropleth maps illustrated the total percentage change in DUD-related DALYs from 1990 to 2021. Line plots depicted DALY trends by sex, while tile plots displayed DALY rates by drug type across world regions and SDI quintiles in 2021. The full list of these 26 world regions are listed in S1 Appendix.

## Results

### Drug use disorders, incidence

In 2021, there were 13.6 (95% UI, 11.6–15.7) million new cases of DUD reported across 204 countries and territories, representing a 35.5% increase from 1990 (10.0 [95% UI, 8.5–11.5] million) (Table 1). From 1990 to 2021, the number of new cases rose for opioid use disorder (+49.2%), cannabis use disorder (+27.2%), and cocaine use disorder (+17.2%), while decreasing for amphetamine use disorder (−21.6%). Notably, cannabis use disorder had the highest incidence in 2021, accounting for 3.6 (95% UI, 2.7–4.7) million new cases.

**Table 1:** Global incidence, deaths, and DALYs attributable to drug use disorders in 1990, 2000, 2010, and 2021.

Measure	1990	2000	2010	2021
**Any drug use disorder** ^**1**^				
Incidence, N (95% UI)	10,043,456 (8,541,086– 11,526,400)	11,523,701 (9,872,473– 13,179,331)	12,476,821 (10,724,615– 14,248,840)	13,609,362 (11,625,288– 15,667,184)
Incidence rate per 100,000 (95% UI)	184.3 (156.9– 211.7)	181.5 (155.3– 207.7)	170.8 (146.8– 195.9)	169.4 (145.1– 195.0)
Deaths, N (95% UI)	61,774 (57,329– 66,898)	82,895 (79,383– 86,474)	89,468 (87,049– 91,816)	137,278 (129,269– 146,181)
Death rate per 100,000 (95% UI)	1.3 (1.2–1.4)	1.4 (1.3–1.5)	1.3 (1.2–1.3)	1.7 (1.6–1.8)
DALYs, N (95% UI)	8,910,603 (7,055,603– 10,630,912)	11,070,351 (9,041,838– 13,076,196)	11,616,942 (9,484,737– 13,741,902)	15,562,162 (12,752,222– 18,119,264)
DALY rate per 100,000 (95% UI)	166.4 (132.6– 198.4)	177.2 (145.1– 208.6)	159.7 (130.5– 188.5)	191.0 (156.1– 222.8)
**Opioid use disorder**				
Incidence, N (95% UI)	1,301,551 (1,077,634– 1,598,053)	1,632,978 (1,369,018– 1,985,698)	1,637,140 (1,376,840– 1,982,929)	1,942,525 (1,643,342– 2,328,363)
Incidence rate per 100,000 (95% UI)	23.4 (19.6–28.5)	25.7 (21.7–31.2)	22.2 (18.7–26.8)	24.5 (20.7–29.5)
Deaths, N (95% UI)	41,567 (36,923– 45,060)	56,264 (53,672– 58,853)	63,749 (61,807– 65,583)	99,555 (92,948– 108,050)
Death rate per 100,000 (95% UI)	0.9 (0.8–0.9)	1.0 (0.9–1.0)	0.9 (0.9–0.9)	1.2 (1.1–1.3)
DALYs, N (95% UI)	5,415,249 (4,242,001– 6,437,812)	7,129,063 (5,673,767– 8,453,675)	7,826,828 (6,299,231– 9,289,910)	11,218,519 (9,188,658– 13,159,551)
DALY rate per 100,000 (95% UI)	103.7 (81.8, 122.8)	115.1 (91.8, 135.8)	108.0 (87.1, 128.1)	137.1 (112.3, 161.4)
**Amphetamine use disorder**				
Incidence, N (95% UI)	1,364,378 (943,695– 1,909,687)	1,282,385 (908,915– 1,790,758)	1,139,242 (790,516– 1,596,663)	1,069,011 (757,797–1,482,514)
Incidence rate per 100,000 (95% UI)	22.7 (15.9–31.8)	19.6 (13.9–27.3)	15.2 (10.6–21.3)	13.7 (9.7–19.1)
Deaths, N (95% UI)	4,852 (4,108– 5,815)	6,055 (5,509–6,697)	6,162 (5,900–6,454)	9,880 (8920– 11,061)
Death rate per 100,000 (95% UI)	0.1 (0.1–0.1)	0.1 (0.1–0.1)	0.1 (0.1–0.1)	0.1 (0.1–0.1)
DALYs, N (95% UI)	1,705,357 (1,110,942– 2,494,977)	1,772,502 (1,169,867– 2,574,333)	1,541,609 (1,021,449– 2,231,532)	1,677,367 (1,171,027– 2,343,858)
DALY rate per 100,000 (95% UI)	29.6 (19.5–43.5)	27.5 (18.2–39.9)	20.9 (13.9–30.0)	21.0 (14.6–29.3)
**Cocaine use disorder**				
Incidence, N (95% UI)	186,500 (128,306– 266,812)	210,779 (150,153– 290,525)	233,740 (168,521– 317,740)	219,145 (158,098– 298,957)
Incidence rate per 100,000 (95% UI)	3.1 (2.1–4.4)	3.1 (2.3–4.3)	3.2 (2.3–4.4)	2.9 (2.1–3.9)
Deaths, N (95% UI)	3,538 (2,966–4,486)	6,146 (5,502–7,029)	7,688 (7,159–8,375)	12,555 (11,406– 14,508)
Death rate per 100,000 (95% UI)	0.1 (0.1–0.1)	0.1 (0.1–0.1)	0.1 (0.1–0.1)	0.2 (0.1–0.2)
DALYs, N (95% UI)	583,791 (416,443– 801,039)	769,420 (583,148– 1,009,434)	900,775 (700,908– 1,165,241)	1,133,624 (917,462– 1,427,944)
DALY rate per 100,000 (95% UI)	10.9 (7.9–14.8)	12.4 (9.4–16.2)	12.4 (9.7–16.0)	13.9 (11.2–17.5)
**Cannabis use disorder** ^**3**^				
Incidence, N (95% UI)	2,849,696 (2,126,950– 3,746,333)	3,120,769 (2,379,633– 4,062,689)	3,451,669 (2,642,221– 4,518,813)	3,625,507 (2,738,090– 4,737,132)
Incidence rate per 100,000 (95% UI)	48.5 (36.4–63.4)	47.1 (36.1–61.1)	47.2 (35.9–61.2)	46.8 (35.3–61.2)
DALYs, N (95% UI)	491,999 (287,638– 764,349)	540,390 (317,958– 830,324)	612,185 (361,162– 951,794)	653,849 (389,510– 1,017,565)
DALY rate per 100,000 (95% UI)	8.7 (5.1–13.3)	8.4 (5.0–12.9)	8.6 (5.1–13.3)	8.3 (4.9–12.9)

**Abbreviations.** 95% UI=95% uncertainty interval; DALY=disability-adjusted life years.

^1^Any drug use disorder encompasses opioid, amphetamine, cocaine, and cannabis use disorders, and other drug use disorders. Given the lack of specificity regarding the specific drug types included in the aggregated ‘other drug use disorder’ category in GBD 2021, this variable was excluded from the present study.“

^2^Mortality data unavailable for cannabis use disorder in GBD 2021.

**Fig 1:**
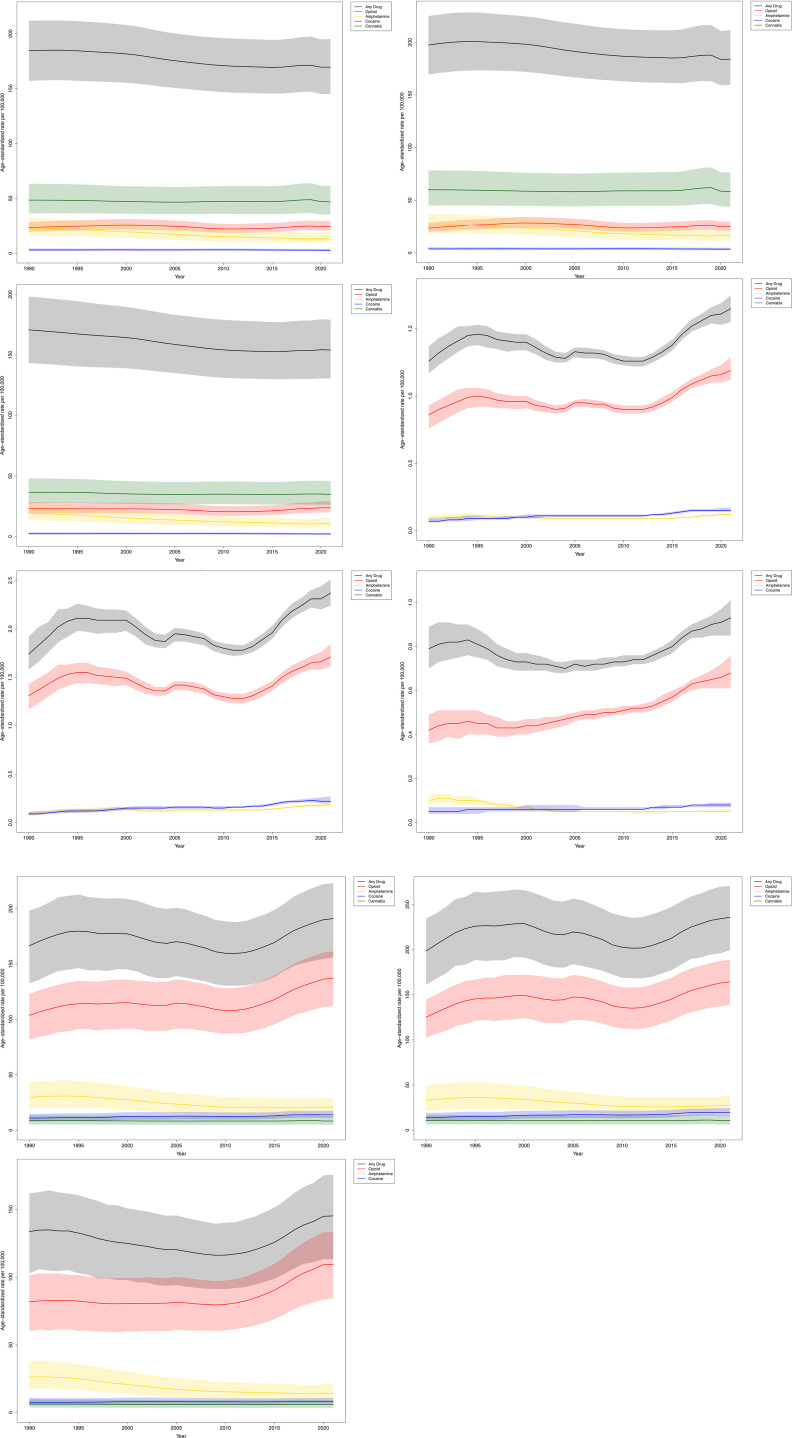
Global age-standardized rates of incidence, mortality, and DALYs due to drug use disorders overall and by sex, 1990–2021.

Conversely, the ASIR for any DUD decreased by 8.1%, from 184.3 (95% UI, 156.9–211.7) per 100,000 in 1990 to 169.4 (95% UI, 145.1–195.0) per 100,000 in 2021 (Table 1, Fig 1, S1 Table 1). The ASIR rose slightly for opioid use disorder (+4.7%), but decreased for amphetamine use disorder (−39.6%), cocaine use disorder (−6.5%), and cannabis use disorder (−3.5%). Cannabis use disorder continued to have the highest ASIR in 2021, with 46.8 (95% UI, 35.3–61.2) new cases per 100,000.

### Drug use disorders, mortality

In 2021, there were 137,278 (95% UI, 129,269−146,181) deaths attributed to DUD, marking a 122.2% increase from 1990 (61,774 [95% UI, 57,329−66,898]) (Table 1). Deaths increased most for cocaine use disorder (+254.9%), followed by opioid use disorder (+139.5%) and amphetamine use disorder (+103.6%). Opioid use disorder was responsible for the highest number of deaths in 2021, accounting for 99,555 (95% UI, 92,948−108,050) fatalities globally.

Similarly, the ASMR rose by 30.8% between 1990 (1.3 [95% UI, 1.2–1.4] per 100,000) and 2021 (1.7 [95% UI, 1.6–1.8] per 100,000) (Table 1, Fig 1, S2 Table). During this period, the ASMR increased for cocaine use disorder (+100.0%) and opioid use disorder (+33.3%), while remaining relatively stable for amphetamine use disorder. In 2021, opioid use disorder had the highest ASMR, with 24.5 (95% UI, 20.7–29.5) deaths per 100,000.

### Drug use disorders, DALYs

In 2021, DUDs accounted for 15.6 (95% UI, 12.8–18.1) million DALYs, reflecting a 74.6% increase from 1990 (8.9 [95% UI, 7.1–10.6] million) (Table 1). DALYs rose for opioid use disorder (+107.2%), cocaine use disorder (+94.2%), and cannabis use disorder (+32.9%), but decreased for amphetamine use disorder (−1.6%).

The age-standardized DALY rate increased by 14.8% over this period, from 166.4 (95% UI, 132.6–198.4) per 100,000 in 1990 to 191.0 (95% UI, 156.1–222.8) per 100,000 in 2021 (Table 1, Fig 1, S3 Table). Rates rose for opioid use disorder (+32.2%) and cocaine use disorder (+27.5%) but fell for amphetamine use disorder (−29.1%) and cannabis use disorder (−4.6%). In 2021, opioid use disorder had the highest DALY rate, at 137.1 (95% UI, 112.3–161.4) per 100,000.

### Drug use disorders, sex differences

These trends also differed by sex. In 2021, the ASIR for DUD was 19.5% higher among males (184.0 [95% UI, 159.7–211.5] per 100,000) compared to females (154.1 [95% UI, 130.5–179.2] per 100,000) (Fig 1, S1 Table). Between 1990 and 2021, the ASIR decreased for both sexes, but the decline was more pronounced in females (−9.7%) than in males (−6.8%).

Similarly, in 2021, the ASMR among males (2.4 [95% UI, 2.2–2.5] per 100,000) was more than double that of females (0.9 [95% UI, 0.9–1.0] per 100,000) (Fig 1, S2 Table). Between 1990 and 2021, the increase in ASMR among males (+36.2%) was roughly double that of females (+17.7%).

DALYs also varied considerably by sex (Fig 1, S3 Table). In 2021, the age-standardized DALY rate was 62.4% higher among males (235.9 [95% UI, 200.0–270.3] per 100,000) than among females (145.3 [95% UI, 113.4–175.6] per 100,000). Between 1990 and 2021, these rates increased more for males (+18.8%) than for females (+8.6%).

### Drug use disorders, geographical variations

**Fig 2:**
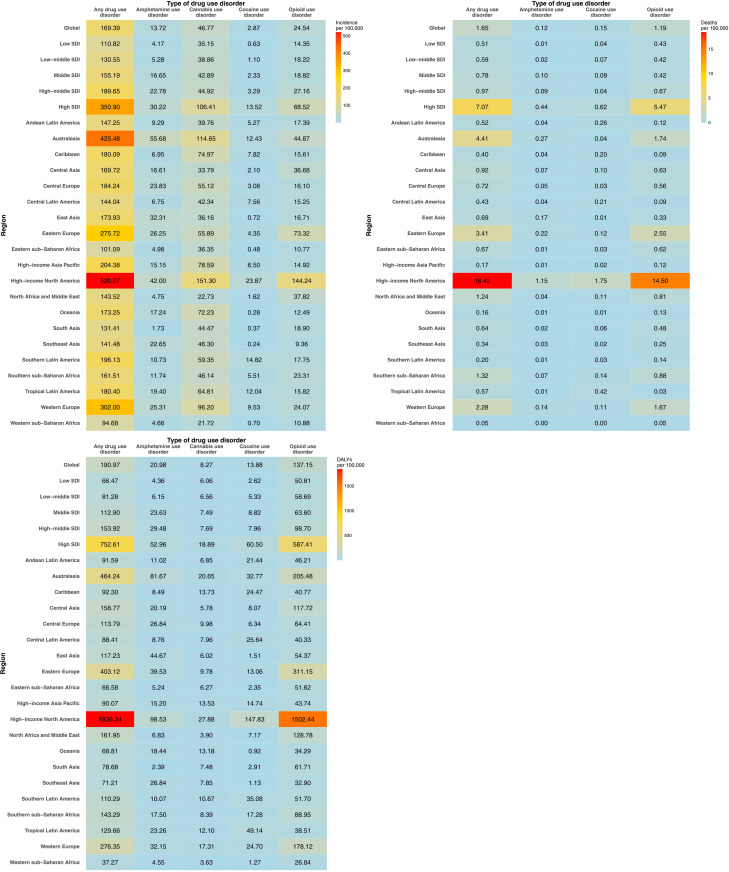
Global age-standardized rates of incidence, mortality, and disability-adjusted life years (DALYs) attributable to drug use disorders, by region, in 2021.

Epidemiological trends in DUD reveal notable geographical variations. In 2021, ASIRs for overall DUD were highest in the high SDI quintile (350.9 [95% UI, 307.4–400.2] per 100,000), which was more than double the global ASIR rate (169.4 [95% UI, 145.1–195.0] per 100,000) (Fig 2, S4 Table). High-income North America (520.1 [95% UI, 454.1–592.8] per 100,000), Australasia (425.5 [95% UI, 369.4–483.0] per 100,000), and Western Europe (302.0 [95% UI, 262.9–348.2] per 100,000) showed disproportionately higher rates.

Similarly, ASMRs were highest in the high SDI quintile (7.1 [95% UI, 6.5–7.7] per 100,000), more than four times the global ASMR (1.7 [95% UI, 1.6–1.8] per 100,000) (Fig 2, S5 Table). In high-income North America, the ASMR (18.4 [95% UI, 16.8–20.3] per 100,000) was approximately 11.4 times the global ASMR, while high rates were also observed in Australasia (4.4 [95% UI, 3.9–4.9] per 100,000) and Eastern Europe (3.4 [95% UI, 3.1–3.7] per 100,000).

**Fig 3:**
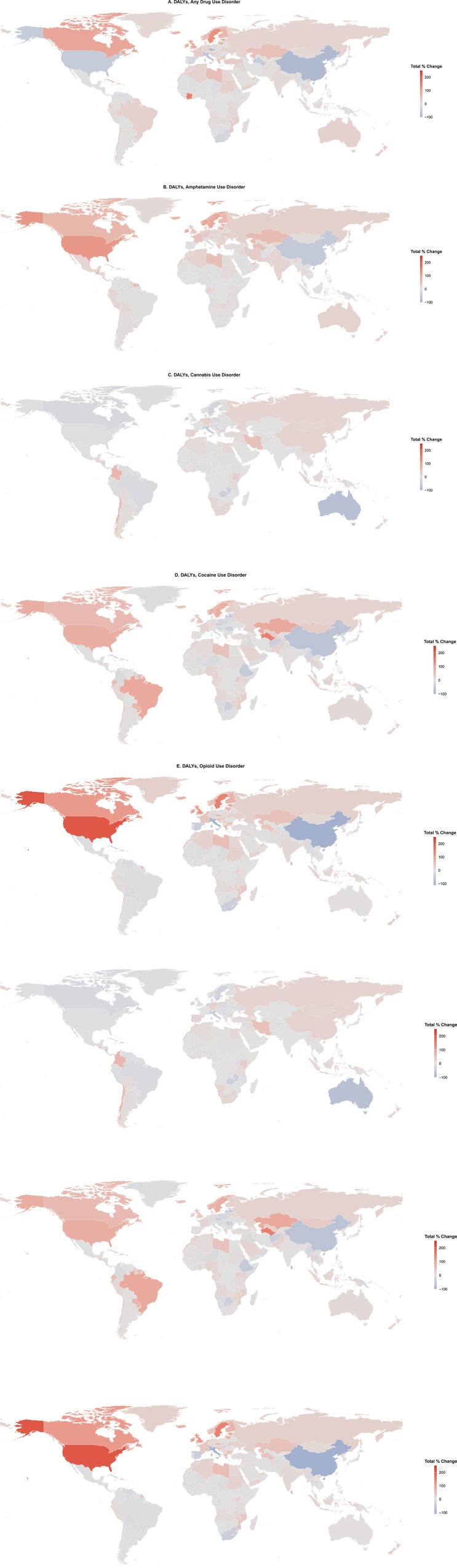
Percentage changes between 1990 and 2021 age-standardized DALY rates per 100,000 for drug use disorders, by drug type and country.

The age-standardized rate of DALYs for DUD was highest in the high SDI quintile (752.6 [95% UI, 630.6–872.9] per 100,000), nearly four times the global rate (191.0 [95% UI, 156.1–222.8] per 100,000) (Fig 2, S6 Table). These rates were disproportionately higher in high-income North America (1836.3 [95% UI, 1547.7–2122.5] per 100,000), roughly 2.4 times the global rate. Although Australasia (464.2 [95% UI, 387.4–539.8] per 100,000) and Eastern Europe (403.1 [95% UI, 387.4–539.8] per 100,000) also had notably elevated rates, they were lower compared to the global rate. Between 1990 and 2021, DALY rates for DUD increased in 137 countries (67.2%) and decreased in 67 countries (32.8%) (Fig 3, S7 Table).

In 2021, among specific DUDs, cannabis use disorder had the highest global ASIR (46.8 [95% UI, 35.3–61.2] per 100,000), while opioid use disorder had the highest ASMR (1.2 [95% UI, 1.1–1.3] per 100,000) and age-standardized DALY rate (137.2 [95% UI, 112.3–161.4] per 100,000) (Table 1, Fig 2). The U.S. exhibited the highest DALY rate for opioid use disorder (1594.6 [95% UI, 1308.1–1849.8] per 100,000), followed by Canada (681.0 [95% UI, 590.5–775.9] per 100,000) and Estonia (611.1 [95% UI, 481.1–739.6] per 100,000) (Fig 3, S8 Table). Opioid use disorder DALYs rose in 107 countries (52.5%) and decreased in 97 countries (47.6%).

For stimulant use disorders, DALY rates for amphetamine use disorder were highest in the U.S. (103.9 [95% UI, 84.0–129.9] per 100,000), followed by Australia (83.5 [95% UI, 55.2–120.3] per 100,000) and New Zealand (72.3 [95% UI, 44.1–106.2] per 100,000) (Fig 3, S9 Table). Similarly, DALY rates for cocaine use disorder were highest in the U.S. (70.2 [95% UI, 47.0–101.1] per 100,000), followed by Greenland (49.0 [95% UI, 31.9–75.2] per 100,000) and Canada (46.0 [95% UI, 30.1–71.4] per 100,000). DALY rates for amphetamine use disorder rose in 164 countries (80.4%) and fell in 37 countries (19.6%), while for cocaine use disorder, they increased in 122 countries (59.8%) and decreased in 82 countries (40.2%) (Fig 3, S10 Table).

Finally, DALY rates for cannabis use disorder were highest in Australia (37.0 [95% UI, 24.1–54.3] per 100,000), followed by Canada (35.8 [95% UI, 23.5–52.8] per 100,000) and the U.S. (28.9 [95% UI, 17.2–44.9] per 100,000) (Fig 3, S11 Table). These rates rose in 120 countries (58.8%) and declined in 84 countries (41.2%).

## Discussion

This cross-sectional study provides an updated and comprehensive analysis of spatial and temporal patterns attributed to DUDs between 1990 and 2021. Notably, our study findings show an overall decline in DUD-related ASIRs over the study period; while amphetamine, cocaine, and cannabis use disorders showed a decreasing trend, we observed a slight increase for opioid use disorder. In contrast, DUD-related ASMR and DALYs consistently increased over the study period, predominantly driven by rising rates for opioid use disorder. Males consistently exhibited higher rates of DUDs, and geographical variations were evident, with higher rates in high-income North America (e.g., the U.S. and Canada) and Australasia. Overall, opioid use disorder was the largest contributor to ASMRs and DALYs in high-income North America, while cannabis use disorder contributed most to DALYs in Australasia.

Consistent with prior empirical evidence [5], our findings show that more than half of global DUD-related deaths occurred in the U.S., with approximately three out of four deaths from opioid use disorder. The rise in opioid overdose deaths has been driven by the widespread proliferation of illicitly manufactured fentanyl, a potent synthetic opioid spread through both international trafficking routes and domestic clandestine drug laboratories, as well as a smaller contribution from diverted pharmaceutical supplies [13]. Additionally, fentanyl and its analogs are often mixed with other opioids (e.g., heroin) and stimulants (e.g., cocaine and methamphetamine) in polysubstance mixtures, and this evolving landscape poses significant challenges to implement targeted public health interventions [14].

Numerous interventions have been implemented to combat the rising global burden of opioid use disorder. Prior evidence demonstrates that the broad distribution of harm reduction resources such as naloxone is associated with a significant reduction in overdose deaths [15]. However, considering our study findings showing rising global ASMRs and DALYs for opioid use disorder, further preventative efforts are needed to mitigate harms surrounding opioid use. These may included, but are not limited to, proactive screening for DUDs to inform clinical management, more accessible drug testing services, and enhanced epidemiological surveillance to monitor drug overdoses [10,16]. Further policies are also needed to address the underlying social determinants of health that increase vulnerability to DUDs, including disparities by socioeconomic status, access to addiction treatment and mental health services, and environmental and neighborhood factors [10,16].

In contast, amphetamine use disorder has shown declining ASIRs and DALY rates, with ASMRs remaining fairly stable over time; this is consistent with GBD 2019 findings [1]. The observed decline in ASIRs may be partially explained by the under-diagnosis of methamphetamine use disorder, given that methamphetamine use is highly stigmatized, leading to underreporting and delays in seeking treatment [17], For instance, an Australian study found that the level of under-reporting was strongly associated with negative attitudes towards methamphetamine use, with actual prevalence being approximately four-fold higher than estimated [18]. These public attitude shifts may be fueled by negative media stories associating amphetamine use with violence and crime, as well as movements such as the Australian government’s “Ice Destroys Lives” campaign, which further stigmatizes methamphetamine use [19].

Nevertheless, the global burden of amphetamine use disorder remains substantial and underscores the need for further clinical and public health interventions for stimulant use disorders. This is particularly salient given the well-documented challenges in clinical trials for stimulant use disorder medications, which are often hindered by smaller sample sizes, inconsistency in design and outcome assessments, and high attrition rates [20]. Additionally, available psychosocial interventions (e.g., cognitive behavioral therapy, contingency management, and community reinforcement) have shown limited effectiveness in treating amphetamine use disorder and are variably implemented [21]. The limited availability of these psychosocial interventions also remains a significant barrier to accessing treatment among individuals with stimulant use disorders [22]. Further research is needed to develop effective pharmacological therapies for amphetamine use disorder, while simultaneously ensuring the accessibility and affordability of these treatments.

Despite a slight decline in ASIRs for cocaine use disorder, the number of deaths has tripled over the past three decades and the ASMR has doubled. This increase is likely influenced by a combination of factors, including the production of cocaine in clandestine laboratories and co-involvement of cocaine with fentanyl in polysubstance mixtures [9,11,27]. Prior evidence suggests that approximately three out of four cocaine-related overdose deaths in the U.S. co-involved at least one opioid in 2018 [23]. This pattern appears to have been amplified in recent years where overdose deaths co-involving cocaine and opioids decreased by 12% per year from 2006 to 2010, remained stable from 2010 to 2014, but only to increase by 46% from 2014 to 2017 [24]. These findings underscore the need for multi-sectoral partnerships to improve overdose prevention efforts for stimulant overdoses, including expanding access to drug testing and reducing stigma for harm reduction clinics and overdose prevention centers. Community-based harm reduction programs have been shown to significantly decrease overdose deaths, are cost-effective, and promote greater treatment engagement without increasing crime or public disorder in surrounding regions [25].

Cannabis use disorders continue to have the highest number of incident cases and ASIRs compared to other DUDs, particularly in high-income North America, Australasia, and Western Europe. The increasing ASIRs may be partly explained by increasing evidence demonstrating the medical benefits of cannabis, particularly for chronic pain, resulting in greater access for medical and recreational use [26]. Prior evidence shows that cannabis legalization has been positively associated with increased prevalence of illicit cannabis use and cannabis use disorders [27]. North America was one of the first regions to allow cannabis usage for medical purposes, with Canada doing so in 2001 and the majority of U.S. states following suit [28]. Similarly, Australia enacted policies allowing access to medical cannabis in 2016, following significant public support, which increased the prevalence of cannabis use disorder [29].

As more countries legalize cannabis, its potential for dependence and adverse outcomes could rise, emphasizing the need for continued monitoring and harm reduction-informed preventive measures [28]. Policymakers could consider regulatory frameworks that limit tetrahydrocannabinol (THC) concentrations in recreational cannabis products to mitigate the risk of dependence and psychosis, which have been linked to higher THC potency [27,28]. Additionally, public education campaigns to educate the public about the risks of heavy cannabis use and promoting safe use behaviors are essential. Ultimately, ensuring that individuals with cannabis use disorder receive a comprehensive continuum of care will be critical in addressing its global burden, including pharmacotherapy, behavioral treatment, and psychosocial support.

### Limitations

This study has several limitations. First, the GBD 2021 database relies solely on *DSM-IV-TR* and *ICD-10* diagnostic criteria for DUDs. Compared to *DSM-V* criteria, these case definitions may lead to an underestimation of DUD-related morbidity and mortality attributed, as *DSM-IV* and *ICD-10* separate harmful substance use from substance dependence; in contrast, *DSM-V* employs broader criteria, classifying individuals with mild, moderate, or severe substance use disorder based on the number of diagnostic criteria met [30]. Second, given that many individuals who use drugs have an overdose without ever receiving a formal DUD diagnosis, our estimates may not capture the full scope of drug-related hospitalizations or deaths. Third, aside from the SDI quintiles, the GBD 2021 database does not include information on key social determinants of health and clinical factors which have been widely shown to influence DUD-related morbidity, including income, education, race and ethnicity, insurance, housing instability, healthcare access, and comorbid conditions. Thus, further studies are needed to stratify global epidemiological trends in DUDs by such factors.

## Conclusion

Overall, our study findings show that DUDs contribute to a substantial global burden of DUD-related morbidity and mortality; this is particularly concerning for opioid and stimulant use disorders. Despite progress in expanding access to medication-assisted therapies, rising mortality and DALY rates underscore an urgent need for tailored public health interventions to expand access to drug testing, public health surveillance, addiction treatment, and social services. Addressing these challenges requires a coordinated global effort to mitigate the escalating global burden of DUDs.

## Supporting information

S1 Appendix Global Burden of Disease Study 2021 methodological details. (DOCX)

S1 Table Global age-standardized incidence rates (ASIR) per 100,000 attributable to any, opioid, amphetamine, cocaine, and cannabis use disorders, 1990–2021. (DOCX)

S2 Table Global age-standardized mortality rates (ASMR) per 100,000 attributable to any, opioid, amphetamine, cocaine, and cannabis use disorders, 1990–2021. (DOCX)

S3 Table Global age-standardized disability-adjusted life year (DALY) rates per 100,000 attributable to any, opioid, amphetamine, cocaine, and cannabis use disorders, 1990–2021. (DOCX)

S4 Table Age-standardized incidence rates (ASIRs) per 100,000 attributable to any, opioid, amphetamine, cocaine, and cannabis use disorders, stratified by world region, 1990–2021. (DOCX)

S5 Table Age-standardized mortality rates (ASMRs) per 100,000 attributable to any, opioid, amphetamine, cocaine, and cannabis use disorders, stratified by world region, 1990–2021. (DOCX)

S6 Table Age-standardized disability-adjusted life year (DALY) rates per 100,000 attributable to any, opioid, amphetamine, cocaine, and cannabis use disorders, stratified by world region, 1990–2021. (DOCX)

S7 Table Age-standardized disability-adjusted life year (DALY) rates per 100,000 attributable to any drug use disorder, stratified by country in 1990 and 2021, and total percentage change. (DOCX)

S8 Table Age-standardized disability-adjusted life year (DALY) rates per 100,000 attributable to any opioid use disorder, stratified by country in 1990 and 2021, and total percentage change. (DOCX)

S9 Table Age-standardized disability-adjusted life year (DALY) rates per 100,000 attributable to any amphetamine use disorder, stratified by country in 1990 and 2021, and total percentage change. (DOCX)

S10 Table Age-standardized disability-adjusted life year (DALY) rates per 100,000 attributable to any cocaine use disorder, stratified by country in 1990 and 2021, and total percentage change. (DOCX)

S11 Table Age-standardized disability-adjusted life year (DALY) rates per 100,000 attributable to any cocaine use disorder, stratified by country in 1990 and 2021, and total percentage change. (DOCX)
