# Supplementary material for: Global burden of disease due to opioid, amphetamine, cocaine, and cannabis use disorders, 1990-2021: a systematic analysis for the Global Burden of Disease Study 2021
Source: PLoS One. 2025 Aug 21;20(8):e0328276. doi: 10.1371/journal.pone.0328276 (PMC12370144; doi:10.1371/journal.pone.0328276)
Supplement: S1 Appendix — (DOCX) [file pone.0328276.s001.docx]

**eMethods**

**Section 1. Calculation of the Socio-Demographic Index**

Using data from the Global Burden of Diseases, Injuries, and Risk Factors (GBD) 2021 Study, we obtained estimates of age-standardized incidence (ASIR), mortality (ASMR), and DALYs for drug use disorders (DUDs) based on Socio-demographic Index (SDI) quintiles. The SDI is a composite measure ranging from 0 to 100, representing the socioeconomic conditions at each specific location-year. Specifically, SDI is a geometric mean of the total fertility rate for individuals aged 25 years or under (TFU25), mean educational attainment for individuals aged 15 years or above (EA15), and lag-distributed income per capita (LDI). The index scores (I) for calculating SDI is defined by the following equation:

$I_{Cly} = \frac{C_{ly} - C_{low}}{C_{high} - C_{low}}$

I_Cly_ denotes the index score for covariate *C*, location *l*, and year *y*. Note that for I_TFU25_, the index score is calculated using the modified equation below, as lower TFU25 values indicate higher levels of development, resulting in higher index scores.

$I_{Cly} = 1 - \frac{C_{ly} - C_{low}}{C_{high} - C_{low}}$

Using these index scores, the SDI can be calculated using the following equation:

$SDI= \sqrt[3]{I_{TFU25}* I_{EA15}* I_{lnLDI}}$ * 100

**Section 2. World Regions in GBD 2021**

We obtained estimates of age-standardized disability-adjusted life years (DALYs) for DUDs for 204 countries and territories, as well as several major world regions. These regions include Andean Latin America; Australasia; Caribbean; Central Asia; Central Europe; Central Latin America; East Asia; Eastern Europe; Eastern sub-Saharan Africa; high-income Asia Pacific; high-income North America; North Africa and Middle East; Oceania; South Asia; Southeast Asia; Southern Latin America; Southern sub-Saharan Africa; Tropical Latin America; Western Europe; and Western sub-Saharan Africa.
