# Supplementary material for: Global burden of disease due to opioid, amphetamine, cocaine, and cannabis use disorders, 1990-2021: a systematic analysis for the Global Burden of Disease Study 2021
Source: PLoS One. 2025 Aug 21;20(8):e0328276. doi: 10.1371/journal.pone.0328276 (PMC12370144; doi:10.1371/journal.pone.0328276)
Supplement: S1 Table — (DOCX) [file pone.0328276.s002.docx]

**S1 Table. Global age-standardized incidence rates (ASIR) per 100,000 attributable to any, opioid, amphetamine, cocaine, and cannabis use disorders, 1990-2021**

| **Year** | **Any drug use disorder, ASIR (95% UI)** | **Opioid use disorder, ASIR (95% UI)** | **Amphetamine use disorder, ASIR (95% UI)** | **Cocaine use disorder, ASIR (95% UI)** | **Cannabis use disorder, ASIR (95% UI)** |
| --- | --- | --- | --- | --- | --- |
| **Both sexes** | | | | | |
| 1990 | 184.31 (156.91, 211.67) | 23.37 (19.58, 28.48) | 22.7 (15.92, 31.75) | 3.09 (2.13, 4.39) | 48.46 (36.39, 63.37) |
| 1991 | 184.65 (157.33, 211.82) | 23.73 (19.9, 28.9) | 22.75 (16.01, 31.67) | 3.08 (2.14, 4.32) | 48.43 (36.45, 63.26) |
| 1992 | 184.85 (157.54, 211.86) | 24.06 (20.19, 29.27) | 22.67 (16.01, 31.45) | 3.08 (2.15, 4.29) | 48.4 (36.5, 63.16) |
| 1993 | 184.87 (157.59, 211.86) | 24.34 (20.45, 29.58) | 22.5 (15.91, 31.12) | 3.08 (2.16, 4.27) | 48.35 (36.54, 63.06) |
| 1994 | 184.69 (157.44, 211.66) | 24.57 (20.66, 29.82) | 22.23 (15.75, 30.74) | 3.09 (2.17, 4.27) | 48.28 (36.54, 62.93) |
| 1995 | 184.28 (157.08, 211.24) | 24.72 (20.82, 29.99) | 21.87 (15.51, 30.24) | 3.1 (2.18, 4.27) | 48.16 (36.5, 62.77) |
| 1996 | 183.75 (156.74, 210.7) | 24.9 (20.99, 30.2) | 21.41 (15.21, 29.65) | 3.11 (2.19, 4.29) | 47.99 (36.39, 62.49) |
| 1997 | 183.24 (156.42, 210.14) | 25.14 (21.21, 30.5) | 20.89 (14.88, 28.96) | 3.13 (2.21, 4.31) | 47.77 (36.32, 62.1) |
| 1998 | 182.74 (156.11, 209.54) | 25.41 (21.43, 30.84) | 20.38 (14.53, 28.24) | 3.15 (2.24, 4.33) | 47.54 (36.25, 61.68) |
| 1999 | 182.18 (155.74, 208.65) | 25.61 (21.61, 31.1) | 19.93 (14.2, 27.67) | 3.15 (2.25, 4.33) | 47.31 (36.18, 61.29) |
| 2000 | 181.51 (155.25, 207.67) | 25.68 (21.67, 31.19) | 19.56 (13.93, 27.26) | 3.14 (2.25, 4.3) | 47.13 (36.13, 61.05) |
| 2001 | 180.55 (154.63, 206.58) | 25.6 (21.6, 31.09) | 19.2 (13.65, 26.85) | 3.12 (2.23, 4.26) | 46.98 (36, 60.89) |
| 2002 | 179.26 (153.66, 205.02) | 25.43 (21.44, 30.86) | 18.74 (13.31, 26.28) | 3.11 (2.23, 4.22) | 46.85 (35.89, 60.73) |
| 2003 | 177.83 (152.42, 203.06) | 25.2 (21.25, 30.55) | 18.24 (12.92, 25.64) | 3.1 (2.22, 4.19) | 46.74 (35.79, 60.59) |
| 2004 | 176.46 (151.37, 201.42) | 24.93 (21.04, 30.2) | 17.76 (12.53, 24.99) | 3.1 (2.23, 4.18) | 46.65 (35.72, 60.49) |
| 2005 | 175.29 (150.58, 200.22) | 24.65 (20.8, 29.83) | 17.32 (12.18, 24.39) | 3.11 (2.24, 4.17) | 46.6 (35.68, 60.4) |
| 2006 | 174.28 (149.72, 199.06) | 24.24 (20.45, 29.31) | 16.88 (11.85, 23.7) | 3.12 (2.25, 4.19) | 46.63 (35.67, 60.44) |
| 2007 | 173.25 (148.85, 197.93) | 23.65 (19.95, 28.57) | 16.37 (11.46, 22.95) | 3.14 (2.27, 4.23) | 46.77 (35.73, 60.63) |
| 2008 | 172.29 (148.02, 197.02) | 23.02 (19.42, 27.79) | 15.88 (11.09, 22.21) | 3.17 (2.3, 4.29) | 46.96 (35.83, 60.87) |
| 2009 | 171.45 (147.29, 196.45) | 22.5 (18.97, 27.16) | 15.47 (10.78, 21.61) | 3.19 (2.31, 4.33) | 47.11 (35.9, 61.09) |
| 2010 | 170.78 (146.75, 195.88) | 22.22 (18.74, 26.81) | 15.2 (10.59, 21.25) | 3.2 (2.31, 4.35) | 47.17 (35.92, 61.19) |
| 2011 | 170.3 (146.33, 195.43) | 22.17 (18.72, 26.74) | 15.01 (10.46, 20.93) | 3.18 (2.3, 4.33) | 47.15 (35.87, 61.19) |
| 2012 | 169.95 (146.05, 195.08) | 22.27 (18.79, 26.84) | 14.78 (10.33, 20.62) | 3.15 (2.27, 4.28) | 47.13 (35.81, 61.19) |
| 2013 | 169.7 (145.85, 194.79) | 22.46 (18.96, 27.05) | 14.55 (10.19, 20.31) | 3.1 (2.24, 4.23) | 47.1 (35.75, 61.19) |
| 2014 | 169.49 (145.65, 194.53) | 22.72 (19.2, 27.35) | 14.31 (10.04, 19.99) | 3.06 (2.21, 4.17) | 47.08 (35.69, 61.19) |
| 2015 | 169.23 (145.38, 194.21) | 22.98 (19.41, 27.69) | 14.08 (9.89, 19.68) | 3.03 (2.18, 4.13) | 47.03 (35.63, 61.16) |
| 2016 | 169.43 (145.42, 194.48) | 23.41 (19.78, 28.12) | 13.86 (9.75, 19.36) | 3 (2.16, 4.1) | 47.31 (35.77, 61.49) |
| 2017 | 170.24 (146.05, 195.51) | 24.05 (20.31, 28.83) | 13.64 (9.62, 19.04) | 2.96 (2.13, 4.06) | 47.96 (36.17, 62.45) |
| 2018 | 171.02 (146.85, 196.79) | 24.63 (20.75, 29.46) | 13.43 (9.49, 18.74) | 2.94 (2.11, 4.02) | 48.59 (36.55, 63.46) |
| 2019 | 171.16 (147.07, 197.33) | 24.86 (20.94, 29.7) | 13.25 (9.38, 18.49) | 2.92 (2.1, 4) | 48.81 (36.58, 63.85) |
| 2020 | 169.42 (145.03, 194.68) | 24.35 (20.58, 29.29) | 13.68 (9.65, 19.11) | 2.84 (2.05, 3.89) | 47.06 (35.52, 61.54) |
| 2021 | 169.39 (145.14, 195.01) | 24.54 (20.74, 29.48) | 13.72 (9.7, 19.07) | 2.87 (2.06, 3.93) | 46.77 (35.25, 61.17) |
| **Males** | | | | | |
| 1990 | 197.41 (169.42, 224.95) | 23.52 (19.78, 28.47) | 26.1 (18.23, 36.44) | 3.77 (2.63, 5.3) | 59.97 (45.01, 78.31) |
| 1991 | 198.7 (170.84, 226.21) | 24.28 (20.46, 29.38) | 26.38 (18.5, 36.63) | 3.76 (2.64, 5.28) | 59.9 (45.04, 78.24) |
| 1992 | 199.71 (171.84, 227.35) | 24.97 (21.06, 30.18) | 26.5 (18.65, 36.62) | 3.77 (2.65, 5.28) | 59.83 (45.07, 78.17) |
| 1993 | 200.41 (172.67, 228.16) | 25.56 (21.57, 30.86) | 26.48 (18.65, 36.45) | 3.78 (2.67, 5.29) | 59.76 (45.1, 78.09) |
| 1994 | 200.74 (173.14, 228.35) | 26.06 (22, 31.41) | 26.32 (18.54, 36.09) | 3.79 (2.68, 5.29) | 59.67 (45.14, 77.98) |
| 1995 | 200.64 (173.26, 228.29) | 26.42 (22.31, 31.82) | 25.99 (18.35, 35.58) | 3.8 (2.69, 5.29) | 59.54 (45.13, 77.84) |
| 1996 | 200.29 (173.27, 227.6) | 26.81 (22.64, 32.3) | 25.52 (18.08, 35) | 3.81 (2.71, 5.28) | 59.38 (44.96, 77.43) |
| 1997 | 199.88 (173.11, 226.92) | 27.3 (23.06, 32.92) | 25.01 (17.79, 34.34) | 3.82 (2.72, 5.3) | 59.2 (44.88, 77.08) |
| 1998 | 199.41 (172.9, 226.1) | 27.81 (23.53, 33.55) | 24.5 (17.48, 33.68) | 3.83 (2.74, 5.3) | 59 (44.82, 76.71) |
| 1999 | 198.85 (172.56, 225.41) | 28.2 (23.9, 33.98) | 24.05 (17.2, 33.1) | 3.83 (2.74, 5.28) | 58.8 (44.75, 76.35) |
| 2000 | 198.15 (171.92, 224.66) | 28.35 (24.05, 34.13) | 23.7 (16.98, 32.7) | 3.8 (2.73, 5.23) | 58.61 (44.67, 76.08) |
| 2001 | 197.12 (171.07, 223.49) | 28.26 (23.99, 34.01) | 23.31 (16.68, 32.18) | 3.78 (2.72, 5.17) | 58.46 (44.52, 75.88) |
| 2002 | 195.71 (170.06, 222.03) | 28.02 (23.77, 33.74) | 22.76 (16.25, 31.53) | 3.77 (2.71, 5.11) | 58.32 (44.4, 75.71) |
| 2003 | 194.14 (168.9, 220.44) | 27.68 (23.47, 33.35) | 22.14 (15.75, 30.81) | 3.77 (2.72, 5.08) | 58.2 (44.31, 75.56) |
| 2004 | 192.63 (167.75, 218.93) | 27.28 (23.12, 32.89) | 21.52 (15.25, 30.04) | 3.78 (2.73, 5.07) | 58.11 (44.27, 75.45) |
| 2005 | 191.35 (166.68, 217.67) | 26.88 (22.77, 32.42) | 20.95 (14.8, 29.24) | 3.78 (2.75, 5.07) | 58.05 (44.25, 75.38) |
| 2006 | 190.26 (165.75, 216.36) | 26.33 (22.27, 31.79) | 20.37 (14.36, 28.57) | 3.8 (2.77, 5.1) | 58.11 (44.28, 75.34) |
| 2007 | 189.21 (164.98, 215.12) | 25.59 (21.63, 30.85) | 19.69 (13.82, 27.61) | 3.84 (2.8, 5.15) | 58.32 (44.41, 75.55) |
| 2008 | 188.24 (164.25, 214) | 24.81 (20.97, 29.86) | 19.03 (13.34, 26.63) | 3.88 (2.84, 5.22) | 58.58 (44.57, 75.88) |
| 2009 | 187.43 (163.57, 213.09) | 24.16 (20.42, 29.06) | 18.5 (12.96, 25.89) | 3.92 (2.85, 5.27) | 58.8 (44.7, 76.19) |
| 2010 | 186.78 (162.93, 212.47) | 23.77 (20.13, 28.53) | 18.17 (12.72, 25.44) | 3.93 (2.86, 5.3) | 58.91 (44.75, 76.35) |
| 2011 | 186.29 (162.44, 211.96) | 23.7 (20.08, 28.39) | 17.93 (12.58, 25.11) | 3.91 (2.85, 5.29) | 58.92 (44.71, 76.4) |
| 2012 | 185.93 (162.05, 211.66) | 23.83 (20.18, 28.49) | 17.67 (12.4, 24.75) | 3.88 (2.82, 5.25) | 58.93 (44.69, 76.47) |
| 2013 | 185.66 (161.81, 211.47) | 24.09 (20.39, 28.74) | 17.4 (12.22, 24.38) | 3.83 (2.79, 5.19) | 58.96 (44.68, 76.56) |
| 2014 | 185.41 (161.58, 211.52) | 24.4 (20.63, 29.08) | 17.12 (12.04, 24) | 3.78 (2.75, 5.14) | 58.98 (44.67, 76.64) |
| 2015 | 185.07 (161.3, 211.45) | 24.63 (20.85, 29.41) | 16.87 (11.87, 23.63) | 3.75 (2.72, 5.1) | 58.97 (44.64, 76.68) |
| 2016 | 185.36 (161.26, 212.12) | 25 (21.15, 29.79) | 16.65 (11.75, 23.29) | 3.71 (2.7, 5.05) | 59.45 (44.92, 77.28) |
| 2017 | 186.48 (162.51, 214.36) | 25.57 (21.61, 30.4) | 16.44 (11.64, 22.98) | 3.68 (2.67, 5.01) | 60.51 (45.6, 78.91) |
| 2018 | 187.6 (163.23, 216.15) | 26.07 (22.01, 30.94) | 16.23 (11.53, 22.67) | 3.65 (2.64, 4.98) | 61.55 (46.26, 80.56) |
| 2019 | 187.88 (163.06, 216.49) | 26.21 (22.11, 31.06) | 16.05 (11.42, 22.36) | 3.63 (2.63, 4.96) | 61.97 (46.46, 81.19) |
| 2020 | 183.84 (159.39, 210.76) | 24.91 (21.23, 29.62) | 16.55 (11.72, 23.07) | 3.53 (2.57, 4.82) | 58.54 (44.22, 76.62) |
| 2021 | 183.99 (159.71, 211.51) | 25.2 (21.46, 29.85) | 16.61 (11.77, 23) | 3.55 (2.59, 4.87) | 58.22 (43.62, 76.24) |
| **Females** | | | | | |
| 1990 | 170.75 (143.4, 198.12) | 23.15 (19.27, 28.41) | 19.23 (13.55, 26.89) | 2.4 (1.63, 3.46) | 36.6 (27.5, 47.97) |
| 1991 | 170.13 (142.9, 197.55) | 23.11 (19.24, 28.31) | 19.04 (13.44, 26.58) | 2.38 (1.62, 3.41) | 36.62 (27.61, 47.95) |
| 1992 | 169.48 (142.35, 196.8) | 23.07 (19.22, 28.24) | 18.76 (13.25, 26.19) | 2.37 (1.62, 3.37) | 36.62 (27.72, 47.9) |
| 1993 | 168.82 (141.77, 196.03) | 23.03 (19.2, 28.17) | 18.43 (13.03, 25.72) | 2.36 (1.63, 3.35) | 36.6 (27.8, 47.82) |
| 1994 | 168.13 (141.16, 195.27) | 22.99 (19.18, 28.11) | 18.07 (12.76, 25.21) | 2.37 (1.63, 3.34) | 36.54 (27.85, 47.7) |
| 1995 | 167.41 (140.56, 194.32) | 22.94 (19.16, 28.07) | 17.68 (12.49, 24.67) | 2.37 (1.64, 3.33) | 36.44 (27.85, 47.53) |
| 1996 | 166.71 (140.18, 193.25) | 22.9 (19.16, 28.01) | 17.23 (12.18, 24.04) | 2.38 (1.66, 3.34) | 36.26 (27.68, 47.28) |
| 1997 | 166.11 (139.87, 192.29) | 22.9 (19.18, 28) | 16.72 (11.83, 23.34) | 2.41 (1.68, 3.37) | 36.01 (27.52, 46.89) |
| 1998 | 165.57 (139.61, 191.48) | 22.92 (19.21, 28.02) | 16.2 (11.45, 22.7) | 2.44 (1.71, 3.41) | 35.73 (27.36, 46.46) |
| 1999 | 165.02 (139.34, 190.59) | 22.93 (19.23, 28.04) | 15.74 (11.1, 22.12) | 2.45 (1.72, 3.43) | 35.47 (27.19, 46.06) |
| 2000 | 164.37 (139, 189.69) | 22.92 (19.21, 28.02) | 15.36 (10.82, 21.61) | 2.45 (1.72, 3.41) | 35.28 (27.08, 45.79) |
| 2001 | 163.47 (138.4, 188.57) | 22.86 (19.15, 27.95) | 15.02 (10.56, 21.1) | 2.43 (1.7, 3.37) | 35.14 (26.96, 45.61) |
| 2002 | 162.29 (137.58, 187.22) | 22.76 (19.06, 27.82) | 14.65 (10.28, 20.56) | 2.42 (1.7, 3.33) | 35.01 (26.85, 45.44) |
| 2003 | 161 (136.67, 185.73) | 22.64 (18.96, 27.71) | 14.29 (9.99, 20.07) | 2.41 (1.69, 3.3) | 34.9 (26.75, 45.29) |
| 2004 | 159.76 (135.79, 184.31) | 22.5 (18.84, 27.56) | 13.94 (9.72, 19.63) | 2.4 (1.69, 3.28) | 34.82 (26.68, 45.17) |
| 2005 | 158.7 (135.03, 183.15) | 22.34 (18.69, 27.39) | 13.63 (9.5, 19.18) | 2.4 (1.69, 3.27) | 34.76 (26.62, 45.08) |
| 2006 | 157.75 (134.23, 182.21) | 22.06 (18.45, 27.03) | 13.33 (9.29, 18.74) | 2.41 (1.7, 3.28) | 34.77 (26.62, 45.1) |
| 2007 | 156.74 (133.35, 181.28) | 21.62 (18.08, 26.47) | 13 (9.05, 18.25) | 2.42 (1.71, 3.31) | 34.84 (26.67, 45.22) |
| 2008 | 155.76 (132.48, 180.38) | 21.16 (17.66, 25.86) | 12.67 (8.8, 17.76) | 2.43 (1.72, 3.35) | 34.94 (26.73, 45.36) |
| 2009 | 154.89 (131.72, 179.58) | 20.77 (17.33, 25.36) | 12.38 (8.57, 17.36) | 2.44 (1.72, 3.37) | 35.02 (26.78, 45.49) |
| 2010 | 154.2 (131.13, 179) | 20.58 (17.16, 25.1) | 12.19 (8.44, 17.08) | 2.44 (1.71, 3.38) | 35.03 (26.78, 45.52) |
| 2011 | 153.72 (130.8, 178.52) | 20.56 (17.16, 25.06) | 12.03 (8.32, 16.83) | 2.42 (1.7, 3.34) | 34.98 (26.73, 45.45) |
| 2012 | 153.38 (130.57, 178.18) | 20.62 (17.23, 25.1) | 11.84 (8.22, 16.56) | 2.38 (1.68, 3.28) | 34.91 (26.66, 45.37) |
| 2013 | 153.13 (130.4, 177.96) | 20.75 (17.37, 25.26) | 11.64 (8.1, 16.27) | 2.34 (1.65, 3.22) | 34.83 (26.58, 45.27) |
| 2014 | 152.94 (130.21, 177.56) | 20.96 (17.56, 25.53) | 11.43 (7.97, 15.97) | 2.3 (1.62, 3.17) | 34.75 (26.5, 45.16) |
| 2015 | 152.74 (130.02, 177.15) | 21.23 (17.8, 25.88) | 11.22 (7.85, 15.68) | 2.27 (1.6, 3.13) | 34.66 (26.45, 45.04) |
| 2016 | 152.84 (129.96, 177.11) | 21.72 (18.25, 26.44) | 11 (7.71, 15.35) | 2.25 (1.58, 3.11) | 34.71 (26.47, 45.17) |
| 2017 | 153.3 (130.17, 177.8) | 22.44 (18.85, 27.28) | 10.76 (7.55, 15) | 2.22 (1.55, 3.08) | 34.93 (26.6, 45.54) |
| 2018 | 153.72 (130.34, 178.36) | 23.1 (19.37, 27.97) | 10.54 (7.39, 14.68) | 2.19 (1.53, 3.05) | 35.13 (26.61, 45.91) |
| 2019 | 153.71 (130.12, 178.5) | 23.42 (19.63, 28.26) | 10.36 (7.27, 14.43) | 2.17 (1.51, 3.03) | 35.14 (26.44, 46.05) |
| 2020 | 154.33 (130.64, 179.47) | 23.72 (19.89, 28.91) | 10.72 (7.53, 15) | 2.12 (1.49, 2.96) | 35.14 (26.43, 46.07) |
| 2021 | 154.12 (130.49, 179.18) | 23.82 (19.95, 29.04) | 10.74 (7.55, 14.96) | 2.14 (1.49, 3) | 34.86 (26.17, 45.76) |
