# Supplementary material for: Global burden of disease due to opioid, amphetamine, cocaine, and cannabis use disorders, 1990-2021: a systematic analysis for the Global Burden of Disease Study 2021
Source: PLoS One. 2025 Aug 21;20(8):e0328276. doi: 10.1371/journal.pone.0328276 (PMC12370144; doi:10.1371/journal.pone.0328276)
Supplement: S2 Table — (DOCX) [file pone.0328276.s003.docx]

**S2 Table. Global age-standardized mortality rates (ASMR) per 100,000 attributable to any, opioid, amphetamine, cocaine, and cannabis use disorders, 1990-2021**

| **Year** | **Any drug use disorder, ASMR (95% UI)** | **Opioid use disorder, ASMR (95% UI)** | **Amphetamine use disorder, ASMR (95% UI)** | **Cocaine use disorder, ASMR (95% UI)** |
| --- | --- | --- | --- | --- |
| **Both sexes** | | | | |
| 1990 | 1.26 (1.17, 1.37) | 0.86 (0.76, 0.93) | 0.09 (0.08, 0.11) | 0.07 (0.06, 0.09) |
| 1991 | 1.32 (1.23, 1.43) | 0.9 (0.8, 0.97) | 0.1 (0.08, 0.12) | 0.07 (0.06, 0.09) |
| 1992 | 1.37 (1.28, 1.47) | 0.93 (0.84, 1.01) | 0.1 (0.09, 0.13) | 0.08 (0.07, 0.1) |
| 1993 | 1.41 (1.33, 1.52) | 0.96 (0.88, 1.05) | 0.1 (0.09, 0.12) | 0.08 (0.07, 0.1) |
| 1994 | 1.45 (1.37, 1.52) | 0.99 (0.91, 1.06) | 0.11 (0.09, 0.13) | 0.09 (0.07, 0.11) |
| 1995 | 1.46 (1.38, 1.53) | 1 (0.92, 1.06) | 0.11 (0.1, 0.12) | 0.09 (0.08, 0.11) |
| 1996 | 1.45 (1.36, 1.52) | 0.99 (0.92, 1.05) | 0.11 (0.1, 0.12) | 0.09 (0.08, 0.1) |
| 1997 | 1.42 (1.35, 1.48) | 0.97 (0.91, 1.02) | 0.1 (0.09, 0.12) | 0.09 (0.08, 0.1) |
| 1998 | 1.41 (1.33, 1.47) | 0.96 (0.91, 1.01) | 0.1 (0.09, 0.12) | 0.09 (0.08, 0.11) |
| 1999 | 1.4 (1.34, 1.46) | 0.96 (0.9, 1) | 0.1 (0.09, 0.11) | 0.1 (0.09, 0.11) |
| 2000 | 1.4 (1.34, 1.46) | 0.96 (0.91, 1) | 0.1 (0.09, 0.11) | 0.1 (0.09, 0.12) |
| 2001 | 1.36 (1.31, 1.42) | 0.93 (0.89, 0.97) | 0.1 (0.09, 0.11) | 0.11 (0.09, 0.12) |
| 2002 | 1.32 (1.27, 1.37) | 0.92 (0.88, 0.95) | 0.09 (0.08, 0.1) | 0.11 (0.1, 0.12) |
| 2003 | 1.29 (1.25, 1.33) | 0.9 (0.87, 0.93) | 0.09 (0.08, 0.09) | 0.11 (0.1, 0.12) |
| 2004 | 1.28 (1.25, 1.32) | 0.91 (0.88, 0.94) | 0.09 (0.08, 0.09) | 0.11 (0.1, 0.12) |
| 2005 | 1.33 (1.29, 1.36) | 0.95 (0.92, 0.97) | 0.09 (0.08, 0.09) | 0.11 (0.1, 0.12) |
| 2006 | 1.32 (1.28, 1.36) | 0.95 (0.92, 0.98) | 0.09 (0.08, 0.09) | 0.11 (0.1, 0.12) |
| 2007 | 1.32 (1.28, 1.35) | 0.94 (0.91, 0.97) | 0.09 (0.08, 0.09) | 0.11 (0.1, 0.12) |
| 2008 | 1.31 (1.27, 1.34) | 0.94 (0.91, 0.96) | 0.09 (0.08, 0.09) | 0.11 (0.1, 0.12) |
| 2009 | 1.28 (1.24, 1.31) | 0.91 (0.88, 0.94) | 0.09 (0.08, 0.09) | 0.11 (0.1, 0.12) |
| 2010 | 1.26 (1.23, 1.3) | 0.9 (0.87, 0.93) | 0.09 (0.08, 0.09) | 0.11 (0.1, 0.12) |
| 2011 | 1.26 (1.22, 1.29) | 0.9 (0.87, 0.93) | 0.09 (0.08, 0.09) | 0.11 (0.1, 0.12) |
| 2012 | 1.26 (1.22, 1.29) | 0.9 (0.87, 0.93) | 0.09 (0.08, 0.09) | 0.11 (0.1, 0.12) |
| 2013 | 1.29 (1.25, 1.32) | 0.92 (0.89, 0.95) | 0.09 (0.08, 0.09) | 0.12 (0.11, 0.12) |
| 2014 | 1.33 (1.29, 1.37) | 0.95 (0.92, 0.99) | 0.09 (0.09, 0.09) | 0.12 (0.11, 0.13) |
| 2015 | 1.38 (1.34, 1.42) | 0.99 (0.96, 1.03) | 0.09 (0.09, 0.1) | 0.13 (0.12, 0.14) |
| 2016 | 1.46 (1.42, 1.5) | 1.05 (1.01, 1.08) | 0.1 (0.09, 0.1) | 0.14 (0.13, 0.15) |
| 2017 | 1.52 (1.48, 1.57) | 1.09 (1.06, 1.13) | 0.1 (0.1, 0.11) | 0.15 (0.14, 0.16) |
| 2018 | 1.56 (1.51, 1.61) | 1.12 (1.08, 1.16) | 0.11 (0.1, 0.12) | 0.15 (0.14, 0.16) |
| 2019 | 1.6 (1.54, 1.66) | 1.15 (1.1, 1.2) | 0.11 (0.11, 0.12) | 0.15 (0.14, 0.16) |
| 2020 | 1.61 (1.53, 1.69) | 1.16 (1.1, 1.23) | 0.12 (0.11, 0.13) | 0.15 (0.14, 0.17) |
| 2021 | 1.65 (1.55, 1.75) | 1.19 (1.12, 1.29) | 0.12 (0.11, 0.13) | 0.15 (0.14, 0.17) |
| **Males** | | | | |
| 1990 | 1.74 (1.58, 1.92) | 1.31 (1.17, 1.43) | 0.08 (0.06, 0.11) | 0.09 (0.08, 0.11) |
| 1991 | 1.83 (1.66, 2.02) | 1.37 (1.23, 1.49) | 0.09 (0.07, 0.12) | 0.09 (0.08, 0.12) |
| 1992 | 1.92 (1.76, 2.07) | 1.43 (1.3, 1.55) | 0.1 (0.08, 0.13) | 0.1 (0.09, 0.12) |
| 1993 | 2.02 (1.86, 2.2) | 1.49 (1.37, 1.63) | 0.11 (0.09, 0.14) | 0.11 (0.1, 0.13) |
| 1994 | 2.08 (1.92, 2.21) | 1.53 (1.41, 1.63) | 0.11 (0.09, 0.14) | 0.12 (0.1, 0.14) |
| 1995 | 2.11 (1.98, 2.26) | 1.55 (1.44, 1.65) | 0.12 (0.1, 0.15) | 0.12 (0.1, 0.14) |
| 1996 | 2.11 (1.97, 2.24) | 1.55 (1.44, 1.65) | 0.13 (0.11, 0.15) | 0.12 (0.1, 0.14) |
| 1997 | 2.09 (1.95, 2.21) | 1.52 (1.43, 1.61) | 0.13 (0.11, 0.16) | 0.12 (0.11, 0.14) |
| 1998 | 2.09 (1.96, 2.2) | 1.51 (1.42, 1.6) | 0.13 (0.12, 0.16) | 0.13 (0.11, 0.15) |
| 1999 | 2.09 (1.96, 2.2) | 1.5 (1.41, 1.58) | 0.14 (0.12, 0.16) | 0.14 (0.12, 0.16) |
| 2000 | 2.09 (1.98, 2.19) | 1.49 (1.42, 1.56) | 0.14 (0.12, 0.16) | 0.15 (0.13, 0.16) |
| 2001 | 2.02 (1.92, 2.11) | 1.44 (1.37, 1.5) | 0.13 (0.12, 0.15) | 0.15 (0.13, 0.17) |
| 2002 | 1.94 (1.84, 2.02) | 1.39 (1.33, 1.45) | 0.12 (0.11, 0.14) | 0.15 (0.14, 0.17) |
| 2003 | 1.88 (1.81, 1.95) | 1.36 (1.31, 1.4) | 0.12 (0.11, 0.13) | 0.15 (0.13, 0.17) |
| 2004 | 1.87 (1.8, 1.94) | 1.36 (1.31, 1.4) | 0.12 (0.11, 0.13) | 0.15 (0.14, 0.17) |
| 2005 | 1.95 (1.87, 2.01) | 1.42 (1.37, 1.46) | 0.13 (0.12, 0.14) | 0.16 (0.14, 0.17) |
| 2006 | 1.94 (1.87, 2) | 1.42 (1.37, 1.46) | 0.13 (0.12, 0.14) | 0.16 (0.14, 0.17) |
| 2007 | 1.92 (1.85, 1.98) | 1.4 (1.35, 1.45) | 0.13 (0.12, 0.14) | 0.16 (0.14, 0.17) |
| 2008 | 1.9 (1.83, 1.95) | 1.38 (1.33, 1.42) | 0.13 (0.12, 0.14) | 0.16 (0.14, 0.17) |
| 2009 | 1.83 (1.77, 1.88) | 1.32 (1.28, 1.36) | 0.13 (0.12, 0.13) | 0.15 (0.14, 0.17) |
| 2010 | 1.8 (1.74, 1.85) | 1.3 (1.25, 1.34) | 0.13 (0.12, 0.13) | 0.15 (0.14, 0.17) |
| 2011 | 1.78 (1.72, 1.84) | 1.28 (1.23, 1.33) | 0.13 (0.12, 0.13) | 0.16 (0.15, 0.17) |
| 2012 | 1.78 (1.73, 1.83) | 1.28 (1.23, 1.33) | 0.13 (0.12, 0.13) | 0.16 (0.15, 0.17) |
| 2013 | 1.82 (1.76, 1.88) | 1.31 (1.26, 1.36) | 0.13 (0.12, 0.14) | 0.17 (0.16, 0.18) |
| 2014 | 1.89 (1.82, 1.95) | 1.36 (1.3, 1.41) | 0.13 (0.13, 0.14) | 0.17 (0.16, 0.19) |
| 2015 | 1.96 (1.9, 2.03) | 1.41 (1.36, 1.46) | 0.14 (0.13, 0.15) | 0.19 (0.17, 0.2) |
| 2016 | 2.09 (2.02, 2.15) | 1.5 (1.44, 1.55) | 0.15 (0.14, 0.16) | 0.21 (0.19, 0.22) |
| 2017 | 2.18 (2.11, 2.26) | 1.56 (1.5, 1.62) | 0.16 (0.15, 0.17) | 0.22 (0.2, 0.23) |
| 2018 | 2.24 (2.17, 2.32) | 1.6 (1.55, 1.67) | 0.17 (0.16, 0.18) | 0.22 (0.21, 0.24) |
| 2019 | 2.31 (2.22, 2.4) | 1.65 (1.58, 1.72) | 0.18 (0.16, 0.19) | 0.23 (0.21, 0.25) |
| 2020 | 2.31 (2.2, 2.44) | 1.66 (1.58, 1.76) | 0.18 (0.16, 0.2) | 0.22 (0.2, 0.26) |
| 2021 | 2.37 (2.24, 2.51) | 1.71 (1.61, 1.84) | 0.19 (0.17, 0.21) | 0.22 (0.2, 0.27) |
| **Females** | | | | |
| 1990 | 0.79 (0.7, 0.89) | 0.42 (0.36, 0.49) | 0.1 (0.08, 0.13) | 0.05 (0.04, 0.07) |
| 1991 | 0.81 (0.73, 0.91) | 0.44 (0.37, 0.51) | 0.11 (0.09, 0.13) | 0.05 (0.04, 0.07) |
| 1992 | 0.82 (0.74, 0.91) | 0.45 (0.39, 0.51) | 0.11 (0.09, 0.13) | 0.05 (0.04, 0.07) |
| 1993 | 0.82 (0.75, 0.9) | 0.45 (0.38, 0.51) | 0.1 (0.08, 0.12) | 0.05 (0.04, 0.07) |
| 1994 | 0.83 (0.76, 0.9) | 0.46 (0.4, 0.51) | 0.1 (0.08, 0.12) | 0.06 (0.04, 0.07) |
| 1995 | 0.81 (0.75, 0.88) | 0.45 (0.4, 0.51) | 0.1 (0.08, 0.11) | 0.06 (0.04, 0.07) |
| 1996 | 0.79 (0.74, 0.85) | 0.45 (0.4, 0.49) | 0.09 (0.08, 0.1) | 0.06 (0.05, 0.07) |
| 1997 | 0.76 (0.71, 0.81) | 0.43 (0.39, 0.48) | 0.08 (0.07, 0.09) | 0.06 (0.05, 0.07) |
| 1998 | 0.74 (0.7, 0.79) | 0.43 (0.39, 0.46) | 0.08 (0.07, 0.09) | 0.06 (0.05, 0.07) |
| 1999 | 0.73 (0.69, 0.77) | 0.43 (0.4, 0.46) | 0.07 (0.06, 0.08) | 0.06 (0.05, 0.07) |
| 2000 | 0.73 (0.69, 0.77) | 0.44 (0.4, 0.47) | 0.07 (0.06, 0.08) | 0.06 (0.05, 0.08) |
| 2001 | 0.72 (0.69, 0.76) | 0.44 (0.41, 0.47) | 0.06 (0.06, 0.07) | 0.06 (0.05, 0.08) |
| 2002 | 0.72 (0.69, 0.75) | 0.45 (0.42, 0.48) | 0.06 (0.05, 0.07) | 0.06 (0.05, 0.08) |
| 2003 | 0.71 (0.68, 0.73) | 0.46 (0.43, 0.48) | 0.05 (0.05, 0.06) | 0.06 (0.05, 0.08) |
| 2004 | 0.7 (0.68, 0.73) | 0.47 (0.44, 0.49) | 0.05 (0.05, 0.06) | 0.06 (0.05, 0.08) |
| 2005 | 0.72 (0.69, 0.74) | 0.48 (0.46, 0.5) | 0.05 (0.05, 0.06) | 0.06 (0.05, 0.08) |
| 2006 | 0.71 (0.69, 0.74) | 0.49 (0.47, 0.51) | 0.05 (0.05, 0.05) | 0.06 (0.06, 0.07) |
| 2007 | 0.72 (0.69, 0.74) | 0.49 (0.47, 0.51) | 0.05 (0.05, 0.05) | 0.06 (0.06, 0.07) |
| 2008 | 0.72 (0.7, 0.75) | 0.5 (0.48, 0.52) | 0.05 (0.05, 0.05) | 0.06 (0.06, 0.07) |
| 2009 | 0.73 (0.71, 0.75) | 0.5 (0.49, 0.52) | 0.05 (0.05, 0.05) | 0.06 (0.06, 0.07) |
| 2010 | 0.73 (0.71, 0.76) | 0.51 (0.49, 0.53) | 0.05 (0.05, 0.05) | 0.06 (0.06, 0.07) |
| 2011 | 0.74 (0.72, 0.76) | 0.52 (0.5, 0.53) | 0.05 (0.04, 0.05) | 0.06 (0.06, 0.07) |
| 2012 | 0.74 (0.72, 0.76) | 0.52 (0.5, 0.54) | 0.05 (0.04, 0.05) | 0.06 (0.06, 0.07) |
| 2013 | 0.76 (0.73, 0.78) | 0.53 (0.51, 0.55) | 0.05 (0.04, 0.05) | 0.07 (0.06, 0.07) |
| 2014 | 0.78 (0.75, 0.8) | 0.55 (0.53, 0.58) | 0.05 (0.04, 0.05) | 0.07 (0.06, 0.08) |
| 2015 | 0.8 (0.77, 0.83) | 0.57 (0.55, 0.6) | 0.05 (0.05, 0.05) | 0.07 (0.07, 0.08) |
| 2016 | 0.84 (0.81, 0.87) | 0.6 (0.58, 0.62) | 0.05 (0.05, 0.05) | 0.07 (0.07, 0.08) |
| 2017 | 0.87 (0.84, 0.9) | 0.63 (0.6, 0.65) | 0.05 (0.05, 0.05) | 0.08 (0.07, 0.08) |
| 2018 | 0.88 (0.84, 0.92) | 0.64 (0.61, 0.67) | 0.05 (0.05, 0.06) | 0.08 (0.07, 0.08) |
| 2019 | 0.9 (0.85, 0.94) | 0.65 (0.61, 0.69) | 0.05 (0.05, 0.06) | 0.08 (0.07, 0.09) |
| 2020 | 0.91 (0.85, 0.97) | 0.66 (0.61, 0.72) | 0.05 (0.05, 0.06) | 0.08 (0.07, 0.09) |
| 2021 | 0.93 (0.85, 1.01) | 0.68 (0.61, 0.76) | 0.05 (0.05, 0.06) | 0.08 (0.07, 0.09) |
