# Supplementary material for: Global burden of disease due to opioid, amphetamine, cocaine, and cannabis use disorders, 1990-2021: a systematic analysis for the Global Burden of Disease Study 2021
Source: PLoS One. 2025 Aug 21;20(8):e0328276. doi: 10.1371/journal.pone.0328276 (PMC12370144; doi:10.1371/journal.pone.0328276)
Supplement: S3 Table — (DOCX) [file pone.0328276.s004.docx]

**S3 Table. Global age-standardized disability-adjusted life year (DALY) rates per 100,000 attributable to any, opioid, amphetamine, cocaine, and cannabis use disorders, 1990-2021**

| **Year** | **Any drug use disorder, DALY rate (95% UI)** | **Opioid use disorder, DALY rate (95% UI)** | **Amphetamine use disorder, DALY rate (95% UI)** | **Cocaine use disorder, DALY rate (95% UI)** | **Cannabis use disorder, DALY rate (95% UI)** |
| --- | --- | --- | --- | --- | --- |
| **Both sexes** | | | | | |
| 1990 | 166.44 (132.55, 198.4) | 103.69 (81.83, 122.75) | 29.63 (19.51, 43.52) | 10.91 (7.86, 14.84) | 8.63 (5.1, 13.25) |
| 1991 | 170.48 (135.78, 201.15) | 106.56 (84.3, 126.2) | 30.27 (20.09, 44.36) | 10.94 (7.95, 14.81) | 8.62 (5.08, 13.22) |
| 1992 | 173.96 (140.12, 205.31) | 109.15 (85.93, 128.97) | 30.68 (20.31, 44.61) | 11.05 (8.06, 14.8) | 8.61 (5.08, 13.17) |
| 1993 | 176.93 (143.78, 210.3) | 111.51 (88.67, 131.31) | 30.82 (20.38, 44.87) | 11.29 (8.31, 15.11) | 8.59 (5.06, 13.17) |
| 1994 | 179.12 (144.83, 211.52) | 113.3 (89.08, 133.34) | 30.83 (20.41, 45) | 11.48 (8.53, 15.3) | 8.57 (5.05, 13.13) |
| 1995 | 179.7 (146.34, 212.84) | 114.15 (91.53, 134.78) | 30.6 (20.4, 44.37) | 11.5 (8.55, 15.31) | 8.55 (5.05, 13.12) |
| 1996 | 179.09 (144.4, 210.57) | 114.22 (91.15, 134.43) | 30.15 (20.19, 43.83) | 11.48 (8.53, 15.27) | 8.52 (5.07, 13.06) |
| 1997 | 177.73 (144.47, 209.57) | 113.91 (90.08, 134.03) | 29.45 (19.64, 43.02) | 11.55 (8.6, 15.31) | 8.49 (5.04, 13.05) |
| 1998 | 177.47 (143.8, 209.05) | 114.29 (91.25, 135.22) | 28.78 (19.38, 41.71) | 11.75 (8.85, 15.56) | 8.44 (5.02, 13.03) |
| 1999 | 177.47 (145.42, 209.44) | 114.84 (91.07, 135.87) | 28.09 (18.85, 40.81) | 12.09 (9.14, 15.92) | 8.4 (5.02, 12.95) |
| 2000 | 177.22 (145.13, 208.61) | 115.14 (91.77, 135.82) | 27.51 (18.21, 39.88) | 12.35 (9.39, 16.18) | 8.36 (4.98, 12.9) |
| 2001 | 174.71 (143.24, 207.12) | 114.08 (91.26, 135.06) | 26.69 (17.75, 38.62) | 12.46 (9.55, 16.33) | 8.34 (4.97, 12.89) |
| 2002 | 171.93 (139.55, 203.89) | 113.09 (89.58, 134.29) | 25.72 (17.2, 37.32) | 12.49 (9.61, 16.27) | 8.31 (4.93, 12.86) |
| 2003 | 169.5 (137.39, 200.87) | 112.51 (89.59, 133.83) | 24.78 (16.44, 35.88) | 12.47 (9.56, 16.42) | 8.29 (4.94, 12.86) |
| 2004 | 168.61 (137.1, 199.69) | 112.79 (89.74, 133.93) | 24.07 (15.98, 34.65) | 12.5 (9.64, 16.3) | 8.27 (4.94, 12.78) |
| 2005 | 170.18 (139.1, 200.73) | 114.65 (91.81, 136.16) | 23.59 (15.67, 33.91) | 12.71 (9.86, 16.48) | 8.24 (4.9, 12.77) |
| 2006 | 168.87 (137.6, 199.2) | 114.17 (91.51, 135.29) | 23 (15.33, 32.99) | 12.73 (9.86, 16.43) | 8.24 (4.91, 12.79) |
| 2007 | 166.55 (135.86, 196.01) | 112.71 (90.7, 133.34) | 22.33 (14.93, 32.17) | 12.65 (9.85, 16.33) | 8.25 (4.9, 12.73) |
| 2008 | 164.27 (134.26, 193.34) | 111.15 (89.45, 131.77) | 21.71 (14.53, 31.26) | 12.59 (9.77, 16.28) | 8.28 (4.92, 12.78) |
| 2009 | 161.04 (131.56, 189.66) | 108.88 (87.99, 128.88) | 21.15 (14.08, 30.42) | 12.45 (9.66, 16.1) | 8.29 (4.93, 12.85) |
| 2010 | 159.72 (130.53, 188.59) | 108.02 (87.08, 128.05) | 20.86 (13.86, 30.03) | 12.4 (9.68, 16.02) | 8.29 (4.9, 12.87) |
| 2011 | 159.6 (130.41, 187.74) | 108.21 (87.32, 128.28) | 20.68 (13.83, 29.67) | 12.44 (9.72, 16.02) | 8.29 (4.9, 12.84) |
| 2012 | 160.23 (130.67, 188.7) | 109.2 (87.87, 129.31) | 20.47 (13.62, 29.42) | 12.43 (9.75, 15.96) | 8.29 (4.92, 12.82) |
| 2013 | 162.53 (132.37, 191.56) | 111.48 (89.76, 132.11) | 20.34 (13.54, 29.11) | 12.55 (9.88, 16.03) | 8.29 (4.93, 12.85) |
| 2014 | 165.77 (134.95, 194.56) | 114.53 (92.2, 135.68) | 20.27 (13.5, 29.07) | 12.72 (10.09, 16.11) | 8.28 (4.93, 12.87) |
| 2015 | 169.39 (138.17, 198.69) | 117.85 (95.59, 139.37) | 20.21 (13.53, 28.94) | 13 (10.36, 16.42) | 8.28 (4.92, 12.91) |
| 2016 | 174.96 (143.57, 204.46) | 122.52 (99.69, 144.7) | 20.27 (13.72, 28.78) | 13.53 (10.87, 16.99) | 8.34 (4.95, 12.97) |
| 2017 | 180.21 (148.44, 210.88) | 127.14 (103.81, 150.2) | 20.28 (13.87, 28.62) | 13.84 (11.15, 17.32) | 8.48 (5.01, 13.19) |
| 2018 | 183.89 (151.01, 215.16) | 130.51 (106.42, 154.26) | 20.27 (13.95, 28.58) | 13.9 (11.23, 17.32) | 8.62 (5.11, 13.44) |
| 2019 | 187.14 (152.98, 218.91) | 133.47 (108.93, 157.41) | 20.24 (14.07, 28.47) | 14.02 (11.36, 17.45) | 8.68 (5.12, 13.51) |
| 2020 | 189.97 (154.54, 222.08) | 136.33 (110.98, 160.4) | 20.86 (14.41, 29.26) | 13.87 (11.23, 17.37) | 8.32 (4.93, 12.95) |
| 2021 | 190.97 (156.11, 222.79) | 137.15 (112.29, 161.39) | 20.98 (14.56, 29.33) | 13.88 (11.18, 17.52) | 8.27 (4.9, 12.86) |
| **Males** | | | | | |
| 1990 | 198.52 (161.3, 234.34) | 125.14 (102.6, 144.69) | 32.94 (20.96, 48.96) | 14.11 (10.23, 19.24) | 10.89 (6.39, 16.69) |
| 1991 | 205.49 (167.55, 239.83) | 130.27 (106.74, 149.9) | 34.01 (21.81, 50.65) | 14.21 (10.38, 19.25) | 10.86 (6.39, 16.68) |
| 1992 | 212.22 (173.59, 247.04) | 135.15 (110.13, 156.03) | 34.95 (22.53, 51.5) | 14.44 (10.67, 19.43) | 10.85 (6.39, 16.63) |
| 1993 | 218.87 (182.3, 255.88) | 139.85 (115.37, 160.89) | 35.68 (23.2, 52.53) | 14.91 (11.13, 19.86) | 10.83 (6.37, 16.62) |
| 1994 | 223.28 (183.36, 258.54) | 143.21 (117.35, 165.25) | 36.02 (23.46, 52.86) | 15.24 (11.49, 20.37) | 10.8 (6.33, 16.54) |
| 1995 | 225.97 (188.62, 264.17) | 145.47 (121.3, 166.63) | 36.15 (23.8, 52.98) | 15.3 (11.54, 20.44) | 10.78 (6.33, 16.61) |
| 1996 | 226.63 (187.62, 263.11) | 146.33 (122.42, 168.8) | 36.02 (23.92, 52.66) | 15.24 (11.5, 20.23) | 10.76 (6.36, 16.49) |
| 1997 | 226.2 (188.12, 264.35) | 146.49 (120.9, 169.08) | 35.61 (23.61, 51.89) | 15.3 (11.59, 20.18) | 10.73 (6.35, 16.53) |
| 1998 | 227.39 (188.24, 264.97) | 147.74 (122.38, 171.79) | 35.19 (23.62, 50.7) | 15.59 (11.89, 20.57) | 10.69 (6.36, 16.51) |
| 1999 | 228.56 (191.91, 266.47) | 148.9 (122.52, 171.94) | 34.7 (23.47, 49.71) | 16.13 (12.43, 21.03) | 10.67 (6.36, 16.45) |
| 2000 | 228.92 (191.27, 265.85) | 149.38 (124.4, 172.21) | 34.27 (22.99, 49.27) | 16.56 (12.84, 21.33) | 10.63 (6.31, 16.44) |
| 2001 | 225.11 (188.25, 263.04) | 147.33 (122.3, 171.46) | 33.39 (22.49, 48.19) | 16.71 (13.07, 21.52) | 10.6 (6.31, 16.4) |
| 2002 | 220.59 (183.58, 258.67) | 145.16 (119.19, 170.23) | 32.24 (21.71, 46.29) | 16.76 (13.12, 21.4) | 10.57 (6.28, 16.33) |
| 2003 | 217.22 (180.64, 254.41) | 143.97 (118.71, 168.11) | 31.21 (20.94, 44.78) | 16.77 (13.11, 21.63) | 10.55 (6.29, 16.28) |
| 2004 | 216.4 (180.1, 253.22) | 144.41 (119, 168.37) | 30.44 (20.46, 43.31) | 16.83 (13.12, 21.58) | 10.52 (6.26, 16.32) |
| 2005 | 219.59 (183.95, 256.56) | 147.54 (122.1, 171.99) | 29.98 (20.28, 42.79) | 17.2 (13.54, 22.04) | 10.5 (6.2, 16.29) |
| 2006 | 218.17 (181.82, 254.5) | 146.99 (122.09, 170.57) | 29.27 (19.8, 41.35) | 17.29 (13.57, 22.09) | 10.5 (6.24, 16.32) |
| 2007 | 214.8 (179.37, 249.94) | 144.75 (120.53, 167.93) | 28.37 (19.28, 40.21) | 17.19 (13.5, 21.93) | 10.52 (6.2, 16.25) |
| 2008 | 211.16 (176.71, 245.57) | 142.11 (118, 165.05) | 27.52 (18.82, 38.76) | 17.13 (13.52, 21.87) | 10.57 (6.23, 16.38) |
| 2009 | 205.4 (172.33, 239.95) | 137.89 (114.79, 160.42) | 26.7 (18.14, 37.87) | 16.88 (13.23, 21.61) | 10.6 (6.26, 16.37) |
| 2010 | 202.65 (168.92, 237.44) | 135.82 (112.95, 158.35) | 26.32 (17.86, 37.33) | 16.81 (13.15, 21.57) | 10.6 (6.21, 16.44) |
| 2011 | 201.62 (168.39, 235.28) | 135.16 (112.27, 157.83) | 26.1 (17.81, 36.84) | 16.89 (13.29, 21.58) | 10.6 (6.24, 16.36) |
| 2012 | 201.84 (168.28, 235.89) | 135.7 (112.25, 158.48) | 25.88 (17.59, 36.51) | 16.94 (13.4, 21.53) | 10.6 (6.27, 16.42) |
| 2013 | 204.35 (169.92, 238.9) | 138.04 (114.5, 161.1) | 25.76 (17.6, 36.47) | 17.16 (13.61, 21.74) | 10.61 (6.29, 16.48) |
| 2014 | 208.23 (173.51, 242.7) | 141.41 (117.79, 165.02) | 25.76 (17.57, 36.45) | 17.47 (13.93, 22.05) | 10.61 (6.27, 16.52) |
| 2015 | 212.45 (177.03, 247.12) | 144.98 (121.41, 168.63) | 25.79 (17.66, 36.43) | 17.95 (14.36, 22.44) | 10.61 (6.28, 16.51) |
| 2016 | 219.31 (183.53, 252.95) | 150.15 (126.62, 174.62) | 26.05 (18.22, 36.51) | 18.88 (15.34, 23.3) | 10.71 (6.33, 16.74) |
| 2017 | 224.97 (189.66, 258.78) | 154.62 (130.6, 179.02) | 26.26 (18.53, 36.46) | 19.41 (15.85, 23.94) | 10.94 (6.41, 17.08) |
| 2018 | 228.54 (191.39, 262.21) | 157.44 (133.08, 182.12) | 26.47 (18.83, 36.4) | 19.51 (16, 23.96) | 11.16 (6.57, 17.41) |
| 2019 | 232.35 (194.59, 266.46) | 160.56 (135.77, 185.08) | 26.61 (19.06, 36.51) | 19.74 (16.25, 24.22) | 11.26 (6.6, 17.61) |
| 2020 | 234.19 (195.82, 269.68) | 162.97 (137.25, 187.57) | 27.31 (19.42, 37.53) | 19.4 (15.87, 24.23) | 10.57 (6.24, 16.43) |
| 2021 | 235.88 (199.5, 270.25) | 164.33 (138.97, 188.54) | 27.58 (19.78, 37.7) | 19.36 (15.7, 24.42) | 10.51 (6.2, 16.42) |
| **Females** | | | | | |
| 1990 | 133.73 (102.8, 161.92) | 81.86 (60.1, 101.53) | 26.27 (17.56, 37.68) | 7.65 (5.5, 10.81) | 6.33 (3.78, 9.75) |
| 1991 | 134.76 (105.83, 162.79) | 82.44 (61.32, 102.74) | 26.46 (17.97, 37.97) | 7.6 (5.48, 10.67) | 6.32 (3.76, 9.74) |
| 1992 | 134.93 (104.92, 164.18) | 82.68 (60.56, 102.49) | 26.35 (17.66, 37.93) | 7.59 (5.45, 10.59) | 6.32 (3.76, 9.68) |
| 1993 | 134.16 (104.53, 162.53) | 82.67 (61.48, 102.8) | 25.89 (17.35, 37.23) | 7.61 (5.52, 10.6) | 6.31 (3.78, 9.73) |
| 1994 | 134.13 (105.14, 161.7) | 82.88 (61.51, 101.8) | 25.57 (17.16, 37.08) | 7.64 (5.59, 10.66) | 6.3 (3.73, 9.73) |
| 1995 | 132.63 (102.9, 160.59) | 82.33 (60.42, 101.65) | 24.97 (16.69, 35.75) | 7.64 (5.6, 10.62) | 6.28 (3.73, 9.64) |
| 1996 | 130.8 (101.67, 158.25) | 81.64 (60.51, 100.52) | 24.22 (16.05, 34.82) | 7.66 (5.6, 10.59) | 6.25 (3.71, 9.63) |
| 1997 | 128.56 (99.34, 156.25) | 80.88 (59.89, 100.07) | 23.23 (15.41, 33.69) | 7.73 (5.67, 10.72) | 6.2 (3.72, 9.55) |
| 1998 | 126.89 (98.98, 153.38) | 80.43 (59.68, 99.16) | 22.32 (14.75, 32.49) | 7.85 (5.77, 10.74) | 6.15 (3.68, 9.48) |
| 1999 | 125.75 (97.91, 153.03) | 80.38 (59.47, 99.47) | 21.44 (14.11, 31.33) | 8.01 (5.96, 10.9) | 6.1 (3.66, 9.4) |
| 2000 | 124.94 (97.99, 150.73) | 80.52 (60.11, 99.44) | 20.72 (13.73, 30.14) | 8.1 (6.02, 11) | 6.06 (3.64, 9.3) |
| 2001 | 123.78 (96.27, 149.64) | 80.48 (59.84, 99.36) | 19.97 (13.08, 29.22) | 8.16 (6.09, 11.09) | 6.03 (3.59, 9.33) |
| 2002 | 122.76 (95.95, 148.53) | 80.67 (60.34, 99.85) | 19.17 (12.55, 28.17) | 8.18 (6.09, 11.06) | 6.01 (3.59, 9.22) |
| 2003 | 121.28 (94.14, 146.84) | 80.71 (60.19, 99.55) | 18.33 (11.93, 26.92) | 8.14 (6.07, 11.06) | 5.99 (3.59, 9.3) |
| 2004 | 120.34 (93.68, 145.34) | 80.84 (60.36, 99.38) | 17.66 (11.37, 26.08) | 8.13 (6.06, 10.96) | 5.97 (3.59, 9.22) |
| 2005 | 120.27 (94.16, 145.58) | 81.41 (61.13, 100.07) | 17.18 (11.09, 25.35) | 8.19 (6.09, 10.96) | 5.95 (3.59, 9.18) |
| 2006 | 119.09 (93.02, 143.76) | 80.99 (60.79, 99.59) | 16.7 (10.76, 24.79) | 8.13 (6.07, 10.84) | 5.94 (3.57, 9.18) |
| 2007 | 117.81 (92.14, 142.15) | 80.32 (60.58, 98.51) | 16.25 (10.41, 24.13) | 8.07 (6.06, 10.76) | 5.94 (3.56, 9.17) |
| 2008 | 116.87 (91.55, 140.79) | 79.83 (60.42, 97.71) | 15.87 (10.14, 23.64) | 8.01 (6.01, 10.66) | 5.95 (3.56, 9.14) |
| 2009 | 116.18 (91.32, 139.37) | 79.5 (60.46, 97.01) | 15.56 (9.98, 23.04) | 7.99 (6, 10.57) | 5.95 (3.54, 9.23) |
| 2010 | 116.3 (91.17, 140.17) | 79.86 (60.68, 97.55) | 15.37 (9.9, 22.74) | 7.97 (6, 10.54) | 5.94 (3.56, 9.22) |
| 2011 | 117.1 (92.02, 140.59) | 80.92 (61.73, 98.5) | 15.23 (9.9, 22.5) | 7.96 (6.01, 10.56) | 5.94 (3.53, 9.22) |
| 2012 | 118.14 (92.66, 142.68) | 82.35 (62.83, 100.34) | 15.04 (9.64, 22.14) | 7.9 (5.98, 10.48) | 5.93 (3.56, 9.19) |
| 2013 | 120.2 (94.52, 144.69) | 84.56 (64.31, 103.07) | 14.88 (9.63, 21.93) | 7.92 (6.09, 10.42) | 5.92 (3.55, 9.19) |
| 2014 | 122.77 (96.47, 147.97) | 87.27 (66.34, 106.28) | 14.73 (9.49, 21.66) | 7.94 (6.14, 10.43) | 5.91 (3.55, 9.13) |
| 2015 | 125.75 (98.82, 151.33) | 90.33 (68.96, 109.97) | 14.58 (9.4, 21.42) | 8.01 (6.21, 10.45) | 5.91 (3.53, 9.15) |
| 2016 | 129.99 (102.3, 156.17) | 94.46 (72.42, 114.92) | 14.44 (9.35, 21.17) | 8.15 (6.36, 10.59) | 5.92 (3.54, 9.16) |
| 2017 | 134.8 (106.32, 162.04) | 99.22 (76.36, 120.59) | 14.24 (9.27, 20.88) | 8.23 (6.43, 10.66) | 5.98 (3.57, 9.21) |
| 2018 | 138.56 (109.85, 166.58) | 103.12 (79.32, 125.69) | 14.01 (9.18, 20.59) | 8.24 (6.39, 10.64) | 6.03 (3.63, 9.3) |
| 2019 | 141.2 (111.11, 169.66) | 105.9 (81.33, 129.66) | 13.79 (9.06, 20.08) | 8.25 (6.44, 10.7) | 6.03 (3.59, 9.32) |
| 2020 | 144.97 (113.33, 175.13) | 109.18 (83.39, 133.16) | 14.32 (9.37, 20.84) | 8.29 (6.48, 10.84) | 6.02 (3.58, 9.32) |
| 2021 | 145.25 (113.42, 175.63) | 109.42 (84.49, 133.54) | 14.28 (9.36, 20.94) | 8.34 (6.55, 10.97) | 5.97 (3.54, 9.21) |
