# Supplementary material for: Global burden of disease due to opioid, amphetamine, cocaine, and cannabis use disorders, 1990-2021: a systematic analysis for the Global Burden of Disease Study 2021
Source: PLoS One. 2025 Aug 21;20(8):e0328276. doi: 10.1371/journal.pone.0328276 (PMC12370144; doi:10.1371/journal.pone.0328276)
Supplement: S4 Table — (DOCX) [file pone.0328276.s005.docx]

**S4 Table. Age-standardized incidence rates (ASIRs) per 100,000 attributable to any, opioid, amphetamine, cocaine, and cannabis use disorders, stratified by world region, 1990-2021**

| **World region** | **Any drug use disorder, ASIR (95% UI)** | **Opioid use disorder, ASIR (95% UI)** | **Amphetamine use disorder, ASIR (95% UI)** | **Cocaine use disorder, ASIR (95% UI)** | **Cannabis use disorder, ASIR (95% UI)** |
| --- | --- | --- | --- | --- | --- |
| Global | 169.39 (145.14, 195.01) | 24.54 (20.74, 29.48) | 13.72 (9.7, 19.07) | 2.87 (2.06, 3.93) | 46.77 (35.25, 61.17) |
| Low SDI | 110.82 (92.59, 128.79) | 14.35 (11.93, 17.42) | 4.17 (2.85, 5.84) | 0.63 (0.45, 0.89) | 35.15 (25.65, 47.64) |
| Low-middle SDI | 130.55 (110.96, 151.41) | 18.22 (15.29, 22.17) | 5.28 (3.66, 7.46) | 1.1 (0.75, 1.49) | 38.86 (29.16, 51.76) |
| Middle SDI | 155.19 (131.25, 179.27) | 18.82 (15.81, 22.76) | 16.65 (11.67, 23.46) | 2.33 (1.59, 3.33) | 42.89 (31.99, 56.76) |
| High-middle SDI | 189.65 (161.53, 218.09) | 27.16 (23.02, 32.63) | 22.78 (15.75, 32.04) | 3.29 (2.23, 4.73) | 44.92 (34.37, 57.91) |
| High SDI | 350.9 (307.36, 400.2) | 68.52 (57.67, 82.33) | 30.22 (21.39, 41.49) | 13.52 (10.02, 18.91) | 106.41 (80.4, 136.64) |
| Andean Latin America | 147.25 (123.64, 171.11) | 17.39 (13.8, 21.67) | 9.29 (6.33, 13.13) | 5.27 (3.32, 8.08) | 39.76 (29.1, 52.9) |
| Australasia | 425.48 (369.38, 483.04) | 44.87 (38.68, 51.99) | 55.68 (38.08, 78.43) | 12.43 (8.34, 18.59) | 114.65 (90.13, 144.62) |
| Caribbean | 180.09 (147.21, 220.4) | 15.61 (12.48, 19.26) | 6.95 (4.79, 9.85) | 7.82 (4.84, 11.94) | 74.97 (49.43, 108.44) |
| Central Asia | 169.72 (143.57, 197.09) | 36.68 (30.96, 43.7) | 16.61 (11.63, 23.13) | 2.1 (1.42, 2.99) | 33.79 (22.62, 50.51) |
| Central Europe | 184.24 (155.27, 214.63) | 16.1 (13.62, 18.96) | 23.83 (16.19, 33.54) | 3.08 (2, 4.57) | 55.12 (42.33, 71.28) |
| Central Latin America | 144.04 (121.38, 167.38) | 15.25 (12.19, 18.85) | 6.75 (4.59, 9.38) | 7.56 (4.83, 11.58) | 42.34 (32.65, 54.46) |
| East Asia | 173.93 (146.09, 204.63) | 16.71 (13.88, 20.27) | 32.31 (22.4, 45.25) | 0.72 (0.46, 1.05) | 36.16 (26.09, 49.11) |
| Eastern Europe | 275.72 (238.8, 312.9) | 73.32 (61.9, 87.26) | 26.25 (18.74, 35.39) | 4.35 (3.13, 6.05) | 55.89 (37.85, 79.13) |
| Eastern Sub-Saharan Africa | 101.09 (83.77, 119.6) | 10.77 (8.94, 12.99) | 4.98 (3.36, 7) | 0.48 (0.34, 0.68) | 36.35 (25.69, 50.62) |
| High-income Asia Pacific | 204.38 (168.19, 247.27) | 14.92 (12.05, 18.42) | 15.15 (10.11, 21.23) | 6.5 (4.43, 9.5) | 78.59 (55.32, 109.81) |
| High-income North America | 520.07 (454.13, 592.82) | 144.24 (120.13, 174.95) | 42 (29.89, 56.72) | 23.87 (17.69, 33.93) | 151.3 (114.29, 196.4) |
| North Africa and Middle East | 143.52 (120.87, 169.07) | 37.82 (31.5, 45.62) | 4.75 (3.3, 6.67) | 1.62 (1.07, 2.34) | 22.73 (16.1, 31.39) |
| Oceania | 173.25 (141.6, 212) | 12.49 (10.2, 15.23) | 17.24 (11.45, 25) | 0.28 (0.16, 0.42) | 72.23 (47.14, 102.46) |
| South Asia | 131.41 (109.78, 153.28) | 18.9 (15.73, 23.12) | 1.73 (1.21, 2.43) | 0.37 (0.24, 0.54) | 44.47 (32.43, 59.54) |
| Southeast Asia | 141.48 (116.93, 166.01) | 9.36 (7.79, 11.23) | 22.65 (15.16, 32.8) | 0.24 (0.14, 0.36) | 46.3 (33.74, 63.67) |
| Southern Latin America | 196.13 (167.54, 227.34) | 17.75 (14.11, 22.1) | 10.73 (7.23, 15.12) | 14.82 (9.74, 22.82) | 59.35 (48.47, 72.21) |
| Southern Sub-Saharan Africa | 161.51 (137.31, 186.47) | 23.31 (19.68, 28.1) | 11.74 (8.21, 16.33) | 5.51 (3.89, 7.67) | 46.14 (32.43, 63.24) |
| Tropical Latin America | 180.4 (153.43, 207.8) | 15.82 (12.43, 19.74) | 19.4 (12.91, 27.72) | 12.04 (8.21, 18.04) | 64.81 (48.52, 84.57) |
| Western Europe | 302 (262.87, 348.16) | 24.07 (20.69, 28.09) | 25.31 (17.31, 35.24) | 9.53 (6.2, 14.54) | 96.2 (76.54, 119.49) |
| Western Sub-Saharan Africa | 94.68 (79.6, 111.36) | 10.88 (8.95, 13.31) | 4.66 (3.16, 6.6) | 0.7 (0.53, 0.9) | 21.72 (15.81, 29.29) |
