# Supplementary material for: Global burden of disease due to opioid, amphetamine, cocaine, and cannabis use disorders, 1990-2021: a systematic analysis for the Global Burden of Disease Study 2021
Source: PLoS One. 2025 Aug 21;20(8):e0328276. doi: 10.1371/journal.pone.0328276 (PMC12370144; doi:10.1371/journal.pone.0328276)
Supplement: S5 Table — (DOCX) [file pone.0328276.s006.docx]

**S5 Table. Age-standardized mortality rates (ASMRs) per 100,000 attributable to any, opioid, amphetamine, cocaine, and cannabis use disorders, stratified by world region, 1990-2021**

| **World region** | **Any drug use disorder, ASMR (95% UI)** | **Opioid use disorder, ASMR (95% UI)** | **Amphetamine use disorder, ASMR (95% UI)** | **Cocaine use disorder, ASMR (95% UI)** |
| --- | --- | --- | --- | --- |
| Global | 1.65 (1.55, 1.75) | 1.19 (1.12, 1.29) | 0.12 (0.11, 0.13) | 0.15 (0.14, 0.17) |
| Low SDI | 0.51 (0.4, 0.62) | 0.43 (0.34, 0.51) | 0.01 (0.01, 0.01) | 0.04 (0.02, 0.06) |
| Low-middle SDI | 0.59 (0.53, 0.66) | 0.42 (0.36, 0.48) | 0.02 (0.01, 0.02) | 0.07 (0.06, 0.1) |
| Middle SDI | 0.78 (0.69, 0.86) | 0.42 (0.36, 0.47) | 0.1 (0.08, 0.12) | 0.09 (0.08, 0.1) |
| High-middle SDI | 0.97 (0.9, 1.03) | 0.67 (0.62, 0.71) | 0.09 (0.08, 0.11) | 0.04 (0.04, 0.05) |
| High SDI | 7.07 (6.54, 7.71) | 5.47 (4.97, 6.06) | 0.44 (0.41, 0.52) | 0.62 (0.56, 0.73) |
| Andean Latin America | 0.52 (0.43, 0.64) | 0.12 (0.09, 0.16) | 0.04 (0.03, 0.05) | 0.26 (0.21, 0.32) |
| Australasia | 4.41 (3.94, 4.89) | 1.74 (1.51, 1.99) | 0.27 (0.23, 0.31) | 0.04 (0.03, 0.05) |
| Caribbean | 0.4 (0.34, 0.47) | 0.09 (0.07, 0.11) | 0.04 (0.03, 0.04) | 0.2 (0.16, 0.24) |
| Central Asia | 0.92 (0.78, 1.08) | 0.63 (0.52, 0.74) | 0.07 (0.06, 0.09) | 0.1 (0.07, 0.12) |
| Central Europe | 0.72 (0.67, 0.78) | 0.56 (0.52, 0.61) | 0.05 (0.04, 0.05) | 0.03 (0.02, 0.03) |
| Central Latin America | 0.43 (0.38, 0.48) | 0.09 (0.08, 0.1) | 0.04 (0.03, 0.05) | 0.21 (0.19, 0.24) |
| East Asia | 0.69 (0.57, 0.83) | 0.33 (0.26, 0.4) | 0.17 (0.14, 0.21) | 0.01 (0.01, 0.02) |
| Eastern Europe | 3.41 (3.11, 3.73) | 2.55 (2.33, 2.81) | 0.22 (0.2, 0.25) | 0.12 (0.11, 0.13) |
| Eastern Sub-Saharan Africa | 0.67 (0.46, 0.86) | 0.62 (0.43, 0.79) | 0.01 (0.01, 0.02) | 0.03 (0.01, 0.07) |
| High-income Asia Pacific | 0.17 (0.16, 0.18) | 0.12 (0.11, 0.13) | 0.01 (0.01, 0.01) | 0.02 (0.02, 0.02) |
| High-income North America | 18.42 (16.81, 20.33) | 14.5 (12.92, 16.3) | 1.15 (1.03, 1.39) | 1.75 (1.57, 2.11) |
| North Africa and Middle East | 1.24 (1.1, 1.42) | 0.81 (0.68, 0.93) | 0.04 (0.03, 0.06) | 0.11 (0.08, 0.14) |
| Oceania | 0.16 (0.12, 0.22) | 0.13 (0.09, 0.17) | 0.01 (0.01, 0.02) | 0.01 (0.01, 0.02) |
| South Asia | 0.64 (0.56, 0.72) | 0.48 (0.4, 0.55) | 0.02 (0.01, 0.02) | 0.06 (0.04, 0.09) |
| Southeast Asia | 0.34 (0.29, 0.42) | 0.25 (0.2, 0.3) | 0.03 (0.02, 0.05) | 0.02 (0.02, 0.03) |
| Southern Latin America | 0.2 (0.18, 0.23) | 0.14 (0.12, 0.16) | 0.01 (0.01, 0.01) | 0.03 (0.02, 0.03) |
| Southern Sub-Saharan Africa | 1.32 (1.2, 1.45) | 0.88 (0.78, 0.99) | 0.07 (0.05, 0.08) | 0.14 (0.11, 0.17) |
| Tropical Latin America | 0.57 (0.54, 0.62) | 0.03 (0.03, 0.04) | 0.01 (0.01, 0.01) | 0.42 (0.4, 0.46) |
| Western Europe | 2.28 (2.19, 2.37) | 1.67 (1.59, 1.74) | 0.14 (0.13, 0.15) | 0.11 (0.11, 0.12) |
| Western Sub-Saharan Africa | 0.05 (0.04, 0.07) | 0.05 (0.04, 0.07) | 0 (0, 0) | 0 (0, 0) |
