# Supplementary material for: Global burden of disease due to opioid, amphetamine, cocaine, and cannabis use disorders, 1990-2021: a systematic analysis for the Global Burden of Disease Study 2021
Source: PLoS One. 2025 Aug 21;20(8):e0328276. doi: 10.1371/journal.pone.0328276 (PMC12370144; doi:10.1371/journal.pone.0328276)
Supplement: S6 Table — (DOCX) [file pone.0328276.s007.docx]

**S6 Table. Age-standardized disability-adjusted life year (DALY) rates per 100,000 attributable to any, opioid, amphetamine, cocaine, and cannabis use disorders, stratified by world region, 1990-2021**

| **World region** | **Any drug use disorder, DALY rate (95% UI)** | **Opioid use disorder, DALY rate (95% UI)** | **Amphetamine use disorder, DALY rate (95% UI)** | **Cocaine use disorder, DALY rate (95% UI)** | **Cannabis use disorder, DALY rate (95% UI)** |
| --- | --- | --- | --- | --- | --- |
| Global | 190.97 (156.11, 222.79) | 137.15 (112.29, 161.39) | 20.98 (14.56, 29.33) | 13.88 (11.18, 17.52) | 8.27 (4.9, 12.86) |
| Low SDI | 66.47 (51.71, 81.05) | 50.81 (39.15, 61.76) | 4.36 (2.56, 6.63) | 2.62 (1.68, 4.07) | 6.06 (3.54, 9.54) |
| Low-middle SDI | 81.28 (63.74, 98.29) | 58.69 (44.52, 71.94) | 6.15 (3.75, 9.1) | 5.33 (4.09, 6.78) | 6.56 (3.88, 10.4) |
| Middle SDI | 112.9 (89.5, 135.09) | 63.6 (49.05, 77.63) | 23.63 (15.53, 34.41) | 8.82 (6.96, 11.21) | 7.49 (4.41, 11.73) |
| High-middle SDI | 153.92 (122.22, 185.21) | 98.7 (78.06, 119.48) | 29.48 (18.97, 43.15) | 7.96 (5.57, 11.14) | 7.69 (4.66, 11.79) |
| High SDI | 752.61 (630.61, 872.87) | 587.41 (484.84, 680.69) | 52.96 (40.45, 69.35) | 60.5 (48.28, 77.26) | 18.89 (11.53, 28.83) |
| Andean Latin America | 91.59 (71.54, 113.89) | 46.21 (32.59, 61.99) | 11.02 (6.9, 16.44) | 21.44 (16.63, 27.46) | 6.85 (4.03, 10.91) |
| Australasia | 464.24 (387.42, 539.76) | 205.48 (168.06, 240.26) | 81.67 (53.61, 117.56) | 32.77 (20.01, 51.15) | 20.65 (13.45, 31.16) |
| Caribbean | 92.3 (70.37, 116.99) | 40.77 (28.15, 53.52) | 8.49 (5.37, 12.56) | 24.47 (18.12, 33.54) | 13.73 (7.44, 22.62) |
| Central Asia | 158.77 (122.14, 192.69) | 117.72 (89.49, 144.38) | 20.19 (12.99, 29.11) | 8.07 (6.23, 10.21) | 5.78 (3.01, 9.7) |
| Central Europe | 113.79 (90.95, 137.08) | 64.41 (53.16, 75.8) | 26.84 (16.42, 40.57) | 6.34 (4.17, 9.49) | 9.98 (6.13, 15.25) |
| Central Latin America | 88.41 (68.83, 109.78) | 40.33 (28.12, 52.9) | 8.76 (5.72, 12.52) | 25.64 (18.93, 34.27) | 7.96 (4.96, 11.83) |
| East Asia | 117.23 (89.99, 144.5) | 54.37 (40.97, 67.47) | 44.67 (29.64, 65.46) | 1.51 (0.99, 2.13) | 6.02 (3.44, 9.65) |
| Eastern Europe | 403.12 (337.84, 468.51) | 311.15 (259.01, 365.83) | 39.53 (28.6, 53.32) | 13.06 (10.35, 16.89) | 9.78 (5.45, 15.57) |
| Eastern Sub-Saharan Africa | 66.58 (51.76, 82.03) | 51.62 (40.04, 62.98) | 5.24 (3.06, 8.03) | 2.35 (1.45, 4.26) | 6.27 (3.62, 10.11) |
| High-income Asia Pacific | 90.07 (65.11, 117.63) | 43.74 (30.9, 57.08) | 15.2 (8.7, 23.86) | 14.74 (8.79, 23.21) | 13.53 (7.7, 22.38) |
| High-income North America | 1836.34 (1547.74, 2122.45) | 1502.44 (1235.96, 1740.1) | 98.53 (79.65, 122.87) | 147.83 (121.82, 183.99) | 27.88 (16.97, 42.78) |
| North Africa and Middle East | 161.95 (129.92, 193.35) | 128.78 (99.59, 157.46) | 6.83 (4.51, 9.43) | 7.17 (5.5, 9.34) | 3.9 (2.27, 6.24) |
| Oceania | 68.81 (49.71, 89.73) | 34.29 (24.39, 44.64) | 18.44 (10.09, 30.05) | 0.92 (0.65, 1.41) | 13.18 (7.15, 22.17) |
| South Asia | 78.68 (62.13, 95.44) | 61.71 (46.68, 75.92) | 2.39 (1.63, 3.36) | 2.91 (2.02, 4.03) | 7.48 (4.47, 11.97) |
| Southeast Asia | 71.21 (52.71, 91.13) | 32.9 (25.23, 40.51) | 26.84 (15.71, 41.66) | 1.13 (0.84, 1.66) | 7.85 (4.52, 12.65) |
| Southern Latin America | 110.29 (79.08, 143.92) | 51.7 (36.07, 67.75) | 10.07 (5.93, 15.92) | 35.08 (21.05, 53.62) | 10.67 (7.19, 15.54) |
| Southern Sub-Saharan Africa | 143.29 (117, 169.05) | 88.95 (71.72, 105.11) | 17.5 (11.31, 24.89) | 17.28 (12.43, 23.48) | 8.39 (4.76, 13.56) |
| Tropical Latin America | 129.66 (98.32, 160.11) | 38.51 (25.72, 51.57) | 23.26 (13.32, 36.42) | 49.14 (38.4, 63.14) | 12.1 (7.27, 19.12) |
| Western Europe | 276.35 (230.25, 322.25) | 178.12 (149.99, 207.3) | 32.15 (21.68, 45.88) | 24.7 (16.67, 35.05) | 17.31 (11.24, 25.56) |
| Western Sub-Saharan Africa | 37.27 (26.73, 48.09) | 26.84 (18.22, 35.3) | 4.55 (2.54, 7.32) | 1.27 (0.87, 1.81) | 3.63 (2.08, 5.73) |
