# Supplementary material for: Global burden of disease due to opioid, amphetamine, cocaine, and cannabis use disorders, 1990-2021: a systematic analysis for the Global Burden of Disease Study 2021
Source: PLoS One. 2025 Aug 21;20(8):e0328276. doi: 10.1371/journal.pone.0328276 (PMC12370144; doi:10.1371/journal.pone.0328276)
Supplement: S7 Table — (DOCX) [file pone.0328276.s008.docx]

**S7 Table. Age-standardized disability-adjusted life year (DALY) rates per 100,000 attributable to any drug use disorder, stratified by country in 1990 and 2021, and total percentage change**

| **Location** | **DALY rate (95% UI) in 1990** | **DALY rate (95% UI) in 2021** | **% Change** |
| --- | --- | --- | --- |
| Afghanistan | 186.58 (145.33, 228.33) | 203.76 (162.87, 250.12) | 8.81 |
| Albania | 93.43 (73.86, 115.37) | 125.5 (97.97, 155.66) | 29.51 |
| Algeria | 133.79 (106.04, 166.23) | 167.13 (129.49, 207.11) | 22.25 |
| American Samoa | 74.42 (54.39, 97.12) | 78.45 (56.67, 99.53) | 5.27 |
| Andorra | 99.48 (70.88, 131.1) | 105 (74.81, 137.71) | 5.40 |
| Angola | 55.06 (40.52, 69.71) | 60.5 (44.59, 77.78) | 9.42 |
| Antigua and Barbuda | 85.28 (60.35, 112.26) | 89.05 (66.09, 113.69) | 4.33 |
| Argentina | 103.91 (70.08, 140.39) | 105.17 (74.47, 140.02) | 1.21 |
| Armenia | 124.44 (88.42, 161.92) | 113.38 (79.07, 145.88) | -9.31 |
| Australia | 399.17 (324.22, 470.93) | 502.09 (420.6, 582.79) | 22.94 |
| Austria | 200.99 (155.81, 245.3) | 251.89 (204.95, 297.04) | 22.57 |
| Azerbaijan | 114.06 (80.39, 147.76) | 105.87 (77.21, 135.95) | -7.45 |
| Bahamas | 91.26 (67.93, 120.58) | 92.01 (66.36, 120.85) | 0.82 |
| Bahrain | 95.62 (68.03, 126.16) | 93.48 (67.05, 120.03) | -2.26 |
| Bangladesh | 61.28 (46.17, 78.23) | 64.28 (48.17, 82.75) | 4.78 |
| Barbados | 89.93 (62.68, 120.41) | 89.49 (64.57, 116.73) | -0.49 |
| Belarus | 241.42 (178.96, 311.95) | 249.96 (203.26, 299.17) | 3.48 |
| Belgium | 135.58 (105.48, 168.1) | 214.45 (177.84, 253) | 45.85 |
| Belize | 82.2 (58.48, 108.76) | 80.53 (57.42, 107.07) | -2.05 |
| Benin | 39.11 (27.24, 52.25) | 39.58 (28.24, 52.56) | 1.19 |
| Bermuda | 110.85 (83.7, 141.53) | 126.52 (97.57, 155.03) | 13.22 |
| Bhutan | 60.85 (45.45, 79.24) | 65.05 (48.01, 83.53) | 6.67 |
| Bolivia | 97.74 (72.52, 122.82) | 99.48 (74.1, 125.62) | 1.76 |
| Bosnia and Herzegovina | 57.23 (41.81, 73.6) | 62.65 (45.37, 81.24) | 9.05 |
| Botswana | 91.73 (67.41, 117.34) | 88.09 (65.96, 110.86) | -4.05 |
| Brazil | 102.77 (71.68, 134.29) | 131.43 (99.94, 162.15) | 24.60 |
| Brunei Darussalam | 141.63 (108.6, 174.48) | 112.67 (84.86, 140.97) | -22.88 |
| Bulgaria | 93.68 (71.23, 118.27) | 130.03 (101.41, 161.12) | 32.79 |
| Burkina Faso | 36.85 (26.19, 48.02) | 38.59 (26.98, 50.61) | 4.61 |
| Burundi | 69.16 (50.63, 94.44) | 71.49 (50.65, 96.4) | 3.31 |
| Cabo Verde | 45.49 (32.37, 60) | 44.78 (31.6, 58.3) | -1.57 |
| Cambodia | 60.78 (43.82, 80.82) | 60.94 (43.08, 81.77) | 0.26 |
| Cameroon | 44.31 (31.76, 58.1) | 43.97 (30.73, 57.49) | -0.77 |
| Canada | 346.59 (265.58, 430.44) | 877.19 (759.19, 998.21) | 92.86 |
| Central African Republic | 54.85 (39.36, 72.3) | 54.61 (38.63, 69.96) | -0.44 |
| Chad | 39.65 (27.37, 52.08) | 37.39 (25.9, 49.29) | -5.87 |
| Chile | 102.2 (72.67, 134.01) | 121.03 (89.38, 154.42) | 16.91 |
| China | 272.83 (220.61, 321.52) | 116.47 (89.13, 143.59) | -85.12 |
| Colombia | 94.27 (70.11, 120.64) | 96.46 (73.86, 123.08) | 2.30 |
| Comoros | 68.76 (46.25, 92.21) | 82.95 (61.11, 109.32) | 18.76 |
| Congo | 60.99 (45.44, 76.91) | 66.07 (49.55, 83.27) | 8.00 |
| Cook Islands | 70.76 (49.12, 94.02) | 71.35 (50.48, 91.88) | 0.83 |
| Costa Rica | 78.5 (56.71, 101.67) | 85.71 (66.16, 107.9) | 8.79 |
| Côte d'Ivoire | 39.59 (27.92, 52.68) | 179.36 (142.59, 214.5) | 151.08 |
| Croatia | 128.87 (103.24, 155.02) | 78.16 (55.3, 102.49) | -50.00 |
| Cuba | 92.18 (65.71, 119.95) | 152.61 (119.09, 186.84) | 50.41 |
| Cyprus | 146.07 (115.48, 178.24) | 143.42 (113.3, 176.56) | -1.83 |
| Czechia | 101.69 (78, 127.98) | 38.76 (26.67, 50.76) | -96.45 |
| Democratic People's Republic of Korea | 133.18 (102.78, 166.23) | 113.5 (85.73, 146.3) | -15.99 |
| Republic of the Congo | 51.01 (37.94, 66.17) | 58.25 (42.28, 75.62) | 13.27 |
| Denmark | 339.41 (283.8, 400.08) | 378.43 (314.58, 437.72) | 10.88 |
| Djibouti | 71.07 (49.49, 95.31) | 84.04 (58.17, 112.06) | 16.76 |
| Dominica | 94.29 (68.33, 124.08) | 110.45 (83.99, 140.26) | 15.82 |
| Dominican Republic | 62.47 (43.47, 82.8) | 69.62 (48.97, 92.23) | 10.84 |
| Ecuador | 76.57 (55.49, 98.07) | 94 (73.58, 116.86) | 20.51 |
| Egypt | 84.31 (57.37, 111.06) | 99.09 (68.94, 128.83) | 16.15 |
| El Salvador | 84.95 (66.85, 106.25) | 81.5 (62.5, 101.45) | -4.15 |
| Equatorial Guinea | 55.41 (41.67, 73.03) | 68.49 (50.91, 89.21) | 21.19 |
| Eritrea | 69.14 (50.71, 93.3) | 85.17 (59.14, 117.54) | 20.85 |
| Estonia | 315.86 (258.12, 366.21) | 733.92 (586.79, 889.92) | 84.31 |
| Eswatini | 94.5 (70.82, 118.94) | 100.77 (78.29, 125.21) | 6.42 |
| Ethiopia | 47.88 (36.62, 62.07) | 45.56 (34.27, 57.71) | -4.97 |
| Fiji | 71.86 (51.64, 94.58) | 64.06 (45.16, 84.29) | -11.49 |
| Finland | 273.71 (231.04, 315.97) | 517.31 (441.03, 595.09) | 63.66 |
| France | 140.69 (111.8, 171.62) | 200.59 (165.19, 237.62) | 35.47 |
| Gabon | 67.43 (51.12, 85.07) | 69.92 (52.73, 89.23) | 3.63 |
| Gambia | 43.41 (29.72, 57.15) | 40.46 (28.72, 52.18) | -7.04 |
| Georgia | 123.99 (88.26, 162.37) | 119.48 (87.84, 151.18) | -3.71 |
| Germany | 194.12 (159.04, 228.19) | 235.19 (192.38, 277.4) | 19.19 |
| Ghana | 43.82 (29.9, 58.07) | 44.81 (30.18, 59.47) | 2.23 |
| Greece | 138.98 (109.01, 168.52) | 256.32 (212.32, 296.43) | 61.21 |
| Greenland | 282.91 (221.18, 346.83) | 332.53 (267.32, 400.83) | 16.16 |
| Grenada | 91.59 (66.71, 120.04) | 119.18 (91.04, 149.39) | 26.33 |
| Guam | 87.71 (65.75, 113.56) | 73.34 (51.31, 96.78) | -17.89 |
| Guatemala | 117.52 (99.96, 135.9) | 124.14 (104.01, 145.53) | 5.48 |
| Guinea | 36.9 (25.8, 48.04) | 35.54 (25.19, 46.4) | -3.76 |
| Guinea-Bissau | 37.94 (26.7, 50.46) | 36.97 (26.06, 48.15) | -2.59 |
| Guyana | 69.69 (49.25, 92.12) | 73.59 (53.67, 95.06) | 5.45 |
| Haiti | 77.46 (56.62, 99.34) | 85.99 (63.57, 110.51) | 10.45 |
| Honduras | 103.91 (84.4, 125.53) | 109.67 (83.47, 138.69) | 5.40 |
| Hungary | 90.67 (65.79, 117.38) | 109.92 (82.51, 140.09) | 19.25 |
| Iceland | 255.14 (208.42, 302) | 510.21 (432.42, 583.44) | 69.30 |
| India | 71.92 (56.97, 87.98) | 81.52 (64.47, 98.94) | 12.53 |
| Indonesia | 43.78 (30.28, 59.82) | 47.44 (33.63, 64.09) | 8.03 |
| Iran | 339.74 (281.57, 397.18) | 339.36 (282.07, 397.38) | -0.11 |
| Iraq | 137.77 (108.2, 169.29) | 159.03 (125.92, 202.94) | 14.35 |
| Ireland | 180.28 (145.99, 220.03) | 414.53 (342.12, 481.53) | 83.26 |
| Israel | 129.87 (99.73, 158.19) | 153.65 (122.92, 184.31) | 16.81 |
| Italy | 262.19 (213.15, 312.22) | 138.92 (104.24, 173.13) | -63.52 |
| Jamaica | 90.87 (65.5, 118.73) | 89.88 (65.52, 118.55) | -1.10 |
| Japan | 85.95 (61.12, 113.56) | 85.3 (62.08, 111.73) | -0.76 |
| Jordan | 94.55 (65.81, 123.25) | 90.59 (63.86, 119.88) | -4.28 |
| Kazakhstan | 189.55 (147.86, 233.05) | 314.08 (249.39, 378.82) | 50.50 |
| Kenya | 45.29 (33.32, 56.53) | 48.6 (37.5, 58.62) | 7.05 |
| Kiribati | 259.38 (209.52, 319.63) | 322.76 (242.47, 419.61) | 21.86 |
| Kuwait | 106.42 (78.85, 138.35) | 128.51 (98.92, 162.26) | 18.86 |
| Kyrgyzstan | 161.32 (126.42, 198.84) | 166.43 (134.29, 204.05) | 3.12 |
| Lao People's Democratic Republic | 57.8 (41.42, 75.92) | 56.93 (40.92, 74.67) | -1.52 |
| Latvia | 229.13 (181.71, 270.11) | 272.59 (220.7, 323.57) | 17.37 |
| Lebanon | 107.35 (79.07, 136.87) | 141.39 (104.26, 179.73) | 27.54 |
| Lesotho | 85.11 (62.46, 108.35) | 96.68 (73.24, 122.71) | 12.75 |
| Liberia | 42.02 (29.64, 55.62) | 41.44 (29.48, 55.64) | -1.39 |
| Libya | 146.97 (116.72, 179.59) | 251.47 (204.48, 310.75) | 53.71 |
| Lithuania | 201.04 (160.17, 235.54) | 386.6 (317.58, 458.25) | 65.39 |
| Luxembourg | 271.05 (221.32, 318.46) | 261.12 (213.41, 307.89) | -3.73 |
| Madagascar | 64.61 (48.51, 84.26) | 69.86 (51.13, 91.07) | 7.81 |
| Malawi | 66.67 (48.95, 89.28) | 79.89 (57.75, 106.44) | 18.09 |
| Malaysia | 68.1 (47.71, 89.57) | 67.79 (48.14, 88.82) | -0.46 |
| Maldives | 63.54 (45.07, 85.07) | 76.24 (53.87, 98.94) | 18.22 |
| Mali | 38.49 (26.58, 50.17) | 36.87 (25.47, 48.51) | -4.30 |
| Malta | 140.23 (107.69, 171.52) | 226.98 (182.96, 268.43) | 48.16 |
| Marshall Islands | 77.78 (57.33, 99.98) | 76.99 (56.01, 99.43) | -1.02 |
| Mauritania | 43.17 (30.31, 57.31) | 42 (28.27, 55.52) | -2.75 |
| Mauritius | 92.85 (64.41, 122.79) | 180.59 (150.11, 213.3) | 66.52 |
| Mexico | 85.83 (64.9, 109.36) | 86.88 (66.56, 108.77) | 1.22 |
| Micronesia | 80.86 (59.31, 103.52) | 77.86 (55.88, 100.16) | -3.78 |
| Monaco | 116.23 (80.41, 152.32) | 128.07 (92.24, 165.49) | 9.70 |
| Mongolia | 114.09 (80.19, 147.85) | 129.55 (96.31, 163.39) | 12.71 |
| Montenegro | 71.49 (51.99, 92.41) | 72.47 (52.23, 94.27) | 1.36 |
| Morocco | 140.09 (107.8, 175.01) | 165.53 (128.5, 206.81) | 16.69 |
| Mozambique | 46.73 (34.85, 58.99) | 64.16 (48.75, 82.74) | 31.70 |
| Myanmar | 65.89 (47.11, 87.85) | 61.29 (42.51, 81.05) | -7.24 |
| Namibia | 95.67 (71.45, 122.29) | 96.73 (73.34, 119.31) | 1.10 |
| Nauru | 79.19 (57.68, 103.19) | 76.29 (54.26, 98.73) | -3.73 |
| Nepal | 63.57 (47.44, 81.53) | 63.95 (47.6, 81.63) | 0.60 |
| Netherlands | 126.71 (94.18, 160.63) | 168.58 (133.69, 205.61) | 28.55 |
| New Zealand | 197.94 (146.45, 249.87) | 273.84 (217.67, 327.59) | 32.46 |
| Nicaragua | 71.06 (50.77, 92.33) | 63.46 (45.27, 80.97) | -11.31 |
| Niger | 39.11 (27.35, 53.26) | 38.58 (26.92, 51.9) | -1.36 |
| Nigeria | 34.86 (25.36, 44.42) | 33.93 (24.41, 43.68) | -2.70 |
| Niue | 79.2 (57.65, 101.55) | 75.32 (53.91, 97.89) | -5.02 |
| North Macedonia | 86.68 (63.23, 111.32) | 96.5 (71.81, 121.5) | 10.73 |
| Northern Mariana Islands | 84.09 (61.15, 109.94) | 86.24 (61.67, 113.89) | 2.52 |
| Norway | 276.95 (224.16, 331.68) | 466.2 (387.62, 544.62) | 52.08 |
| Oman | 93.09 (67, 121.43) | 89.8 (64.7, 115.19) | -3.60 |
| Pakistan | 75.25 (58.15, 94.47) | 72.36 (56.7, 88.86) | -3.92 |
| Palau | 69.63 (48.11, 92.92) | 72.25 (48.71, 95.44) | 3.69 |
| Palestine | 91.66 (62.55, 124.23) | 84.69 (56.46, 114.03) | -7.91 |
| Panama | 81.47 (59.97, 104.45) | 82.51 (62.54, 102.86) | 1.27 |
| Papua New Guinea | 66.36 (46.28, 89.06) | 65.57 (46.23, 87.11) | -1.20 |
| Paraguay | 74.34 (50.87, 98.76) | 78.01 (56.42, 101.02) | 4.82 |
| Peru | 81.89 (61.06, 104.08) | 87.87 (68.13, 109.57) | 7.05 |
| Philippines | 60.04 (42.96, 78.15) | 57.95 (41.23, 74.9) | -3.54 |
| Poland | 109.63 (83.82, 136.69) | 115.61 (90.21, 142.29) | 5.31 |
| Portugal | 133.31 (104.49, 160.3) | 138.2 (110.96, 165.59) | 3.60 |
| Puerto Rico | 165.36 (122.48, 205.14) | 251.83 (212.35, 298.31) | 42.06 |
| Qatar | 92.01 (66.28, 121.21) | 87.42 (63.03, 116.31) | -5.12 |
| Republic of Korea | 99.29 (70.42, 129.96) | 99.31 (69.68, 130.25) | 0.02 |
| Republic of Moldova | 162.24 (121.95, 201.48) | 154.35 (119.94, 191.23) | -4.99 |
| Romania | 88.48 (69.61, 108.59) | 86.52 (71.04, 102.34) | -2.24 |
| Russian Federation | 371.69 (295.89, 440.78) | 456.02 (385.23, 526.69) | 20.45 |
| Rwanda | 76.81 (57.99, 102.27) | 80.54 (57.06, 106.74) | 4.74 |
| Saint Kitts and Nevis | 84.88 (61.16, 111.55) | 85.06 (61.34, 108.84) | 0.21 |
| Saint Lucia | 90.66 (62.86, 120.03) | 87.99 (62.82, 112.5) | -2.99 |
| Saint Vincent and the Grenadines | 78.8 (54.27, 104.76) | 84.79 (61.41, 111) | 7.33 |
| Samoa | 77.45 (55.55, 100.94) | 78.49 (57.44, 101.01) | 1.33 |
| San Marino | 115.63 (81.32, 153.89) | 119.6 (86.43, 156.97) | 3.38 |
| Sao Tome and Principe | 47.81 (34.06, 62.33) | 53.84 (39.92, 68.23) | 11.88 |
| Saudi Arabia | 90.45 (61.09, 118.41) | 93.75 (65.88, 124.07) | 3.58 |
| Senegal | 43.45 (30.68, 57.11) | 40.25 (27.73, 53.38) | -7.65 |
| Serbia | 88.22 (70.84, 108.46) | 105.93 (83.54, 127.73) | 18.29 |
| Seychelles | 87.45 (64.41, 111.77) | 100.58 (76.69, 124.08) | 13.99 |
| Sierra Leone | 43.88 (30.89, 57.28) | 40.61 (28.44, 53.56) | -7.74 |
| Singapore | 95.99 (67.14, 127.03) | 94.91 (66.68, 126.65) | -1.13 |
| Slovakia | 113.36 (85.21, 140.66) | 105.55 (80.68, 130.45) | -7.14 |
| Slovenia | 112.48 (92.1, 135.21) | 178.61 (141.47, 215.11) | 46.24 |
| Solomon Islands | 70.93 (51.46, 92.02) | 70.61 (51.45, 91.58) | -0.45 |
| Somalia | 65.49 (46.35, 95.44) | 65.97 (44.87, 95.54) | 0.73 |
| South Africa | 201.24 (159.91, 240.46) | 157.73 (129.31, 185.46) | -24.36 |
| South Sudan | 65.4 (47.63, 86.7) | 66.87 (48.19, 94.65) | 2.22 |
| Spain | 259.39 (210.45, 311.4) | 218.13 (170.99, 268) | -17.32 |
| Sri Lanka | 121.71 (94.23, 146.76) | 88.35 (66.28, 111.91) | -32.03 |
| Sudan | 126.87 (97.46, 159.88) | 161.57 (124.37, 198.88) | 24.18 |
| Suriname | 78.46 (56.43, 101.83) | 100.07 (76.21, 126.53) | 24.33 |
| Sweden | 125.47 (103.75, 147.84) | 430.88 (354.39, 505.09) | 123.38 |
| Switzerland | 553.92 (451.47, 652.04) | 269.87 (224.64, 317.7) | -71.91 |
| Syrian Arab Republic | 113.84 (89.17, 141.36) | 134.46 (103.85, 164.48) | 16.65 |
| Taiwan (Province of China) | 153.93 (126.31, 182.7) | 165.5 (130.5, 203.15) | 7.25 |
| Tajikistan | 111.75 (79.52, 146.65) | 103.68 (75.1, 130.88) | -7.50 |
| Thailand | 96.15 (65.07, 132.14) | 103.34 (72.4, 136.78) | 7.21 |
| Timor-Leste | 63.17 (43.43, 85.3) | 63.34 (44.08, 85.88) | 0.27 |
| Togo | 36.25 (24.77, 47.7) | 37.8 (26.1, 49.61) | 4.19 |
| Tokelau | 76.42 (56.09, 100.03) | 74.47 (52.98, 95.36) | -2.58 |
| Tonga | 71.42 (50.64, 94.16) | 72.51 (51.89, 95.98) | 1.51 |
| Trinidad and Tobago | 78.99 (55.07, 104.1) | 97.73 (73.59, 122.05) | 21.29 |
| Tunisia | 129.05 (100.36, 161.74) | 165.06 (127.32, 203.38) | 24.61 |
| Türkiye | 104.03 (75.48, 136.23) | 215.12 (164.1, 270.38) | 72.65 |
| Turkmenistan | 121.97 (87.65, 156.87) | 74.55 (53.49, 98.2) | -49.23 |
| Tuvalu | 74.8 (53.99, 96.41) | 104.49 (76.03, 132.91) | 33.43 |
| Uganda | 65.87 (47.32, 86.26) | 77.94 (57.52, 102.99) | 16.83 |
| Ukraine | 223.44 (180.95, 267.11) | 279.62 (219.77, 344.82) | 22.43 |
| United Arab Emirates | 133.16 (101.2, 169.22) | 200.82 (149.9, 252.32) | 41.09 |
| United Kingdom | 243.89 (191.8, 294.14) | 554.29 (472.29, 632.12) | 82.10 |
| United Republic of Tanzania | 82.51 (59.88, 109.61) | 95.07 (68.53, 125.5) | 14.17 |
| United States of America | 352.71 (272.69, 429.65) | 221.66 (170.49, 294.74) | -46.45 |
| United States Virgin Islands | 164.08 (132.75, 198.59) | 1944.08 (1632.99, 2249.41) | 247.22 |
| Uruguay | 120.78 (85.22, 160.32) | 122.11 (89.36, 155.52) | 1.10 |
| Uzbekistan | 105.49 (74.75, 138.41) | 107.79 (77.72, 134.72) | 2.16 |
| Vanuatu | 67.97 (49.15, 88.79) | 66.53 (47.13, 86.28) | -2.14 |
| Venezuela | 69.35 (49.93, 91.04) | 63.7 (45.37, 82.72) | -8.50 |
| Vietnam | 104.09 (81.28, 129.59) | 140.56 (107.02, 176.8) | 30.04 |
| Yemen | 106.37 (78.72, 141.04) | 133.48 (98.84, 174.39) | 22.70 |
| Zambia | 81.46 (60.77, 107.02) | 90.76 (66.66, 120.63) | 10.81 |
| Zimbabwe | 105.2 (79.08, 133.93) | 106.19 (81.88, 130.44) | 0.94 |
