# Supplementary material for: Global burden of disease due to opioid, amphetamine, cocaine, and cannabis use disorders, 1990-2021: a systematic analysis for the Global Burden of Disease Study 2021
Source: PLoS One. 2025 Aug 21;20(8):e0328276. doi: 10.1371/journal.pone.0328276 (PMC12370144; doi:10.1371/journal.pone.0328276)
Supplement: S8 Table — (DOCX) [file pone.0328276.s009.docx]

**S8 Table. Age-standardized disability-adjusted life year (DALY) rates per 100,000 attributable to any opioid use disorder, stratified by country in 1990 and 2021, and total percentage change**

| **Location** | **DALY rate (95% UI) in 1990** | **DALY rate (95% UI) in 2021** | **% Change** |
| --- | --- | --- | --- |
| Afghanistan | 144.75 (105.44, 183.81) | 160.36 (123.62, 202.29) | 10.24 |
| Albania | 56.29 (43.22, 70.13) | 84.72 (66.28, 105.4) | 40.88 |
| Algeria | 103.43 (78.56, 129.94) | 130.64 (98.89, 164.79) | 23.36 |
| American Samoa | 38.77 (27.27, 50.63) | 39.17 (28.2, 50.95) | 1.03 |
| Andorra | 45.89 (30.68, 61.84) | 49.5 (33.43, 65.45) | 7.57 |
| Angola | 41.51 (30.56, 53.82) | 46.1 (33.64, 59.94) | 10.49 |
| Antigua and Barbuda | 43.63 (28.74, 59.64) | 41.48 (27.64, 56.52) | -5.05 |
| Argentina | 51.95 (33.06, 70.15) | 50.12 (34.7, 66.96) | -3.59 |
| Armenia | 97.23 (65.02, 131.06) | 84.59 (57.34, 112.66) | -13.93 |
| Australia | 218.95 (183.12, 253.54) | 226.75 (185.97, 264.86) | 3.50 |
| Austria | 144.43 (115.1, 173.25) | 176.72 (144.89, 207.13) | 20.18 |
| Azerbaijan | 88.45 (60.9, 117.49) | 79.06 (57.49, 103.56) | -11.22 |
| Bahamas | 46.72 (31.74, 63.54) | 43.7 (29.12, 58.49) | -6.68 |
| Bahrain | 81.25 (56.69, 108.37) | 77.97 (54.27, 102.55) | -4.12 |
| Bangladesh | 48.78 (35.94, 63.86) | 51.83 (37.3, 67.72) | 6.06 |
| Barbados | 50.61 (32.8, 69.58) | 46.27 (30.77, 63.15) | -8.97 |
| Belarus | 191.91 (138.5, 253.05) | 194.51 (157.13, 231.25) | 1.35 |
| Belgium | 77.22 (59.55, 93.97) | 134.97 (113.08, 158.89) | 55.84 |
| Belize | 41.57 (27.34, 57.33) | 38.34 (25.45, 52.81) | -8.09 |
| Benin | 28.29 (18.71, 38.44) | 28.24 (19.13, 38.21) | -0.18 |
| Bermuda | 57.97 (40.77, 75.73) | 56.94 (41.96, 74.79) | -1.79 |
| Bhutan | 48.58 (35.42, 63.87) | 52.19 (37.22, 68.26) | 7.17 |
| Bolivia | 51.4 (34.91, 69.15) | 46.5 (31.64, 62.25) | -10.02 |
| Bosnia and Herzegovina | 38.54 (26.84, 50.82) | 44.19 (31.06, 59.09) | 13.68 |
| Botswana | 72.82 (53.44, 94.77) | 69.74 (51.19, 88.14) | -4.32 |
| Brazil | 41.19 (27.12, 55.76) | 38.39 (25.67, 51.43) | -7.04 |
| Brunei Darussalam | 80.5 (61.64, 99.12) | 56.67 (43.12, 71.25) | -35.10 |
| Bulgaria | 48.22 (35.94, 60.94) | 76.69 (60.43, 93.73) | 46.40 |
| Burkina Faso | 26.89 (18.08, 35.92) | 28.58 (18.9, 37.94) | 6.10 |
| Burundi | 54.38 (38.91, 75.53) | 57.27 (39.31, 79.18) | 5.18 |
| Cabo Verde | 35.32 (24.11, 47.47) | 33.26 (22.63, 44.06) | -6.01 |
| Cambodia | 27.39 (20.27, 36.08) | 28.01 (20.06, 36.62) | 2.24 |
| Cameroon | 33.86 (23.16, 45.47) | 32.98 (22.56, 44.06) | -2.63 |
| Canada | 228.61 (172.36, 283.43) | 681.03 (590.51, 775.86) | 109.16 |
| Central African Republic | 41.74 (28.99, 57.02) | 41.33 (28.78, 54) | -0.99 |
| Chad | 29.49 (19.64, 39.54) | 27.25 (18.34, 36.28) | -7.90 |
| Chile | 50.12 (31.9, 69.11) | 54.32 (38.31, 71.4) | 8.05 |
| China | 156.25 (122.44, 184.48) | 53.97 (40.54, 66.99) | -106.30 |
| Colombia | 46.21 (30.59, 62.75) | 43.8 (29.27, 59.7) | -5.36 |
| Comoros | 53.63 (33.34, 72.59) | 66.71 (47.82, 88.87) | 21.82 |
| Congo | 46.42 (33.79, 59.95) | 50.12 (37.17, 63.97) | 7.67 |
| Cook Islands | 35.98 (24.93, 47.73) | 34.95 (24.19, 45.74) | -2.90 |
| Costa Rica | 43.99 (28.9, 59.89) | 44.53 (31.11, 60.18) | 1.22 |
| Côte d'Ivoire | 29.15 (20.34, 39.7) | 28.09 (18.69, 37.57) | -3.70 |
| Croatia | 83.37 (65.2, 101.78) | 135.52 (108.14, 162.73) | 48.58 |
| Cuba | 50.91 (33.04, 70.42) | 41.22 (26.89, 57.7) | -21.11 |
| Cyprus | 91.05 (73.19, 109.86) | 95.16 (74.28, 116.35) | 4.42 |
| Czechia | 45.33 (34.75, 56.34) | 78.93 (61.64, 95.17) | 55.46 |
| Democratic People's Republic of Korea | 71.68 (55.22, 89.14) | 56.48 (42.26, 72.34) | -23.83 |
| Republic of the Congo | 234.16 (196.51, 275.77) | 253.67 (211.5, 294.89) | 8.00 |
| Denmark | 56.13 (37.25, 78.3) | 67.93 (45, 93.28) | 19.08 |
| Djibouti | 39.13 (25.51, 54.14) | 38.71 (26.56, 51.61) | -1.08 |
| Dominica | 32.41 (21.3, 43.93) | 35.44 (22.39, 48.85) | 8.94 |
| Dominican Republic | 43 (27.99, 58.96) | 46.89 (32.68, 62.02) | 8.66 |
| Ecuador | 73.65 (49.91, 98.62) | 86.87 (60.04, 114.44) | 16.51 |
| Egypt | 47.32 (32.62, 62.46) | 44.94 (31.19, 58.8) | -5.16 |
| El Salvador | 42.08 (30.82, 55.93) | 50.91 (36.2, 66.61) | 19.05 |
| Equatorial Guinea | 54.38 (37.74, 74.09) | 68.55 (45.81, 95.87) | 23.16 |
| Eritrea | 230.41 (181.76, 269.14) | 611.05 (481.07, 739.56) | 97.53 |
| Estonia | 71.5 (51.76, 92.37) | 75.7 (57.24, 96.47) | 5.71 |
| Eswatini | 32.99 (24.43, 43.22) | 31.7 (23.56, 40.23) | -3.99 |
| Ethiopia | 37 (27.2, 47.82) | 30.29 (21.3, 39.76) | -20.01 |
| Fiji | 188.91 (161.51, 217.38) | 373.86 (313.5, 429.27) | 68.26 |
| Finland | 91.82 (72.31, 112.12) | 137.26 (112, 162.29) | 40.20 |
| France | 52.51 (39.36, 66.89) | 53.04 (39.43, 68.44) | 1.00 |
| Gabon | 33.08 (21.84, 44.81) | 30.07 (20.53, 40.26) | -9.54 |
| Gambia | 96.06 (65.77, 127.61) | 88.55 (64.38, 114.37) | -8.14 |
| Georgia | 132.75 (112.14, 152.35) | 150.93 (122.82, 176.42) | 12.83 |
| Germany | 32.99 (21.64, 44.71) | 33.45 (21.8, 45.42) | 1.38 |
| Ghana | 111.22 (86.31, 135.39) | 214.28 (176.9, 250.66) | 65.58 |
| Greece | 168.64 (132.12, 208.92) | 219.27 (173.03, 259.16) | 26.25 |
| Greenland | 43.29 (28.81, 59.63) | 42.91 (30.49, 56.4) | -0.88 |
| Grenada | 47.23 (35.5, 59.59) | 35.19 (23.53, 46.34) | -29.43 |
| Guam | 56.65 (44.13, 70.23) | 50.54 (38.53, 64.85) | -11.41 |
| Guatemala | 26.71 (17.73, 35.39) | 25.31 (16.99, 34.22) | -5.38 |
| Guinea | 27.8 (19.3, 37.93) | 26.73 (18.15, 35.94) | -3.92 |
| Guinea-Bissau | 38.65 (25.14, 53.26) | 36.29 (24.02, 49.3) | -6.30 |
| Guyana | 36.25 (25.39, 48.61) | 35.71 (23.62, 48.31) | -1.50 |
| Haiti | 52.69 (37.7, 67.27) | 52.57 (37.25, 69.11) | -0.23 |
| Honduras | 43.08 (31.07, 56.48) | 58.69 (45.25, 73.23) | 30.92 |
| Hungary | 168.51 (138.2, 198.72) | 353.6 (297.55, 407.25) | 74.12 |
| Iceland | 55.89 (42.47, 67.85) | 63.83 (47.77, 78.66) | 13.28 |
| India | 13.08 (9.51, 16.91) | 15.2 (11.76, 19.23) | 15.02 |
| Indonesia | 252.73 (195.63, 301.11) | 256.77 (204.65, 306.48) | 1.59 |
| Iran | 100.07 (73.43, 127.13) | 108.4 (82.76, 139.92) | 8.00 |
| Iraq | 103.66 (82.33, 126.2) | 289.26 (239.3, 340.1) | 102.62 |
| Ireland | 71.98 (53.27, 89.18) | 81.49 (62.69, 100.66) | 12.41 |
| Israel | 183.04 (152.55, 214.3) | 67.55 (52.33, 82) | -99.68 |
| Italy | 42.54 (28.66, 57.12) | 39.53 (25.21, 53.66) | -7.34 |
| Jamaica | 41.18 (27.5, 54.26) | 40.63 (28.84, 53.14) | -1.34 |
| Japan | 79.39 (53.59, 106.03) | 77.75 (52.78, 105.49) | -2.09 |
| Jordan | 151.82 (117.06, 188.65) | 245.1 (191.86, 302.6) | 47.90 |
| Kazakhstan | 34.28 (25.56, 42.45) | 35.56 (26.94, 43.78) | 3.67 |
| Kenya | 176.11 (138.44, 221.26) | 203.08 (147.13, 271.02) | 14.25 |
| Kiribati | 85.27 (59.2, 114.14) | 100.72 (74.42, 131.21) | 16.65 |
| Kuwait | 116.84 (88.5, 148.27) | 116.95 (92.22, 145.95) | 0.09 |
| Kyrgyzstan | 28.02 (20.33, 36.3) | 27.51 (19.19, 36.36) | -1.84 |
| Lao People's Democratic Republic | 157.38 (122.91, 187.96) | 195.79 (159.67, 233.93) | 21.84 |
| Latvia | 88.77 (63.01, 115.09) | 121.22 (87.97, 154.94) | 31.16 |
| Lebanon | 64.38 (44.71, 83.94) | 73.26 (54.73, 93.19) | 12.92 |
| Lesotho | 31.02 (21.1, 41.94) | 29.49 (19.96, 40.35) | -5.06 |
| Liberia | 110.51 (81.76, 137.69) | 191.2 (150.8, 242.4) | 54.82 |
| Libya | 150.53 (118.72, 179.59) | 320.3 (261.1, 383.96) | 75.51 |
| Lithuania | 182.4 (146.65, 215.23) | 166.42 (134.54, 198.24) | -9.17 |
| Luxembourg | 49.7 (36.84, 65.29) | 54.87 (39.12, 73.32) | 9.90 |
| Madagascar | 52.27 (37.3, 70.46) | 64.55 (44.68, 88.02) | 21.10 |
| Malawi | 33 (23.55, 42.38) | 32.92 (23.66, 43.09) | -0.24 |
| Malaysia | 28.07 (19.95, 36.85) | 35.48 (26.06, 44.62) | 23.43 |
| Maldives | 28.37 (19, 38.33) | 26.69 (17.8, 36.57) | -6.10 |
| Mali | 93.79 (71.15, 116.92) | 149.02 (119.24, 179.49) | 46.30 |
| Malta | 42.69 (31.37, 55.43) | 41.53 (30.12, 53.58) | -2.75 |
| Marshall Islands | 32.78 (21.71, 44.63) | 31.53 (20.19, 42.49) | -3.89 |
| Mauritania | 56.57 (37.29, 76.52) | 118.49 (98.69, 139.53) | 73.93 |
| Mauritius | 38.63 (26.67, 50.47) | 36.26 (25.2, 47.46) | -6.33 |
| Mexico | 45.79 (33.26, 59.35) | 42.63 (30.13, 56.6) | -7.15 |
| Micronesia | 63.49 (41.13, 86.26) | 67.65 (44.61, 91.46) | 6.35 |
| Monaco | 86.07 (57.95, 114.99) | 96.14 (70.31, 123) | 11.06 |
| Mongolia | 39.47 (27.08, 52.67) | 41.38 (27.98, 54.68) | 4.73 |
| Montenegro | 114.18 (83.23, 145.34) | 131.52 (100.41, 165.48) | 14.14 |
| Morocco | 33.38 (24.14, 42.75) | 49.36 (35.45, 63.89) | 39.12 |
| Mozambique | 30.19 (21.99, 39.99) | 26.59 (18.47, 35.33) | -12.70 |
| Myanmar | 73.12 (54.14, 95.43) | 73.03 (54.12, 91.5) | -0.12 |
| Namibia | 42.81 (31.04, 55.12) | 40.43 (28.25, 53.91) | -5.72 |
| Nauru | 51.54 (37.62, 66.98) | 51.34 (37.31, 66.35) | -0.39 |
| Nepal | 53.15 (41.22, 65.73) | 90.96 (74.27, 108.6) | 53.73 |
| Netherlands | 73.07 (53.1, 91.94) | 98.89 (77.77, 120.33) | 30.26 |
| New Zealand | 47.74 (29.98, 64.91) | 41.77 (27.39, 56.04) | -13.36 |
| Nicaragua | 28.87 (19.8, 39.91) | 28.53 (19.33, 39.18) | -1.18 |
| Niger | 24.87 (17.36, 32.61) | 23.67 (16.58, 30.72) | -4.95 |
| Nigeria | 42.81 (31.02, 54.94) | 38.61 (27.61, 49.65) | -10.33 |
| Niue | 56.2 (39.73, 74.06) | 64.41 (47.48, 80.66) | 13.64 |
| North Macedonia | 40.19 (28.97, 53.14) | 33.4 (22.94, 45.43) | -18.51 |
| Northern Mariana Islands | 230.88 (185.9, 276.96) | 362.75 (301.66, 429.92) | 45.18 |
| Norway | 77.74 (55.51, 104.59) | 74.64 (52.01, 97.51) | -4.07 |
| Oman | 61.72 (46.37, 77.69) | 56.3 (42.84, 69.7) | -9.19 |
| Pakistan | 35.61 (23.68, 47.05) | 36.84 (25.04, 49.07) | 3.40 |
| Palau | 79.74 (52.75, 110.9) | 72.01 (47.32, 99.24) | -10.20 |
| Palestine | 43.08 (28.27, 59.8) | 42.86 (28.38, 58.61) | -0.51 |
| Panama | 32.88 (22.46, 44.14) | 32.21 (22.37, 42.91) | -2.06 |
| Papua New Guinea | 45.56 (29.56, 63.88) | 41.85 (27.34, 58.38) | -8.49 |
| Paraguay | 41.86 (28.13, 56.67) | 45.79 (32.53, 61.89) | 8.97 |
| Peru | 25.57 (18.05, 32.78) | 24.27 (17.56, 31.16) | -5.22 |
| Philippines | 46.22 (38.08, 54.3) | 49.52 (42.04, 57.18) | 6.90 |
| Poland | 78.37 (61.21, 95.83) | 79.78 (62.81, 97.41) | 1.78 |
| Portugal | 96.04 (69.65, 123.67) | 80.28 (63.65, 98.28) | -17.92 |
| Puerto Rico | 76.51 (53.92, 102.53) | 72.46 (49.79, 98.54) | -5.44 |
| Qatar | 47.98 (33.58, 63.59) | 49.59 (34.03, 66.44) | 3.30 |
| Republic of Korea | 114.94 (83.25, 148.15) | 110.17 (82.87, 138.26) | -4.24 |
| Republic of Moldova | 37.86 (27.4, 50.69) | 44.41 (31.19, 58.98) | 15.96 |
| Romania | 61.76 (48.39, 75.9) | 61.76 (50.48, 73.02) | 0.00 |
| Russian Federation | 286.38 (220.54, 347.56) | 347.64 (291.87, 406.55) | 19.38 |
| Rwanda | 59.98 (44.41, 79.91) | 62.86 (42.83, 86.8) | 4.69 |
| Saint Kitts and Nevis | 42.37 (26.94, 59.06) | 40.54 (27.67, 55.22) | -4.42 |
| Saint Lucia | 42.75 (28.02, 59.13) | 40.21 (26.02, 54.46) | -6.13 |
| Saint Vincent and the Grenadines | 41.45 (26.45, 56.83) | 38.81 (25.83, 52.87) | -6.58 |
| Samoa | 39.14 (28.2, 50.37) | 35.99 (25.72, 46.98) | -8.39 |
| San Marino | 64.63 (43.41, 88.39) | 66.69 (42.96, 89.1) | 3.14 |
| Sao Tome and Principe | 37.19 (26.09, 50.02) | 41.98 (31.14, 54.56) | 12.12 |
| Saudi Arabia | 78.25 (51.23, 104.7) | 80.65 (54.86, 108.67) | 3.02 |
| Senegal | 33.26 (22.54, 44.47) | 29.82 (19.69, 40.42) | -10.92 |
| Serbia | 55.68 (43.9, 68.55) | 72.73 (56.25, 89.57) | 26.71 |
| Seychelles | 40.25 (29.79, 51.18) | 48.95 (37.96, 60.41) | 19.57 |
| Sierra Leone | 33.72 (22.3, 45.25) | 30.33 (19.51, 40.57) | -10.60 |
| Singapore | 46.73 (30.81, 62.68) | 46.24 (31.65, 61.23) | -1.05 |
| Slovakia | 65.17 (47.71, 82.38) | 59.83 (43.95, 76.06) | -8.55 |
| Slovenia | 62.56 (50.11, 74.93) | 128.82 (101.7, 157.46) | 72.23 |
| Solomon Islands | 38.33 (27.65, 51.4) | 37.59 (26.48, 48.38) | -1.95 |
| Somalia | 51.04 (34.18, 76.89) | 51.8 (32.76, 78.28) | 1.48 |
| South Africa | 136.51 (105.89, 163.76) | 92.35 (75.89, 108.25) | -39.08 |
| South Sudan | 50.77 (35.8, 68.42) | 52.37 (36.52, 72.83) | 3.10 |
| Spain | 159.9 (133.41, 187.43) | 109.95 (88.25, 132.7) | -37.45 |
| Sri Lanka | 76.1 (61.59, 90.87) | 47.05 (36.25, 59.33) | -48.08 |
| Sudan | 99.96 (74.18, 126.19) | 126.49 (95.08, 159.04) | 23.54 |
| Suriname | 39.33 (26.57, 53.89) | 40.32 (27.45, 54.61) | 2.49 |
| Sweden | 72.41 (61.19, 84.6) | 312.73 (254.74, 372.61) | 146.30 |
| Switzerland | 458 (376.18, 536.75) | 175.44 (148.05, 205.63) | -95.96 |
| Syrian Arab Republic | 96.93 (73.58, 121.93) | 115.38 (88.33, 143.16) | 17.42 |
| Taiwan (Province of China) | 81.87 (68.95, 94.32) | 75.36 (59.29, 91.56) | -8.29 |
| Tajikistan | 81.68 (56.08, 109.77) | 73.4 (50.88, 96.07) | -10.69 |
| Thailand | 38.93 (26.04, 51.14) | 41.89 (29.02, 55.74) | 7.33 |
| Timor-Leste | 29.13 (20.62, 38.43) | 28.95 (20.62, 38.26) | -0.62 |
| Togo | 26.65 (18.02, 36.06) | 28.3 (18.54, 38.47) | 6.01 |
| Tokelau | 41.14 (30.43, 51.96) | 38.42 (27.42, 49.84) | -6.84 |
| Tonga | 37.06 (26.64, 47.6) | 36.61 (25.25, 47.8) | -1.22 |
| Trinidad and Tobago | 42.65 (27.02, 59.28) | 42.32 (28.62, 55.61) | -0.78 |
| Tunisia | 103.13 (76.99, 132.28) | 129.28 (98.86, 159.9) | 22.60 |
| Türkiye | 91.82 (63.35, 121.7) | 91.41 (65.55, 119.07) | -0.45 |
| Turkmenistan | 91.96 (64.09, 120.94) | 143.59 (109.31, 180.56) | 44.56 |
| Tuvalu | 40.61 (29.15, 52.44) | 39.17 (28.29, 51) | -3.61 |
| Uganda | 50.18 (34.38, 68.56) | 61.53 (44.44, 84.16) | 20.39 |
| Ukraine | 179.27 (144.2, 215.81) | 228.75 (179.2, 285.13) | 24.37 |
| United Arab Emirates | 112.92 (83.89, 144.78) | 176.28 (129.29, 224.28) | 44.54 |
| United Kingdom | 124.98 (99.16, 149.53) | 372.46 (317.54, 424.08) | 109.20 |
| United Republic of Tanzania | 65.87 (45.6, 90.71) | 78.17 (55.16, 105.8) | 17.12 |
| United States of America | 205.39 (164.26, 246.19) | 1594.63 (1308.06, 1849.82) | 204.95 |
| United States Virgin Islands | 70.59 (53.26, 89.16) | 66.18 (47.23, 93.94) | -6.45 |
| Uruguay | 60.17 (40.05, 81.38) | 57.92 (42.19, 74.27) | -3.81 |
| Uzbekistan | 78.66 (53.4, 103.75) | 77.64 (55.81, 100.41) | -1.31 |
| Vanuatu | 35.83 (26.3, 47.26) | 34.62 (25.02, 45.14) | -3.44 |
| Venezuela | 48.36 (31.38, 66.45) | 42.63 (27.57, 58.47) | -12.61 |
| Vietnam | 63.02 (50, 79.87) | 87.91 (66.65, 112.13) | 33.29 |
| Yemen | 84.21 (61.09, 110.02) | 105.83 (79.4, 136.01) | 22.85 |
| Zambia | 64.79 (47.91, 87.45) | 74.6 (52.82, 101.86) | 14.10 |
| Zimbabwe | 83.82 (60.94, 107.52) | 82.25 (62.71, 101.99) | -1.89 |
