# Supplementary material for: Global burden of disease due to opioid, amphetamine, cocaine, and cannabis use disorders, 1990-2021: a systematic analysis for the Global Burden of Disease Study 2021
Source: PLoS One. 2025 Aug 21;20(8):e0328276. doi: 10.1371/journal.pone.0328276 (PMC12370144; doi:10.1371/journal.pone.0328276)
Supplement: S9 Table — (DOCX) [file pone.0328276.s010.docx]

**S9 Table. Age-standardized disability-adjusted life year (DALY) rates per 100,000 attributable to any amphetamine use disorder, stratified by country in 1990 and 2021, and total percentage change**

| **Location** | **DALY rate (95% UI) in 1990** | **DALY rate (95% UI) in 2021** | **% Change** |
| --- | --- | --- | --- |
| Afghanistan | 6.76 (3.93, 11.06) | 6.58 (4.17, 9.91) | -2.70 |
| Albania | 15.5 (8.69, 24.37) | 19.55 (11.8, 30.07) | 23.21 |
| Algeria | 5.33 (3.07, 8.2) | 6.64 (4.24, 9.74) | 21.98 |
| American Samoa | 18.25 (10.08, 29.53) | 20.89 (11.95, 33.24) | 13.51 |
| Andorra | 17.84 (9.48, 28.23) | 17.35 (9.36, 27.11) | -2.79 |
| Angola | 5.16 (2.75, 8.33) | 5.45 (2.98, 8.82) | 5.47 |
| Antigua and Barbuda | 6.62 (3.6, 10.64) | 8.33 (4.97, 13.01) | 22.98 |
| Argentina | 8.1 (4.55, 13.25) | 8.57 (4.81, 13.87) | 5.64 |
| Armenia | 15.21 (8.29, 24.69) | 15.24 (8.69, 23.88) | 0.20 |
| Australia | 66.95 (39.38, 104.87) | 83.53 (55.24, 120.31) | 22.13 |
| Austria | 17.09 (9.78, 26.81) | 23.18 (14.28, 35.24) | 30.48 |
| Azerbaijan | 15.23 (8.3, 24.01) | 15.58 (8.64, 24.54) | 2.27 |
| Bahamas | 6.5 (3.5, 10.37) | 7.96 (4.7, 12.24) | 20.26 |
| Bahrain | 4.75 (2.4, 7.69) | 5.2 (2.91, 8.05) | 9.05 |
| Bangladesh | 1.77 (1.07, 2.76) | 1.76 (1.11, 2.73) | -0.57 |
| Barbados | 7.01 (3.7, 11.61) | 7.81 (4.45, 12.36) | 10.81 |
| Belarus | 23.01 (13.03, 35.43) | 25.01 (15.9, 36.6) | 8.33 |
| Belgium | 14.69 (8.24, 22.75) | 22.08 (15.09, 31.52) | 40.75 |
| Belize | 6.38 (3.21, 10.52) | 6.92 (3.91, 10.98) | 8.12 |
| Benin | 4.65 (2.39, 7.92) | 4.6 (2.32, 7.46) | -1.08 |
| Bermuda | 8.02 (4.66, 12.51) | 13.39 (9.04, 18.59) | 51.26 |
| Bhutan | 1.68 (0.99, 2.68) | 1.68 (0.98, 2.6) | 0.00 |
| Bolivia | 9.02 (4.92, 13.96) | 10.12 (5.8, 15.83) | 11.51 |
| Bosnia and Herzegovina | 8.67 (4.87, 13.85) | 9.18 (5.03, 14.62) | 5.72 |
| Botswana | 7.92 (4.5, 12.53) | 8.25 (4.58, 13.06) | 4.08 |
| Brazil | 24.24 (13.62, 37.84) | 23.44 (13.44, 36.64) | -3.36 |
| Brunei Darussalam | 17.23 (9.65, 26.99) | 17.06 (10.09, 27.16) | -0.99 |
| Bulgaria | 21.01 (11.79, 33.36) | 26.54 (16.12, 41.69) | 23.37 |
| Burkina Faso | 4.65 (2.44, 7.57) | 4.64 (2.33, 7.64) | -0.22 |
| Burundi | 5.14 (2.87, 8.39) | 5.06 (2.83, 8.18) | -1.57 |
| Cabo Verde | 4.7 (2.4, 7.81) | 4.8 (2.49, 8.15) | 2.11 |
| Cambodia | 23.86 (12.68, 39.55) | 24.28 (12.77, 39.06) | 1.74 |
| Cameroon | 4.69 (2.44, 7.56) | 4.73 (2.4, 8.02) | 0.85 |
| Canada | 26.16 (14.97, 41.64) | 50.32 (39.5, 62.87) | 65.42 |
| Central African Republic | 5.07 (2.83, 8.19) | 5.12 (2.79, 8.09) | 0.98 |
| Chad | 4.73 (2.32, 7.72) | 4.57 (2.35, 7.33) | -3.44 |
| Chile | 12.2 (6.78, 19.94) | 13.53 (7.92, 21.31) | 10.35 |
| China | 74.37 (51.2, 105.73) | 44.4 (29.4, 65.07) | -51.58 |
| Colombia | 6.5 (3.72, 10.16) | 6.64 (4.13, 10.1) | 2.13 |
| Comoros | 5.14 (2.74, 8.36) | 5.64 (3.16, 9) | 9.28 |
| Congo | 5.38 (3, 8.73) | 6.1 (3.48, 9.38) | 12.56 |
| Cook Islands | 18.5 (10.17, 30.48) | 19.6 (11.38, 30.94) | 5.78 |
| Costa Rica | 5.71 (3.07, 9.29) | 7.62 (5.01, 10.89) | 28.86 |
| Côte d'Ivoire | 16.67 (9.62, 26) | 18.86 (11.79, 28.07) | 12.34 |
| Croatia | 6.56 (3.64, 10.62) | 6.78 (3.85, 10.62) | 3.30 |
| Cuba | 17.81 (10.41, 27.75) | 20.29 (12.24, 30.45) | 13.04 |
| Cyprus | 28.42 (15.72, 45.07) | 34.53 (20.54, 53.35) | 19.47 |
| Czechia | 4.74 (2.51, 7.58) | 4.7 (2.55, 7.7) | -0.85 |
| Democratic People's Republic of Korea | 39.69 (24.32, 60.44) | 40.59 (24.48, 62.06) | 2.24 |
| Republic of the Congo | 5.04 (2.72, 8.22) | 5.3 (2.86, 8.44) | 5.03 |
| Denmark | 26.19 (16.93, 39.08) | 38.32 (28.16, 51.35) | 38.06 |
| Djibouti | 5.18 (2.74, 8.46) | 5.64 (3.14, 9.02) | 8.51 |
| Dominica | 6.65 (3.59, 11.14) | 8.14 (4.91, 12.64) | 20.22 |
| Dominican Republic | 6.15 (3.2, 9.66) | 7.02 (3.86, 11.15) | 13.23 |
| Ecuador | 8.9 (4.77, 14.38) | 11.25 (7.11, 16.66) | 23.43 |
| Egypt | 4.35 (2.28, 7.11) | 4.66 (2.41, 7.6) | 6.88 |
| El Salvador | 5.72 (3.2, 8.95) | 6.89 (4.21, 10.23) | 18.61 |
| Equatorial Guinea | 5.02 (2.69, 8.2) | 7.18 (4.36, 11.17) | 35.79 |
| Eritrea | 4.98 (2.74, 8.19) | 5.56 (3.13, 8.83) | 11.02 |
| Estonia | 29.88 (19.1, 45.4) | 62.02 (44.74, 84.34) | 73.03 |
| Eswatini | 7.91 (4.49, 12.97) | 8.51 (4.84, 13.38) | 7.31 |
| Ethiopia | 4.99 (2.85, 7.89) | 4.86 (2.76, 7.55) | -2.64 |
| Fiji | 18.41 (10.03, 30.93) | 18.23 (9.99, 29.46) | -0.98 |
| Finland | 33.56 (19.84, 51.83) | 60.3 (43.66, 81.77) | 58.60 |
| France | 10.07 (6.08, 15.38) | 16.24 (11.59, 22.04) | 47.79 |
| Gabon | 5.39 (2.85, 8.79) | 6.72 (4.03, 10.13) | 22.05 |
| Gambia | 4.7 (2.56, 7.74) | 4.68 (2.45, 7.33) | -0.43 |
| Georgia | 15.56 (8.88, 25.03) | 17 (10.16, 25.32) | 8.85 |
| Germany | 18.88 (10.82, 31.07) | 26.13 (17, 38.39) | 32.50 |
| Ghana | 4.81 (2.54, 7.85) | 4.65 (2.41, 7.44) | -3.38 |
| Greece | 5.62 (3.27, 8.6) | 9.13 (6.15, 12.86) | 48.52 |
| Greenland | 26.61 (15.17, 41.11) | 29.79 (19.83, 42.37) | 11.29 |
| Grenada | 6.68 (3.61, 10.75) | 10.01 (6.53, 14.42) | 40.45 |
| Guam | 19.73 (10.86, 32.24) | 21.18 (11.85, 33.45) | 7.09 |
| Guatemala | 8 (4.81, 12.73) | 13.36 (9.78, 17.36) | 51.28 |
| Guinea | 4.68 (2.39, 7.75) | 4.58 (2.41, 7.4) | -2.16 |
| Guinea-Bissau | 4.64 (2.45, 7.79) | 4.64 (2.38, 7.67) | 0.00 |
| Guyana | 5.63 (2.86, 9.41) | 6.96 (3.77, 10.8) | 21.21 |
| Haiti | 6.42 (3.59, 10.14) | 7.12 (3.82, 11.2) | 10.35 |
| Honduras | 6.33 (3.64, 10.08) | 7.66 (4.91, 11.76) | 19.07 |
| Hungary | 28.15 (15.5, 45.17) | 33.84 (19.74, 52.58) | 18.41 |
| Iceland | 24.51 (15.82, 36.08) | 48.43 (35.74, 64.89) | 68.10 |
| India | 2.08 (1.38, 3.19) | 2.5 (1.73, 3.51) | 18.39 |
| Indonesia | 22.46 (12.54, 35.64) | 22.48 (12.82, 35.8) | 0.09 |
| Iran | 11.61 (7.9, 16.79) | 16.28 (11.1, 21.63) | 33.81 |
| Iraq | 4.42 (2.36, 7.51) | 4.92 (2.68, 7.63) | 10.72 |
| Ireland | 13.79 (8.31, 20.76) | 34.11 (25.67, 45.32) | 90.56 |
| Israel | 18.57 (10.35, 29.14) | 22.35 (14.23, 32.91) | 18.53 |
| Italy | 33.68 (19.76, 51.78) | 36.03 (21.23, 54.02) | 6.74 |
| Jamaica | 6.27 (3.34, 10.03) | 6.97 (3.94, 10.77) | 10.58 |
| Japan | 14.93 (8.59, 23.75) | 14.9 (8.54, 23.11) | -0.20 |
| Jordan | 4.61 (2.48, 7.41) | 4.55 (2.58, 7.29) | -1.31 |
| Kazakhstan | 17.49 (10.74, 26.78) | 31.57 (21.47, 43.66) | 59.06 |
| Kenya | 4.9 (2.84, 7.83) | 5.22 (3.13, 7.83) | 6.33 |
| Kiribati | 24.63 (16.19, 36.57) | 34.48 (21.02, 47.94) | 33.64 |
| Kuwait | 5.73 (3.31, 8.55) | 8.1 (5.16, 11.34) | 34.61 |
| Kyrgyzstan | 18.33 (10.82, 27.67) | 21.8 (14.57, 31.72) | 17.34 |
| Lao People's Democratic Republic | 19.13 (10.1, 31.27) | 18.87 (10.44, 30.89) | -1.37 |
| Latvia | 33.13 (19.21, 51.84) | 42.93 (27.47, 64.62) | 25.91 |
| Lebanon | 7.13 (3.92, 11.8) | 8.52 (5.03, 12.95) | 17.81 |
| Lesotho | 7.68 (4.29, 12.32) | 8.27 (4.69, 13.14) | 7.40 |
| Liberia | 4.61 (2.47, 7.68) | 4.67 (2.44, 7.38) | 1.29 |
| Libya | 5.8 (3.61, 9.07) | 9.79 (6.03, 14.48) | 52.35 |
| Lithuania | 15.56 (9.49, 23.39) | 27.72 (19.51, 37.69) | 57.75 |
| Luxembourg | 25.17 (15.82, 38.05) | 31.34 (21.12, 45.09) | 21.92 |
| Madagascar | 5.16 (2.84, 8.39) | 5.31 (2.96, 8.64) | 2.87 |
| Malawi | 5.05 (2.74, 8.03) | 5.35 (2.94, 8.62) | 5.77 |
| Malaysia | 24.61 (13.19, 38.9) | 25.09 (13.44, 40.47) | 1.93 |
| Maldives | 24.42 (13.02, 39.49) | 27.49 (15.35, 43.14) | 11.84 |
| Mali | 4.67 (2.45, 7.66) | 4.66 (2.41, 7.56) | -0.21 |
| Malta | 19.42 (11.12, 30.29) | 32.57 (22.66, 46.35) | 51.71 |
| Marshall Islands | 18.36 (10.24, 29.44) | 18.82 (10.61, 30.48) | 2.47 |
| Mauritania | 4.8 (2.46, 7.92) | 4.7 (2.39, 7.69) | -2.11 |
| Mauritius | 24.3 (12.93, 39.68) | 31.97 (19.82, 48.01) | 27.43 |
| Mexico | 8.53 (5.16, 12.91) | 10.12 (6.51, 14.43) | 17.09 |
| Micronesia | 18.41 (10.01, 29.56) | 19.01 (10.71, 29.72) | 3.21 |
| Monaco | 17.74 (9.67, 28.2) | 17.17 (9.41, 27.44) | -3.27 |
| Mongolia | 15.49 (8.83, 24.38) | 16.64 (10.01, 26.24) | 7.16 |
| Montenegro | 15.01 (8.63, 24.87) | 15.36 (8.67, 24.14) | 2.31 |
| Morocco | 4.99 (2.93, 7.74) | 5.96 (3.85, 8.94) | 17.76 |
| Mozambique | 4.85 (2.57, 7.6) | 5.13 (2.73, 8.1) | 5.61 |
| Myanmar | 23.52 (12.31, 38.11) | 23.26 (12.31, 37.8) | -1.11 |
| Namibia | 7.95 (4.4, 12.54) | 8.21 (4.76, 13.08) | 3.22 |
| Nauru | 18.83 (10.56, 29.43) | 19.26 (11.46, 30.44) | 2.26 |
| Nepal | 1.74 (1.1, 2.74) | 1.7 (1.07, 2.57) | -2.33 |
| Netherlands | 19.69 (12.33, 30.95) | 24.83 (14.73, 37.27) | 23.19 |
| New Zealand | 58.43 (32.76, 90.72) | 72.33 (44.09, 106.2) | 21.34 |
| Nicaragua | 5.55 (3, 8.85) | 5.9 (3.38, 9.37) | 6.12 |
| Niger | 4.71 (2.54, 7.98) | 4.65 (2.35, 7.84) | -1.28 |
| Nigeria | 4.62 (2.62, 7.41) | 4.44 (2.5, 7.11) | -3.97 |
| Niue | 19.01 (10.89, 29.87) | 19.84 (11.8, 31.39) | 4.27 |
| North Macedonia | 15.18 (8.47, 23.98) | 17 (9.95, 26.09) | 11.32 |
| Northern Mariana Islands | 25.83 (15.23, 39.84) | 36.29 (22.51, 54.79) | 34.00 |
| Norway | 14.75 (8.65, 22.54) | 35.96 (26.62, 48.26) | 89.12 |
| Oman | 4.85 (2.56, 7.83) | 5.27 (2.96, 8.13) | 8.31 |
| Pakistan | 1.8 (1.13, 2.82) | 2.17 (1.44, 3.13) | 18.69 |
| Palau | 18.6 (9.82, 30.06) | 19.59 (10.8, 31.33) | 5.19 |
| Palestine | 4.21 (2.16, 7.13) | 4.08 (2.12, 7) | -3.14 |
| Panama | 5.92 (3.49, 9.37) | 7.15 (4.49, 10.56) | 18.88 |
| Papua New Guinea | 18.02 (9.79, 29.12) | 18.12 (9.73, 29.69) | 0.55 |
| Paraguay | 16.69 (8.74, 27.25) | 18.16 (10.01, 28.92) | 8.44 |
| Peru | 9.34 (5.13, 14.38) | 11.19 (6.75, 17.09) | 18.07 |
| Philippines | 23.69 (13.72, 37.04) | 22.95 (13.09, 35.7) | -3.17 |
| Poland | 34.51 (20.12, 54.06) | 40.31 (24.5, 60.65) | 15.54 |
| Portugal | 19.92 (11.1, 31.39) | 21.63 (12.94, 33.32) | 8.24 |
| Puerto Rico | 10.62 (6.08, 16.01) | 26.35 (19.68, 32.62) | 90.87 |
| Qatar | 5.14 (2.72, 8.25) | 5.28 (2.97, 8.25) | 2.69 |
| Republic of Korea | 15.62 (8.22, 25.16) | 15.75 (8.53, 25.27) | 0.83 |
| Republic of Moldova | 21.43 (12.11, 34.65) | 22.71 (13.46, 34.98) | 5.80 |
| Romania | 6.08 (3.74, 9.17) | 7.7 (5.07, 11.35) | 23.62 |
| Russian Federation | 32.02 (20.49, 46.99) | 44.79 (33.03, 60.01) | 33.56 |
| Rwanda | 5.26 (2.87, 8.84) | 5.32 (3.07, 8.62) | 1.13 |
| Saint Kitts and Nevis | 7.29 (4.22, 11.21) | 7.96 (4.85, 12.28) | 8.79 |
| Saint Lucia | 6.29 (3.24, 10.41) | 7.39 (4.25, 11.5) | 16.12 |
| Saint Vincent and the Grenadines | 6.27 (3.1, 10.28) | 7.33 (4.35, 11.11) | 15.62 |
| Samoa | 18.77 (10.3, 31.05) | 18.9 (10.43, 30.13) | 0.69 |
| San Marino | 17.81 (9.75, 28.71) | 17.02 (9.53, 27.08) | -4.54 |
| Sao Tome and Principe | 4.81 (2.62, 7.95) | 5.35 (2.89, 8.23) | 10.64 |
| Saudi Arabia | 4.47 (2.22, 7.66) | 4.35 (2.25, 7.18) | -2.72 |
| Senegal | 4.65 (2.37, 7.54) | 4.69 (2.46, 7.77) | 0.86 |
| Serbia | 12.24 (7, 19.13) | 15.07 (9.4, 22.05) | 20.80 |
| Seychelles | 25.99 (14.28, 40.95) | 32.6 (20.38, 48.95) | 22.66 |
| Sierra Leone | 4.68 (2.5, 7.81) | 4.65 (2.4, 7.74) | -0.64 |
| Singapore | 15.78 (8.72, 25.23) | 15.56 (8.51, 24.97) | -1.40 |
| Slovakia | 19.87 (11.42, 32.38) | 19.86 (11.44, 31.09) | -0.05 |
| Slovenia | 12.92 (8.09, 19.72) | 18.73 (12.07, 27.14) | 37.14 |
| Solomon Islands | 18.1 (9.54, 28.82) | 18.17 (9.82, 29.54) | 0.39 |
| Somalia | 5.12 (2.75, 8.74) | 5.07 (2.68, 8.19) | -0.98 |
| South Africa | 22.69 (13.59, 34.01) | 21.71 (13.92, 30.95) | -4.42 |
| South Sudan | 5.05 (2.83, 8.33) | 5 (2.69, 7.93) | -1.00 |
| Spain | 27.79 (16.38, 43.47) | 28.47 (17.36, 42.33) | 2.42 |
| Sri Lanka | 27.19 (16.07, 43.23) | 26.83 (15.48, 42.17) | -1.33 |
| Sudan | 5.23 (3.04, 8.36) | 5.71 (3.52, 8.84) | 8.78 |
| Suriname | 6.81 (3.73, 10.43) | 11.7 (7.42, 17.04) | 54.12 |
| Sweden | 19.92 (12.67, 29.14) | 46.81 (34.18, 62.6) | 85.44 |
| Switzerland | 21.67 (13.13, 33.31) | 27.47 (18.11, 40.34) | 23.72 |
| Syrian Arab Republic | 5.21 (3.07, 8.26) | 5.96 (3.83, 8.61) | 13.45 |
| Taiwan (Province of China) | 43.81 (26.2, 68.84) | 64.63 (43.99, 93.32) | 38.88 |
| Tajikistan | 15.42 (8.6, 24.79) | 16.14 (9.05, 24.93) | 4.56 |
| Thailand | 46.72 (25.49, 74.71) | 50.1 (29.96, 78.34) | 6.98 |
| Timor-Leste | 23.91 (12.6, 38.74) | 24.14 (12.97, 39.73) | 0.96 |
| Togo | 4.72 (2.46, 7.91) | 4.67 (2.42, 7.8) | -1.06 |
| Tokelau | 18.5 (10.29, 29.44) | 19.3 (11.08, 29.89) | 4.23 |
| Tonga | 18.34 (9.8, 29.22) | 18.59 (10.35, 29.78) | 1.35 |
| Trinidad and Tobago | 6.32 (3.38, 10.13) | 9.02 (5.61, 13.52) | 35.57 |
| Tunisia | 5.08 (2.99, 8) | 6.63 (4.02, 9.64) | 26.63 |
| Türkiye | 15.67 (8.7, 25.15) | 26.87 (17.06, 44.27) | 53.93 |
| Turkmenistan | 18.23 (9.88, 30.59) | 19.14 (10.89, 30.85) | 4.87 |
| Tuvalu | 2.69 (1.53, 4.35) | 3.59 (2.25, 5.34) | 28.86 |
| Uganda | 4.89 (2.58, 8.26) | 5.31 (2.94, 8.42) | 8.24 |
| Ukraine | 21.17 (12.38, 32.54) | 26.7 (16.87, 39.36) | 23.21 |
| United Arab Emirates | 5.64 (2.94, 9.19) | 8.78 (5.43, 12.97) | 44.26 |
| United Kingdom | 39.28 (23.78, 58.69) | 59.56 (42.38, 82.96) | 41.63 |
| United Republic of Tanzania | 5.05 (2.68, 8.1) | 5.66 (3.29, 8.69) | 11.40 |
| United States of America | 11.58 (7.28, 16.62) | 18.58 (11.12, 26.54) | 47.28 |
| United States Virgin Islands | 33.38 (19.24, 51.71) | 103.91 (83.99, 129.92) | 113.56 |
| Uruguay | 9.33 (5, 15.29) | 11.05 (6.57, 16.95) | 16.92 |
| Uzbekistan | 15.39 (8.59, 25.45) | 16.5 (9.6, 25.19) | 6.96 |
| Vanuatu | 18.14 (9.52, 28) | 18.23 (10.18, 29.32) | 0.49 |
| Venezuela | 5.59 (3.08, 9.06) | 5.71 (3.1, 8.89) | 2.12 |
| Vietnam | 26.29 (15.1, 40.66) | 35.04 (22.24, 52.78) | 28.73 |
| Yemen | 4.92 (2.85, 7.91) | 5.11 (3.07, 8.07) | 3.79 |
| Zambia | 5.22 (2.89, 8.68) | 6.03 (3.51, 9.29) | 14.42 |
| Zimbabwe | 4.55 (2.67, 7.4) | 4.75 (2.81, 6.94) | 4.30 |
