# Supplementary material for: Global burden of disease due to opioid, amphetamine, cocaine, and cannabis use disorders, 1990-2021: a systematic analysis for the Global Burden of Disease Study 2021
Source: PLoS One. 2025 Aug 21;20(8):e0328276. doi: 10.1371/journal.pone.0328276 (PMC12370144; doi:10.1371/journal.pone.0328276)
Supplement: S10 Table — (DOCX) [file pone.0328276.s011.docx]

**S10 Table. Age-standardized disability-adjusted life year (DALY) rates per 100,000 attributable to any cocaine use disorder, stratified by country in 1990 and 2021, and total percentage change**

| **Location** | **DALY rate (95% UI) in 1990** | **DALY rate (95% UI) in 2021** | **% Change** |
| --- | --- | --- | --- |
| Afghanistan | 15.96 (7.75, 30.65) | 10.85 (5.17, 22.26) | -38.59 |
| Albania | 6.99 (4.47, 10.45) | 6.48 (3.83, 10.16) | -7.58 |
| Algeria | 6.91 (3.78, 11.87) | 7.49 (4.91, 10.61) | 8.06 |
| American Samoa | 1.32 (0.96, 1.78) | 1.79 (1.28, 3.22) | 30.46 |
| Andorra | 16.13 (9.19, 25.71) | 17.91 (10.4, 28.12) | 10.47 |
| Angola | 2.02 (1.24, 3.35) | 2.36 (1.58, 3.67) | 15.56 |
| Antigua and Barbuda | 16.1 (10.29, 24.78) | 20.9 (14.58, 29.36) | 26.09 |
| Argentina | 34.94 (20.27, 55.71) | 36.74 (22.11, 56.94) | 5.02 |
| Armenia | 3.44 (2.2, 5.42) | 3.54 (2.34, 5.4) | 2.87 |
| Australia | 31.71 (19.13, 50.64) | 35.87 (21.69, 56.12) | 12.33 |
| Austria | 21.29 (12.75, 33.98) | 24.08 (14.87, 37.85) | 12.31 |
| Azerbaijan | 3.05 (1.68, 5.11) | 3.33 (2, 5.22) | 8.78 |
| Bahamas | 15.21 (9.51, 23.44) | 16.21 (10.66, 23.78) | 6.37 |
| Bahrain | 2.86 (1.97, 4.23) | 3.1 (2.11, 4.38) | 8.06 |
| Bangladesh | 2.12 (0.98, 3.95) | 1.94 (1.04, 3.1) | -8.87 |
| Barbados | 7.34 (4.39, 11.53) | 8.98 (5.92, 13.07) | 20.17 |
| Belarus | 8.27 (5.56, 12.13) | 9.29 (6.56, 12.98) | 11.63 |
| Belgium | 16.84 (10.33, 26.11) | 22.79 (15.3, 32.93) | 30.26 |
| Belize | 13.08 (7.43, 21.64) | 15.23 (9.84, 23.34) | 15.22 |
| Benin | 1.08 (0.74, 1.53) | 1.05 (0.71, 1.5) | -2.82 |
| Bermuda | 25.38 (18.71, 34.07) | 33.24 (25.04, 43.69) | 26.98 |
| Bhutan | 2.14 (0.94, 4.02) | 1.83 (0.97, 3.05) | -15.65 |
| Bolivia | 23.68 (17.08, 32.72) | 27.81 (19.58, 38.59) | 16.08 |
| Bosnia and Herzegovina | 1.89 (1.17, 2.94) | 2.12 (1.35, 3.16) | 11.48 |
| Botswana | 3.8 (2.36, 5.57) | 2.61 (1.82, 3.49) | -37.57 |
| Brazil | 21.61 (14.03, 32.41) | 50.52 (39.46, 64.93) | 84.92 |
| Brunei Darussalam | 24.57 (15.12, 37.72) | 21.06 (13.22, 31.64) | -15.42 |
| Bulgaria | 11.06 (7.15, 16.59) | 10.38 (6.24, 16.57) | -6.35 |
| Burkina Faso | 1.1 (0.75, 1.59) | 1.08 (0.7, 1.53) | -1.83 |
| Burundi | 2.55 (1.33, 5.14) | 2.03 (1.12, 4.91) | -22.81 |
| Cabo Verde | 0.83 (0.53, 1.28) | 1.21 (0.77, 1.78) | 37.69 |
| Cambodia | 0.75 (0.43, 1.27) | 0.63 (0.4, 0.99) | -17.44 |
| Cameroon | 1.19 (0.81, 1.69) | 1.44 (0.92, 2.11) | 19.07 |
| Canada | 45.96 (30.08, 71.44) | 84.87 (68.31, 105.13) | 61.33 |
| Central African Republic | 1.9 (1.18, 3.5) | 1.97 (1.21, 3.58) | 3.62 |
| Chad | 1.02 (0.69, 1.47) | 1.03 (0.7, 1.47) | 0.98 |
| Chile | 25.76 (15.85, 40.53) | 31.05 (18.5, 46.22) | 18.68 |
| China | 2.89 (1.96, 4.1) | 1.53 (1, 2.15) | -63.60 |
| Colombia | 28.71 (20.52, 39.49) | 24.61 (16.64, 35.24) | -15.41 |
| Comoros | 2.77 (1.35, 5.27) | 2.97 (1.71, 5.81) | 6.97 |
| Congo | 2.51 (1.62, 4.48) | 2.9 (1.83, 4.51) | 14.44 |
| Cook Islands | 0.82 (0.52, 1.24) | 1.11 (0.7, 1.65) | 30.28 |
| Costa Rica | 17.6 (13.1, 24.2) | 20.5 (15.55, 26.99) | 15.25 |
| Côte d'Ivoire | 7.95 (5.63, 10.91) | 5.7 (3.63, 8.95) | -33.27 |
| Croatia | 17.01 (11.36, 24.82) | 13.21 (8.1, 20.7) | -25.28 |
| Cuba | 18.93 (11.64, 29.22) | 19.52 (12.2, 29.35) | 3.07 |
| Cyprus | 3.28 (2.71, 4.08) | 2.02 (1.46, 2.81) | -48.47 |
| Czechia | 1.09 (0.75, 1.55) | 1.15 (0.77, 1.65) | 5.36 |
| Democratic People's Republic of Korea | 1.47 (0.95, 2.26) | 1.27 (0.82, 1.86) | -14.62 |
| Republic of the Congo | 1.89 (1.19, 3.14) | 2.15 (1.36, 3.71) | 12.89 |
| Denmark | 30.75 (22.46, 43.18) | 31.83 (22.66, 44.27) | 3.45 |
| Djibouti | 2.27 (1.26, 4.68) | 2.62 (1.51, 6.25) | 14.34 |
| Dominica | 21.82 (14.19, 32.74) | 38.93 (28.2, 53.5) | 57.89 |
| Dominican Republic | 13.51 (8.32, 21.56) | 13.11 (7.82, 20.52) | -3.01 |
| Ecuador | 12.51 (9.32, 16.84) | 22.01 (16.9, 28.94) | 56.50 |
| Egypt | 1.86 (1.09, 3.05) | 1.82 (1.08, 2.88) | -2.17 |
| El Salvador | 21.97 (17.2, 27.13) | 18.91 (13.97, 24.15) | -15.00 |
| Equatorial Guinea | 2.12 (1.3, 3.62) | 3.04 (1.8, 4.56) | 36.04 |
| Eritrea | 2.63 (1.34, 5.64) | 3.25 (1.72, 7.38) | 21.17 |
| Estonia | 21.66 (15.94, 28.43) | 19.6 (13.84, 27.73) | -9.99 |
| Eswatini | 6.51 (4.43, 9.43) | 6.09 (4.18, 8.85) | -6.67 |
| Ethiopia | 2.85 (1.42, 4.91) | 1.77 (1.2, 2.61) | -47.63 |
| Fiji | 1.21 (0.87, 1.68) | 0.81 (0.58, 1.17) | -40.13 |
| Finland | 17.55 (13.66, 22.44) | 22.92 (18.79, 28.66) | 26.70 |
| France | 9.09 (5.76, 14.05) | 10.96 (7.51, 16.23) | 18.71 |
| Gabon | 2.54 (1.65, 4.43) | 3 (1.86, 4.7) | 16.64 |
| Gambia | 1.08 (0.74, 1.53) | 1.1 (0.71, 1.58) | 1.83 |
| Georgia | 5.5 (3.4, 7.69) | 5.42 (3.87, 7.61) | -1.47 |
| Germany | 21.95 (13.67, 33.93) | 23.55 (15.15, 34.55) | 7.04 |
| Ghana | 1.41 (1, 2.03) | 1.63 (1.09, 2.35) | 14.50 |
| Greece | 9.33 (5.57, 15.44) | 11.8 (7.46, 17.67) | 23.49 |
| Greenland | 48.98 (31.92, 75.22) | 44.73 (30.41, 64.42) | -9.08 |
| Grenada | 20.67 (13.87, 30.29) | 42.65 (31.92, 56.66) | 72.43 |
| Guam | 2.7 (1.95, 3.57) | 0.85 (0.6, 1.25) | -115.58 |
| Guatemala | 34.16 (30.03, 38.52) | 42.19 (35.16, 50.25) | 21.11 |
| Guinea | 1.05 (0.71, 1.53) | 1.06 (0.72, 1.53) | 0.95 |
| Guinea-Bissau | 1.07 (0.72, 1.54) | 1.05 (0.69, 1.55) | -1.89 |
| Guyana | 12.02 (6.69, 20.65) | 15.09 (9.9, 22.51) | 22.75 |
| Haiti | 20.98 (14.09, 31.24) | 25.85 (17.61, 36.32) | 20.87 |
| Honduras | 29.77 (21.82, 38.24) | 32.26 (22.13, 45.46) | 8.03 |
| Hungary | 5.67 (3.66, 8.89) | 5.26 (3, 8.93) | -7.51 |
| Iceland | 26.39 (18.15, 38.59) | 41.43 (30.59, 55.89) | 45.10 |
| India | 2.9 (1.44, 5.06) | 3.03 (2.15, 4.1) | 4.39 |
| Indonesia | 0.47 (0.32, 0.71) | 0.58 (0.41, 0.86) | 21.03 |
| Iran | 26.04 (14.81, 45.74) | 19.02 (15.28, 24.08) | -31.41 |
| Iraq | 8.56 (6.28, 11.27) | 7.31 (5.21, 10.32) | -15.79 |
| Ireland | 23.6 (14.57, 37.09) | 30.07 (20.24, 42.41) | 24.23 |
| Israel | 16.06 (10.21, 24.06) | 17.21 (11.77, 24.99) | 6.92 |
| Italy | 14.75 (9.49, 21.82) | 14.4 (8.98, 21.94) | -2.40 |
| Jamaica | 23.58 (14.32, 36.17) | 22.03 (13.42, 33.97) | -6.80 |
| Japan | 13.4 (7.9, 21) | 13.45 (8.15, 20.89) | 0.37 |
| Jordan | 2.96 (2.11, 4.31) | 2.28 (1.46, 3.37) | -26.10 |
| Kazakhstan | 6.66 (4.69, 9.09) | 15.71 (12.14, 19.89) | 85.82 |
| Kenya | 1.62 (1.07, 2.32) | 1.93 (1.34, 2.85) | 17.51 |
| Kiribati | 16.65 (9.79, 24.13) | 28.38 (15.71, 44.42) | 53.33 |
| Kuwait | 4.23 (3.14, 5.84) | 4.68 (3.28, 7.12) | 10.11 |
| Kyrgyzstan | 11.3 (6.25, 14.81) | 10.29 (7.68, 13.28) | -9.36 |
| Lao People's Democratic Republic | 0.8 (0.44, 1.45) | 0.66 (0.43, 1.01) | -19.24 |
| Latvia | 15.61 (11.04, 18.93) | 8.35 (6.28, 11.26) | -62.57 |
| Lebanon | 2.91 (1.89, 4.56) | 2.86 (2.07, 3.77) | -1.73 |
| Lesotho | 5.53 (3.65, 8.48) | 5.97 (3.9, 8.77) | 7.66 |
| Liberia | 1.11 (0.75, 1.58) | 1.06 (0.72, 1.53) | -4.61 |
| Libya | 8.93 (5.78, 13.46) | 14.3 (8.85, 22.45) | 47.08 |
| Lithuania | 14.06 (10.54, 18.2) | 10.83 (7.77, 14.79) | -26.10 |
| Luxembourg | 21.81 (16.18, 29.11) | 21 (15.03, 29.75) | -3.78 |
| Madagascar | 2.59 (1.39, 4.62) | 2.42 (1.35, 4.87) | -6.79 |
| Malawi | 2.34 (1.19, 4.67) | 2.64 (1.38, 5.85) | 12.06 |
| Malaysia | 0.98 (0.59, 1.37) | 1.13 (0.79, 1.54) | 14.24 |
| Maldives | 0.97 (0.51, 1.51) | 2.3 (1.56, 3.45) | 86.34 |
| Mali | 1.05 (0.69, 1.49) | 0.98 (0.66, 1.44) | -6.90 |
| Malta | 9.82 (6.3, 14.71) | 13.97 (9.85, 19.58) | 35.25 |
| Marshall Islands | 1.15 (0.63, 1.95) | 1.08 (0.73, 1.66) | -6.28 |
| Mauritania | 1.07 (0.74, 1.51) | 1.12 (0.74, 1.62) | 4.57 |
| Mauritius | 0.39 (0.23, 0.66) | 8.69 (7.29, 10.28) | 310.38 |
| Mexico | 28.94 (20.52, 40.72) | 29.29 (21.55, 40.53) | 1.20 |
| Micronesia | 1.21 (0.68, 2.03) | 1.04 (0.66, 1.61) | -15.14 |
| Monaco | 15.04 (8.3, 24.04) | 17.02 (9.91, 26.93) | 12.37 |
| Mongolia | 3.76 (2.31, 5.69) | 5.26 (3.76, 7.44) | 33.57 |
| Montenegro | 4.48 (2.55, 7.35) | 4.43 (2.59, 7.16) | -1.12 |
| Morocco | 6.32 (3.2, 11.29) | 7.54 (4.52, 12.91) | 17.65 |
| Mozambique | 1.75 (0.94, 2.83) | 2.36 (1.32, 4.38) | 29.90 |
| Myanmar | 0.76 (0.44, 1.36) | 0.62 (0.4, 0.98) | -20.36 |
| Namibia | 6.13 (4.19, 9.06) | 5.45 (3.61, 7.85) | -11.76 |
| Nauru | 1.41 (0.83, 2.34) | 1.09 (0.67, 1.69) | -25.74 |
| Nepal | 1.99 (0.84, 3.86) | 1.88 (0.99, 3.09) | -5.69 |
| Netherlands | 28.49 (17.5, 43.12) | 28.19 (17.62, 44.19) | -1.06 |
| New Zealand | 15 (8.95, 23.95) | 17.87 (10.62, 28.01) | 17.51 |
| Nicaragua | 8.68 (6.54, 12.09) | 8.08 (5.85, 11.02) | -7.16 |
| Niger | 1.06 (0.7, 1.56) | 0.91 (0.61, 1.34) | -15.26 |
| Nigeria | 1.13 (0.77, 1.6) | 1.37 (0.92, 1.94) | 19.26 |
| Niue | 1.29 (0.82, 1.93) | 1.1 (0.76, 1.6) | -15.93 |
| North Macedonia | 5.04 (3.07, 7.86) | 4.72 (2.78, 7.48) | -6.56 |
| Northern Mariana Islands | 1.82 (1.06, 2.63) | 1.01 (0.71, 1.46) | -58.89 |
| Norway | 9.61 (5.99, 14.78) | 19.24 (14.57, 25.66) | 69.42 |
| Oman | 3.68 (2.26, 5.63) | 3.2 (2.12, 4.69) | -13.98 |
| Pakistan | 2.3 (1.09, 3.99) | 2.91 (1.73, 4.41) | 23.52 |
| Palau | 0.4 (0.22, 0.7) | 0.41 (0.23, 0.69) | 2.47 |
| Palestine | 1.84 (1.04, 3.07) | 1.78 (1.02, 2.89) | -3.32 |
| Panama | 17.07 (13.28, 22.13) | 18.76 (14.1, 24.58) | 9.44 |
| Papua New Guinea | 0.75 (0.36, 1.51) | 0.61 (0.32, 1.2) | -20.66 |
| Paraguay | 4.66 (2.84, 7.25) | 8.91 (6.47, 11.94) | 64.82 |
| Peru | 16.77 (12.84, 21.58) | 19.12 (14.26, 25.14) | 13.11 |
| Philippines | 0.86 (0.66, 1.18) | 0.91 (0.68, 1.18) | 5.65 |
| Poland | 9.17 (6.87, 12.74) | 7.32 (4.89, 11) | -22.53 |
| Portugal | 11.89 (8.33, 16.54) | 13.76 (9.97, 18.65) | 14.61 |
| Puerto Rico | 35.14 (25.27, 48.51) | 102.9 (83.89, 123.91) | 107.44 |
| Qatar | 3.25 (2.26, 4.65) | 3 (1.92, 4.34) | -8.00 |
| Republic of Korea | 18.32 (10.94, 29.42) | 17.25 (10.24, 28.36) | -6.02 |
| Republic of Moldova | 13.01 (8.9, 17.61) | 7.64 (5.18, 11.03) | -53.23 |
| Romania | 9.14 (6.62, 12.53) | 6.53 (4.23, 10.14) | -33.63 |
| Russian Federation | 19.93 (17.03, 23.43) | 15.27 (12.19, 19.37) | -26.63 |
| Rwanda | 3.33 (1.71, 7.57) | 2.44 (1.38, 5.02) | -31.10 |
| Saint Kitts and Nevis | 17.49 (11.66, 25.79) | 18.02 (11.72, 26.62) | 2.99 |
| Saint Lucia | 13.74 (8.53, 21.62) | 17.53 (11.64, 25.91) | 24.36 |
| Saint Vincent and the Grenadines | 14.3 (8.67, 22.91) | 19.08 (12.32, 27.95) | 28.84 |
| Samoa | 1 (0.55, 1.68) | 0.92 (0.58, 1.47) | -8.34 |
| San Marino | 15.05 (8.69, 24.75) | 16.27 (9.36, 26.03) | 7.79 |
| Sao Tome and Principe | 1.26 (0.86, 1.78) | 1.8 (1.09, 2.82) | 35.67 |
| Saudi Arabia | 2.11 (1.27, 3.4) | 2.59 (1.58, 4.17) | 20.50 |
| Senegal | 1.05 (0.71, 1.53) | 1.07 (0.72, 1.56) | 1.89 |
| Serbia | 4.91 (3.26, 7.29) | 4.55 (2.85, 7.15) | -7.61 |
| Seychelles | 1.26 (0.9, 1.74) | 1.81 (1.08, 2.54) | 36.22 |
| Sierra Leone | 1.01 (0.68, 1.46) | 1.06 (0.7, 1.57) | 4.83 |
| Singapore | 17.09 (9.74, 28.23) | 17.11 (9.85, 27.74) | 0.12 |
| Slovakia | 11.3 (7.46, 16.96) | 10.05 (6, 15.38) | -11.72 |
| Slovenia | 10.09 (7.32, 14.32) | 8.27 (5.13, 12.57) | -19.89 |
| Solomon Islands | 0.8 (0.36, 1.72) | 0.73 (0.37, 1.47) | -9.16 |
| Somalia | 2.26 (1.08, 4.24) | 2.08 (0.99, 4.23) | -8.30 |
| South Africa | 19.84 (14.52, 27.46) | 20.73 (14.83, 28.18) | 4.39 |
| South Sudan | 2.33 (1.23, 3.9) | 2.39 (1.3, 4.8) | 2.54 |
| Spain | 37.73 (23.51, 56.91) | 39.27 (24.31, 60.21) | 4.00 |
| Sri Lanka | 5.03 (3.43, 7.05) | 3.12 (2, 4.39) | -47.76 |
| Sudan | 7.2 (3.3, 13.82) | 7.61 (4.1, 12.02) | 5.54 |
| Suriname | 16.59 (10.8, 24.68) | 28.6 (20.99, 39.98) | 54.46 |
| Sweden | 9.7 (6.37, 14.35) | 21.66 (16.37, 28.8) | 80.33 |
| Switzerland | 24.16 (16.98, 33.92) | 21.48 (14.58, 30.72) | -11.76 |
| Syrian Arab Republic | 5.03 (3.26, 7.28) | 5.2 (3.57, 7.51) | 3.32 |
| Taiwan (Province of China) | 7.73 (6.88, 8.76) | 0.99 (0.63, 1.56) | -205.52 |
| Tajikistan | 3.38 (2.04, 5.42) | 3.69 (2.17, 5.96) | 8.78 |
| Thailand | 0.88 (0.59, 1.31) | 1.06 (0.75, 1.49) | 18.61 |
| Timor-Leste | 0.6 (0.34, 1.04) | 0.57 (0.36, 0.92) | -5.13 |
| Togo | 1.04 (0.71, 1.52) | 1.07 (0.72, 1.53) | 2.84 |
| Tokelau | 1.26 (0.71, 1.96) | 1.25 (0.86, 1.79) | -0.80 |
| Tonga | 0.83 (0.46, 1.41) | 0.87 (0.55, 1.3) | 4.71 |
| Trinidad and Tobago | 15.42 (9.61, 23.84) | 28.97 (20.93, 39.29) | 63.06 |
| Tunisia | 6.23 (3.24, 10.2) | 8.03 (5.42, 11.99) | 25.38 |
| Türkiye | 5.19 (3.36, 7.4) | 22.68 (6.36, 33.61) | 147.47 |
| Turkmenistan | 1.08 (0.56, 1.97) | 1.06 (0.68, 1.69) | -1.87 |
| Tuvalu | 3.48 (2.35, 5.2) | 3.86 (2.68, 5.46) | 10.36 |
| Uganda | 1.86 (1.05, 3.47) | 2.49 (1.45, 5.53) | 29.17 |
| Ukraine | 7.3 (5.3, 10.43) | 7.16 (5.03, 10.27) | -1.94 |
| United Arab Emirates | 4.82 (3.02, 7.61) | 5.23 (3.69, 7.1) | 8.16 |
| United Kingdom | 28.95 (19.5, 41.94) | 42.79 (31.3, 58.4) | 39.07 |
| United Republic of Tanzania | 2.7 (1.4, 5.77) | 3.2 (1.75, 7.46) | 16.99 |
| United States of America | 56.89 (44.47, 70.94) | 103.59 (72.09, 146.1) | 59.93 |
| United States Virgin Islands | 70.18 (46.98, 101.07) | 154.91 (127.68, 193.23) | 79.18 |
| Uruguay | 34.34 (19.51, 54.94) | 35.75 (21.87, 55.06) | 4.02 |
| Uzbekistan | 3.78 (2.37, 5.95) | 4.97 (3.47, 7.05) | 27.37 |
| Vanuatu | 0.81 (0.4, 1.65) | 0.76 (0.43, 1.35) | -6.37 |
| Venezuela | 6.19 (4.51, 8.56) | 5.46 (3.56, 8.21) | -12.55 |
| Vietnam | 2.07 (1.17, 3.46) | 2.87 (1.65, 5.69) | 32.68 |
| Yemen | 7.1 (3.43, 12.93) | 6.62 (3.11, 11.55) | -7.00 |
| Zambia | 3.24 (1.69, 6.47) | 3.51 (2.01, 6.56) | 8.00 |
| Zimbabwe | 7.07 (4.68, 10.29) | 8.33 (5.22, 11.47) | 16.40 |
