# Supplementary material for: Global burden of disease due to opioid, amphetamine, cocaine, and cannabis use disorders, 1990-2021: a systematic analysis for the Global Burden of Disease Study 2021
Source: PLoS One. 2025 Aug 21;20(8):e0328276. doi: 10.1371/journal.pone.0328276 (PMC12370144; doi:10.1371/journal.pone.0328276)
Supplement: S11 Table — (DOCX) [file pone.0328276.s012.docx]

**S11 Table. Age-standardized disability-adjusted life year (DALY) rates per 100,000 attributable to any cocaine use disorder, stratified by country in 1990 and 2021, and total percentage change**

| **Location** | **DALY rate (95% UI) in 1990** | **DALY rate (95% UI) in 2021** | **% Change** |
| --- | --- | --- | --- |
| Afghanistan | 3.55 (1.94, 6.06) | 3.75 (2.05, 6.24) | 5.48 |
| Albania | 7.67 (3.87, 12.85) | 7.13 (4.04, 11.83) | -7.30 |
| Algeria | 4.09 (2.15, 6.9) | 4.52 (2.55, 7.47) | 10.00 |
| American Samoa | 13.06 (6.75, 22.35) | 13.09 (6.88, 22.74) | 0.23 |
| Andorra | 13.72 (7.13, 23.34) | 13.58 (6.98, 23.07) | -1.03 |
| Angola | 5.18 (2.79, 8.38) | 5.15 (2.81, 8.52) | -0.58 |
| Antigua and Barbuda | 16.84 (9.45, 28.23) | 15.29 (7.66, 25.45) | -9.66 |
| Argentina | 6.53 (3.82, 10.43) | 7.03 (4.63, 10.34) | 7.38 |
| Armenia | 5.75 (3.06, 9.9) | 6.21 (3.42, 10.59) | 7.70 |
| Australia | 37.04 (24.09, 54.25) | 18.36 (12.11, 27.13) | -70.18 |
| Austria | 10.34 (6.04, 16.27) | 11 (6.81, 17.17) | 6.19 |
| Azerbaijan | 5.64 (2.88, 9.91) | 5.79 (2.99, 9.73) | 2.62 |
| Bahamas | 19.35 (11.56, 31.24) | 18.7 (11.24, 31.03) | -3.42 |
| Bahrain | 3.97 (2.16, 6.65) | 4.14 (2.24, 6.99) | 4.19 |
| Bangladesh | 5.41 (3.15, 8.79) | 4.89 (2.95, 7.99) | -10.11 |
| Barbados | 23.17 (13.45, 38.64) | 23.18 (14.42, 35.82) | 0.04 |
| Belarus | 7.86 (3.94, 13.51) | 7.95 (4.11, 13.32) | 1.14 |
| Belgium | 17.05 (10.64, 26.81) | 14.98 (9.63, 23.15) | -12.94 |
| Belize | 19.54 (11.99, 30.13) | 17.63 (10.31, 29.36) | -10.29 |
| Benin | 4.21 (2.25, 6.8) | 4.69 (2.68, 7.67) | 10.80 |
| Bermuda | 15.03 (7.95, 25.21) | 15.03 (7.87, 24.93) | 0.00 |
| Bhutan | 6.47 (3.66, 10.79) | 6.48 (3.61, 10.72) | 0.15 |
| Bolivia | 7.08 (4.19, 11.08) | 7.79 (4.64, 12.48) | 9.56 |
| Bosnia and Herzegovina | 6.41 (3.2, 10.64) | 5.25 (2.97, 8.83) | -19.96 |
| Botswana | 4.78 (2.57, 7.87) | 4.89 (2.72, 8.12) | 2.28 |
| Brazil | 14.11 (8.07, 22.55) | 12.33 (7.4, 19.44) | -13.48 |
| Brunei Darussalam | 13.27 (6.93, 22.53) | 13.33 (7.08, 22.94) | 0.45 |
| Bulgaria | 8.39 (5.3, 12.76) | 10.27 (6.41, 15.52) | 20.22 |
| Burkina Faso | 3.39 (1.77, 5.51) | 3.32 (1.88, 5.33) | -2.09 |
| Burundi | 6.09 (3.18, 10.14) | 6.16 (3.35, 10.1) | 1.14 |
| Cabo Verde | 3.56 (1.84, 5.97) | 3.76 (1.95, 6.32) | 5.47 |
| Cambodia | 7.01 (3.69, 11.84) | 6.36 (3.59, 10.53) | -9.73 |
| Cameroon | 3.61 (1.93, 5.93) | 3.66 (1.82, 6.22) | 1.38 |
| Canada | 35.76 (23.51, 52.82) | 30.16 (19.83, 43.48) | -17.03 |
| Central African Republic | 5.1 (2.79, 8.3) | 5.13 (2.76, 8.8) | 0.59 |
| Chad | 3.59 (1.93, 5.9) | 3.62 (1.92, 6.09) | 0.83 |
| Chile | 11.27 (7.33, 16.62) | 19.3 (12.8, 27.62) | 53.80 |
| China | 4.83 (2.81, 7.73) | 6.03 (3.46, 9.67) | 22.19 |
| Colombia | 8.59 (5.29, 13.1) | 16.68 (10.64, 24.46) | 66.36 |
| Comoros | 6.14 (3.15, 10.45) | 6.22 (3.12, 10.52) | 1.29 |
| Congo | 5.16 (2.7, 8.71) | 5.19 (2.86, 8.71) | 0.58 |
| Cook Islands | 13.15 (6.77, 22.61) | 12.81 (6.71, 22.04) | -2.62 |
| Costa Rica | 8.39 (4.68, 14.27) | 8.88 (5.41, 13.94) | 5.68 |
| Côte d'Ivoire | 3.68 (2.09, 6.14) | 3.72 (1.96, 6.22) | 1.08 |
| Croatia | 13.58 (8.06, 21.42) | 12.84 (8, 19.62) | -5.60 |
| Cuba | 15.12 (7.79, 25.44) | 15.15 (7.97, 25.55) | 0.20 |
| Cyprus | 8.62 (4.93, 14.11) | 6.49 (3.99, 10.42) | -28.38 |
| Czechia | 19.61 (12.09, 30.48) | 22.29 (13.76, 34.54) | 12.81 |
| Democratic People's Republic of Korea | 5.48 (3, 8.89) | 5.54 (3.07, 9.39) | 1.09 |
| Republic of the Congo | 13.35 (8.1, 20.86) | 11.09 (6.78, 17.44) | -18.55 |
| Denmark | 6.38 (3.35, 10.84) | 6.42 (3.31, 10.82) | 0.63 |
| Djibouti | 24.67 (13.89, 41.63) | 20.3 (11.33, 33.84) | -19.50 |
| Dominica | 8.54 (4.94, 13.82) | 11.07 (5.54, 18.89) | 25.95 |
| Dominican Republic | 8.31 (4.64, 13.03) | 7.97 (4.44, 13.01) | -4.18 |
| Ecuador | 2.87 (1.61, 4.98) | 3.28 (1.76, 5.5) | 13.35 |
| Egypt | 4.33 (2.53, 6.7) | 5.39 (2.85, 8.82) | 21.90 |
| El Salvador | 4.99 (2.63, 8.24) | 5.42 (2.86, 8.95) | 8.27 |
| Equatorial Guinea | 6.04 (3.14, 10.19) | 6.26 (3.29, 10.2) | 3.58 |
| Eritrea | 11.77 (7.23, 18.52) | 11.06 (6.97, 16.87) | -6.22 |
| Estonia | 6.1 (3.5, 9.78) | 6.85 (4.07, 10.81) | 11.60 |
| Eswatini | 6.25 (3.51, 10.04) | 6.39 (3.58, 10.49) | 2.22 |
| Ethiopia | 13.05 (6.89, 22.8) | 13.07 (6.65, 22.34) | 0.15 |
| Fiji | 9.86 (5.7, 15.64) | 9.44 (5.79, 14.54) | -4.35 |
| Finland | 20.47 (12.88, 30.55) | 20.5 (13.09, 31.69) | 0.15 |
| France | 5.25 (2.89, 8.63) | 5.1 (2.65, 8.52) | -2.90 |
| Gabon | 3.67 (1.96, 6.05) | 3.63 (2, 6.11) | -1.10 |
| Gambia | 3.65 (2.02, 6.28) | 4.44 (2.38, 7.76) | 19.59 |
| Georgia | 10.67 (6.55, 15.85) | 13.43 (8.56, 19.6) | 23.01 |
| Germany | 3.75 (2.01, 6.3) | 4.14 (2.35, 6.79) | 9.89 |
| Ghana | 7.19 (4.42, 11.05) | 7.97 (4.83, 12.77) | 10.30 |
| Greece | 27.46 (17.48, 40.8) | 25.49 (15.56, 39.87) | -7.44 |
| Greenland | 17.01 (9.66, 29.25) | 15.09 (8.05, 25.82) | -11.98 |
| Grenada | 13.37 (6.9, 22.99) | 13.19 (6.8, 22.67) | -1.36 |
| Guam | 3.97 (2.32, 6.17) | 5.19 (2.85, 8.55) | 26.80 |
| Guatemala | 3.59 (1.97, 5.93) | 3.6 (1.86, 6.02) | 0.28 |
| Guinea | 3.58 (1.96, 5.89) | 3.59 (1.94, 6.08) | 0.28 |
| Guinea-Bissau | 11.51 (6.86, 18.27) | 11.91 (6.22, 19.8) | 3.42 |
| Guyana | 11.38 (6.17, 19.39) | 13.43 (7.22, 23.56) | 16.56 |
| Haiti | 7.49 (4.41, 12.4) | 8.2 (4.41, 13.84) | 9.06 |
| Honduras | 9.48 (5.8, 14.79) | 7.99 (4.94, 12.25) | -17.10 |
| Hungary | 10.32 (5.92, 16.61) | 9.5 (5.45, 15.33) | -8.28 |
| Iceland | 7.93 (4.67, 12.55) | 7.88 (4.69, 12.58) | -0.63 |
| India | 6.4 (3.7, 10.25) | 7.45 (4.31, 12.04) | 15.19 |
| Indonesia | 3.46 (1.98, 5.53) | 5.52 (3.14, 8.85) | 46.71 |
| Iran | 3.92 (2.12, 6.62) | 4.26 (2.45, 7.12) | 8.32 |
| Iraq | 19.76 (12.54, 29.53) | 14.79 (9.54, 22.11) | -28.97 |
| Ireland | 9.44 (6, 13.72) | 9.25 (5.63, 14.37) | -2.03 |
| Israel | 20.56 (11.62, 33.56) | 13.2 (7.42, 21.09) | -44.31 |
| Italy | 15.87 (9.09, 26.17) | 18.14 (11.04, 28.88) | 13.37 |
| Jamaica | 13.92 (8.06, 22.43) | 13.74 (8.01, 22.58) | -1.30 |
| Japan | 3.86 (2.01, 6.69) | 3.88 (2.08, 6.49) | 0.52 |
| Jordan | 5.67 (3, 9.55) | 5.68 (2.93, 9.66) | 0.18 |
| Kazakhstan | 3.68 (2.14, 5.8) | 4.91 (2.84, 7.92) | 28.84 |
| Kenya | 12.58 (6.63, 21.33) | 13.29 (7.48, 22) | 5.49 |
| Kiribati | 4.48 (2.39, 7.49) | 5.06 (2.79, 8.32) | 12.17 |
| Kuwait | 5.66 (2.87, 9.72) | 5.71 (2.95, 9.62) | 0.88 |
| Kyrgyzstan | 8 (4.27, 13.6) | 8.1 (4.13, 13.85) | 1.24 |
| Lao People's Democratic Republic | 10.52 (6.63, 16.38) | 10.01 (6.27, 15.43) | -4.97 |
| Latvia | 4.04 (2.25, 6.7) | 4.93 (2.71, 8.18) | 19.91 |
| Lebanon | 5.73 (2.91, 9.63) | 6.07 (3.15, 10.26) | 5.76 |
| Lesotho | 4.42 (2.36, 7.35) | 5.32 (2.96, 8.79) | 18.53 |
| Liberia | 3.83 (2.01, 6.58) | 3.77 (2.05, 6.38) | -1.58 |
| Libya | 6.93 (4.11, 10.93) | 7.71 (4.9, 12.01) | 10.67 |
| Lithuania | 15.79 (8.78, 25.6) | 16.23 (9.63, 26.28) | 2.75 |
| Luxembourg | 6.12 (3.17, 10.14) | 6.11 (3.28, 10.3) | -0.16 |
| Madagascar | 6.08 (3.22, 10.1) | 6.18 (3.33, 10.29) | 1.63 |
| Malawi | 6.92 (3.8, 11.54) | 6.36 (3.73, 10.4) | -8.44 |
| Malaysia | 8.08 (4.46, 13.84) | 8.38 (4.55, 14.06) | 3.65 |
| Maldives | 3.57 (1.88, 5.75) | 3.64 (2.04, 6.17) | 1.94 |
| Mali | 8.11 (4.77, 12.29) | 10 (5.99, 15.5) | 20.95 |
| Malta | 13 (6.78, 22.45) | 13.06 (6.74, 22.52) | 0.46 |
| Marshall Islands | 3.61 (1.94, 5.99) | 3.63 (1.91, 6.11) | 0.55 |
| Mauritania | 8.97 (4.92, 14.76) | 10.6 (6.29, 16.81) | 16.70 |
| Mauritius | 5.69 (3.28, 8.98) | 5.59 (3.44, 8.53) | -1.77 |
| Mexico | 13 (6.89, 22.39) | 13.1 (6.84, 22.38) | 0.77 |
| Micronesia | 13.44 (6.88, 23.12) | 13.39 (6.87, 22.93) | -0.37 |
| Monaco | 6.95 (3.44, 11.99) | 8.27 (4.74, 13.9) | 17.39 |
| Mongolia | 7.91 (4.39, 12.71) | 6.37 (3.53, 10.67) | -21.65 |
| Montenegro | 3.89 (1.99, 6.52) | 4.42 (2.52, 7.35) | 12.77 |
| Morocco | 5.93 (3.22, 9.93) | 6 (3.14, 10.18) | 1.17 |
| Mozambique | 9.35 (5.27, 15.53) | 8.99 (4.67, 15.2) | -3.93 |
| Myanmar | 6.02 (3.22, 10.04) | 7.14 (4.13, 11.65) | 17.06 |
| Namibia | 13.01 (6.71, 22.75) | 13.14 (6.8, 22.08) | 0.99 |
| Nauru | 6.31 (3.63, 10.5) | 6.24 (3.42, 10.4) | -1.12 |
| Nepal | 19.43 (12.55, 29.15) | 13.66 (8.37, 21.18) | -35.23 |
| Netherlands | 26.29 (15.62, 42.66) | 31.45 (18.3, 51.44) | 17.92 |
| New Zealand | 6.15 (3.61, 9.65) | 4.69 (2.71, 7.74) | -27.10 |
| Nicaragua | 3.64 (1.92, 6.16) | 3.65 (2.01, 6.16) | 0.27 |
| Niger | 3.47 (2.02, 5.45) | 3.5 (2.03, 5.6) | 0.86 |
| Nigeria | 13.29 (6.76, 22.8) | 13.18 (6.86, 22.9) | -0.83 |
| Niue | 5.91 (3.39, 9.37) | 5.42 (3.18, 9) | -8.66 |
| North Macedonia | 12.96 (6.81, 22.13) | 13.19 (6.9, 22.89) | 1.76 |
| Northern Mariana Islands | 9.69 (5.73, 15.67) | 8.99 (5.28, 14.97) | -7.50 |
| Norway | 4.08 (2.19, 7.13) | 4.02 (2.19, 6.73) | -1.48 |
| Oman | 7.05 (4.05, 10.92) | 6.98 (4.17, 11.01) | -1.00 |
| Pakistan | 13.25 (6.82, 23.21) | 13.45 (7.03, 23.16) | 1.50 |
| Palau | 4.39 (2.34, 7.53) | 5.35 (3.07, 9.29) | 19.78 |
| Palestine | 11.74 (6.97, 18.08) | 9.36 (4.95, 16.21) | -22.66 |
| Panama | 13.06 (6.81, 23.04) | 13.11 (6.97, 22.19) | 0.38 |
| Papua New Guinea | 5.96 (3.54, 9.31) | 5.97 (3.27, 9.98) | 0.17 |
| Paraguay | 6.5 (3.74, 10.5) | 5.97 (3.46, 9.86) | -8.51 |
| Peru | 8.37 (4.77, 13.25) | 8.43 (4.73, 13.51) | 0.71 |
| Philippines | 13.13 (6.96, 21.76) | 11.44 (6.34, 19) | -13.78 |
| Poland | 12.9 (8.01, 19.49) | 9.67 (5.97, 14.8) | -28.82 |
| Portugal | 14.82 (7.79, 25.04) | 14.95 (7.88, 24.8) | 0.87 |
| Puerto Rico | 4.26 (2.19, 7.32) | 4.28 (2.25, 7.3) | 0.47 |
| Qatar | 13.23 (6.93, 22.31) | 13.27 (6.94, 22.9) | 0.30 |
| Republic of Korea | 6.12 (3.22, 10.8) | 5.5 (3.15, 9.51) | -10.68 |
| Republic of Moldova | 5.11 (2.76, 8.51) | 5.24 (2.88, 8.76) | 2.51 |
| Romania | 4.66 (2.62, 7.66) | 4.54 (2.71, 6.98) | -2.61 |
| Russian Federation | 10.13 (5.8, 16.44) | 10.95 (5.99, 17.4) | 7.78 |
| Rwanda | 7.07 (3.57, 11.81) | 8.74 (4.7, 14.52) | 21.20 |
| Saint Kitts and Nevis | 14.99 (7.88, 25.4) | 15.04 (7.92, 25.39) | 0.33 |
| Saint Lucia | 26.04 (15.34, 41.12) | 19.53 (10.27, 32.55) | -28.77 |
| Saint Vincent and the Grenadines | 15.08 (7.83, 26.19) | 16.96 (9.82, 27.94) | 11.75 |
| Samoa | 16.37 (8.81, 27.81) | 20.79 (12.61, 32.73) | 23.90 |
| San Marino | 13.34 (6.99, 22.43) | 13.44 (6.71, 23.4) | 0.75 |
| Sao Tome and Principe | 3.66 (2, 6.16) | 3.72 (2.08, 6.28) | 1.63 |
| Saudi Arabia | 3.99 (2.23, 6.74) | 4.02 (1.99, 6.85) | 0.75 |
| Senegal | 3.6 (1.96, 5.95) | 3.66 (1.96, 6.15) | 1.65 |
| Serbia | 7.89 (4.38, 13.2) | 6.2 (3.69, 9.51) | -24.10 |
| Seychelles | 16.07 (8.88, 26.92) | 12.86 (7.13, 21.45) | -22.28 |
| Sierra Leone | 3.6 (1.96, 6.08) | 3.65 (1.98, 6.26) | 1.38 |
| Singapore | 13.33 (6.8, 23.03) | 13.26 (6.91, 21.93) | -0.53 |
| Slovakia | 11.12 (6.6, 17.62) | 10.64 (6.95, 16.09) | -4.41 |
| Slovenia | 17.32 (10.19, 27.15) | 13.44 (8.72, 20.31) | -25.36 |
| Solomon Islands | 12.16 (6.44, 20.36) | 12.7 (7.45, 20.85) | 4.35 |
| Somalia | 6.26 (3.32, 10.56) | 6.24 (3.42, 10.63) | -0.32 |
| South Africa | 7.83 (4.67, 12.15) | 9.33 (5.28, 15.22) | 17.53 |
| South Sudan | 6.29 (3.18, 10.55) | 6.08 (3.22, 10.11) | -3.40 |
| Spain | 20.74 (13.94, 28.84) | 25.6 (16.92, 36.24) | 21.05 |
| Sri Lanka | 8.04 (4.09, 13.75) | 8.04 (4.35, 13.72) | 0.00 |
| Sudan | 3.72 (1.98, 6.52) | 3.78 (1.96, 6.28) | 1.60 |
| Suriname | 12.83 (6.87, 21.78) | 12.1 (6.87, 19.6) | -5.86 |
| Sweden | 9.58 (5.61, 15.92) | 7.23 (4.06, 11.96) | -28.14 |
| Switzerland | 27.72 (16.94, 44.33) | 22.01 (13.78, 34.29) | -23.07 |
| Syrian Arab Republic | 2.84 (1.5, 4.78) | 3.09 (1.6, 5.14) | 8.44 |
| Taiwan (Province of China) | 5.55 (3.06, 9.21) | 5.54 (3.02, 9.38) | -0.18 |
| Tajikistan | 5.67 (2.91, 9.78) | 5.73 (2.99, 9.46) | 1.05 |
| Thailand | 8.04 (4.36, 13.64) | 8.35 (4.76, 13.69) | 3.78 |
| Timor-Leste | 8.08 (4.23, 13.74) | 8.09 (4.42, 13.49) | 0.12 |
| Togo | 2.98 (1.61, 4.86) | 2.78 (1.6, 4.47) | -6.95 |
| Tokelau | 13.12 (6.87, 22.32) | 13.13 (6.81, 22.17) | 0.08 |
| Tonga | 13.31 (6.71, 22.75) | 14.56 (8.13, 24.34) | 8.98 |
| Trinidad and Tobago | 12.84 (6.56, 21.97) | 13.16 (7.57, 21.83) | 2.46 |
| Tunisia | 3.78 (1.93, 6.61) | 3.74 (2.01, 6.33) | -1.06 |
| Türkiye | 2.83 (1.65, 4.55) | 2.4 (1.37, 3.79) | -16.48 |
| Turkmenistan | 5.69 (2.92, 9.96) | 5.79 (3.12, 9.92) | 1.74 |
| Tuvalu | 12.9 (6.57, 22.57) | 13.26 (6.89, 22.66) | 2.75 |
| Uganda | 8.08 (4.44, 13.71) | 7.44 (4.28, 11.6) | -8.25 |
| Ukraine | 7.02 (3.75, 11.61) | 6.59 (3.72, 10.78) | -6.32 |
| United Arab Emirates | 4.17 (2.13, 7.09) | 4.06 (2.13, 6.69) | -2.67 |
| United Kingdom | 26.67 (15.87, 42.24) | 25.65 (15.2, 40.77) | -3.90 |
| United Republic of Tanzania | 7.84 (4.64, 12.69) | 6.57 (3.47, 11.12) | -17.67 |
| United States of America | 28.88 (17.15, 44.93) | 27.69 (16.62, 43.19) | -4.21 |
| United States Virgin Islands | 14.87 (7.76, 24.59) | 14.96 (7.86, 25.02) | 0.60 |
| Uruguay | 14.38 (8.54, 22.69) | 14.19 (8.96, 21.73) | -1.33 |
| Uzbekistan | 5.67 (3.02, 9.84) | 5.7 (2.86, 9.59) | 0.53 |
| Vanuatu | 11.43 (5.86, 20.41) | 11.31 (6.52, 18.01) | -1.06 |
| Venezuela | 6.43 (3.6, 10.63) | 5.86 (3.32, 9.67) | -9.28 |
| Vietnam | 8.08 (4.3, 14.21) | 8.14 (4.26, 13.6) | 0.74 |
| Yemen | 3.74 (2, 6.53) | 3.75 (2.04, 6.35) | 0.27 |
| Zambia | 7.05 (4.18, 10.99) | 5 (2.86, 8.23) | -34.36 |
| Zimbabwe | 6.05 (3.26, 10.46) | 6.02 (3.24, 10.04) | -0.50 |
